# The Association between Genetics and Response to Treatment with Biologics in Patients with Psoriasis, Psoriatic Arthritis, Rheumatoid Arthritis, and Inflammatory Bowel Diseases: A Systematic Review and Meta-Analysis

**DOI:** 10.3390/ijms25115793

**Published:** 2024-05-26

**Authors:** Rownaq Fares Al-Sofi, Mie Siewertsen Bergmann, Claus Henrik Nielsen, Vibeke Andersen, Lone Skov, Nikolai Loft

**Affiliations:** 1 Department of Dermatology and Allergy, Copenhagen University Hospital—Herlev and Gentofte, 1165 Copenhagen, Denmark; 2 Copenhagen Research Group for Inflammatory Skin, Herlev and Gentofte Hospital, 2730 Herlev, Denmark; 3 Center for Rheumatology and Spine Diseases, Institute for Inflammation Research, Copenhagen University Hospital Rigshospitalet, 2100 Copenhagen, Denmark; 4 Institute of Regional Health Research, University of Southern Denmark, 5230 Odense, Denmark; 5 Molecular Diagnostics and Clinical Research Unit, Department of Internal Medicine, University Hospital of Southern Denmark, 6200 Aabenraa, Denmark; 6 Institute of Molecular Medicine, University of Southern Denmark, 5230 Odense, Denmark; 7 Department of Clinical Medicine, Faculty of Health and Medical Sciences, University of Copenhagen, 1172 Copenhagen, Denmark

**Keywords:** biomarkers, psoriasis, psoriatic arthritis, rheumatoid arthritits, Crohns, ulcerative colitis, inflammatory bowel diseases, pharmacogenetics

## Abstract

Genetic biomarkers could potentially lower the risk of treatment failure in chronic inflammatory diseases (CID) like psoriasis, psoriatic arthritis (PsA), rheumatoid arthritis (RA), and inflammatory bowel disease (IBD). We performed a systematic review and meta-analysis assessing the association between single nucleotide polymorphisms (SNPs) and response to biologics. Odds ratio (OR) with 95% confidence interval (CI) meta-analyses were performed. In total, 185 studies examining 62,774 individuals were included. For the diseases combined, the minor allele of MYD88 (rs7744) was associated with good response to TNFi (OR: 1.24 [1.02–1.51], 6 studies, 3158 patients with psoriasis or RA) and the minor alleles of NLRP3 (rs4612666) (OR: 0.71 [0.58–0.87], 5 studies, 3819 patients with RA or IBD), TNF-308 (rs1800629) (OR: 0.71 [0.55–0.92], 25 studies, 4341 patients with psoriasis, RA, or IBD), FCGR3A (rs396991) (OR: 0.77 [0.65–0.93], 18 studies, 2562 patients with psoriasis, PsA, RA, or IBD), and TNF-238 (rs361525) (OR: 0.57 [0.34–0.96]), 7 studies, 818 patients with psoriasis, RA, or IBD) were associated with poor response to TNFi together or infliximab alone. Genetic variants in TNFα, NLRP3, MYD88, and FcRγ genes are associated with response to TNFi across several inflammatory diseases. Most other genetic variants associated with response were observed in a few studies, and further validation is needed.

## 1. Introduction

Psoriasis, psoriatic arthritis (PsA), rheumatoid arthritis (RA), and inflammatory bowel disease (IBD) are chronic inflammatory diseases (CID) with reported prevalence ranging from 0.13% for PsA to up to 4% for psoriasis [1,2]. These diseases exhibit genetic and immunological parallels, predominantly driven by T helper (Th)-cell responses [3,4,5,6,7,8]. A shared characteristic among these conditions is their immunological pathogenesis, marked by an excessive activity of specific components within the immune system. Elevated levels of cytokines such as tumor necrosis factor (TNF)-α, interleukin (IL)-1, IL-6, IFN-γ, IL-12/-23, IL-17, and IL-23 play a pivotal role in driving the inflammation associated with these diseases [9,10,11]. Treatment of these inflammatory diseases is often done according to severity. Moderate-to-severe cases often require systemic treatment, including disease-modifying anti-rheumatic drugs (DMARDs), and prednisolone, and some cases require treatment with biologics, also referred to as biological DMARDs (bDMARDs) [12]. Biologics are drugs targeting specific parts of the immune system. For these diseases, most biologics target specific cytokines that are important in immunopathogenesis. The most frequently used biologics are drugs inhibiting TNF-α (TNFi), and these are used across all mentioned diseases [12,13]. Other biologics include IL-6 inhibitors, T-cell co-stimulation blockers, and CD20 antibodies used for RA, IL-17 and IL-23 inhibitors for psoriasis and PsA, and IL-12/-23 inhibitors used for psoriasis, PsA and IBD [14,15,16,17].

Biologics have revolutionized the treatment of these diseases, although approximately 30% of patients do not respond to treatment, requiring a switch in biologics or treatment approach [18]. Today, no biomarkers are available to identify treatment for individual patients, although different biomarkers have been proposed. Indeed, high CRP early in treatment has been associated with the risk of colectomy in patients with ulcerative colitis [19], serum drug concentration has been associated with response [20], certain cytokines have been associated with response [21,22,23], and different genetic variants have been associated with response [24,25,26,27,28,29,30,31,32,33]. In general, these genetic markers have been assessed in smaller studies, which by themselves might not be powered for identifying an association, and most have only been assessed in a few studies [24,25,26,27,28,29,30,31,32,33]. In this study, we conducted a systematic review and meta-analysis on single nucleotide polymorphism (SNP) genetic markers and their response to biologics in psoriasis, PsA, RA, and IBD, and across all the CIDs due to shared immunology.

## 2. Methods and Materials

**Figure 1:**
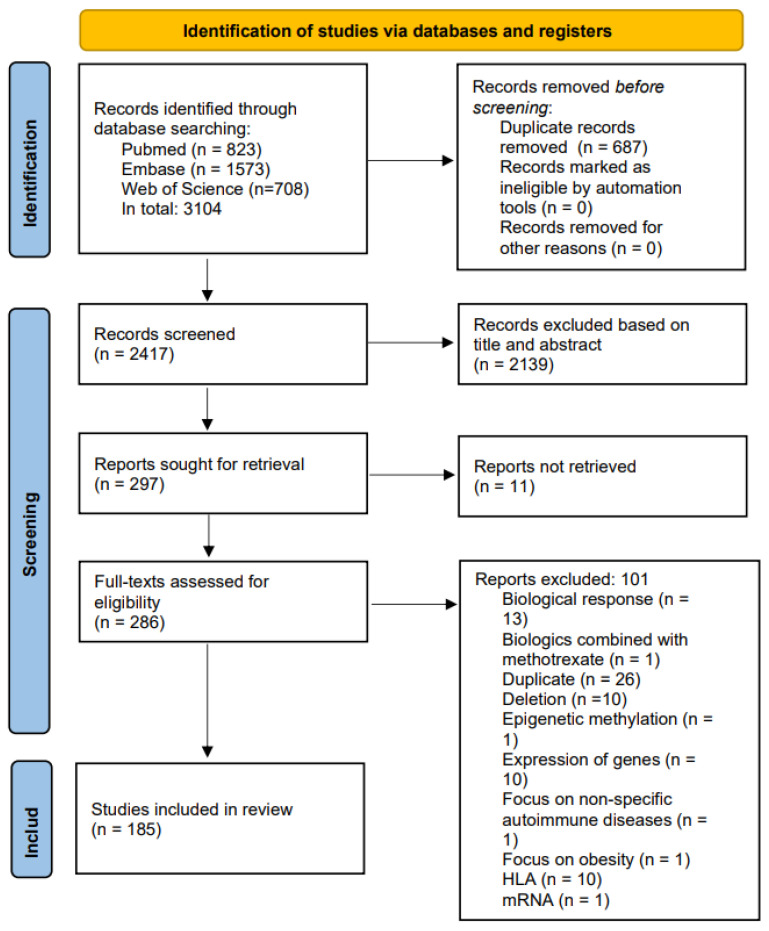
Prisma flowchart.

A systematic review and meta-analysis were conducted according to the guidelines of the Preferred Reporting Items for systematic Reviews and Meta-analyses (PRISMA) statement (Figure 1). Prior to the study, the protocol for the systematic review was registered in PROSPERO (CRD42021282400).

### 2.1. Eligibility Criteria

Published studies in English from any year and any healthcare setting were eligible to be included. All original study types in patients with psoriasis, PsA, RA, or IBD treated with biologics and any SNPs outside the HLA region predicting response were included. Studies had to report an association between SNPs and response/non-response to treatment with biologics. Studies had to be original and report the number of patients receiving the given biologic, the number and/or proportion of patients achieving response, and the number and/or proportion of patients with the SNP and response to treatment. If only haplotypes were presented, the study was excluded.

### 2.2. Search Strategy

Two authors (RA, MS) independently screened Pubmed, Embase, and Web of Science. The databases were screened from inception through November 2021 using numerous alternative search terms for (i) ”biologics”, (ii) “psoriasis, rheumatoid arthritis, inflammatory bowel disease”, and (iii) ”polymorphism” (Supplementary Table S1). Screening was conducted using the web-based screening tool Rayyan, which allows collaborative blinded citation screening [34]. In Rayyan, users assessed studies for in- or exclusion blinded to the other screeners. Following unblinding, disagreements between screeners were shown. Disagreements were resolved through debate, and the resulting decision was unanimous. If the reviewers could not come to an agreement, the senior author (NL) would make the final decision. Data on drug type, number of participants, age, SNP, assessment of response, time of response, and association with response were extracted from each article.

### 2.3. Statistics

Data synthesis was performed using StatsDirect version 3 (StatsDirect Ltd., Cheshire, UK). All SNPs that were found to be associated with a response to biologics in any of the inflammatory diseases were included (Supplementary Tables S2–S5). Meta-analyses were performed for SNPs assessed in ≥ two studies assessing the same drug and/or drug class for the individual disease with retrievable data on genotypes and treatment response. An odds ratio [OR] meta-analysis was performed to achieve pooled OR using the random effects model (Der Simonian and Laird) due to the substantial heterogeneity of studies. Cochran’s Q-test was used to assess the heterogeneity of the studies using a significance level of 0.05 and I2 (inconsistency) statistics. Analyses were conducted for the individual diseases and for SNPs, which could be included in ≥2 meta-analyses for individual diseases for the same drug and/or drug class pooled meta-analyses across all CIDs were conducted. UC and Crohn’s disease were categorized together as IBD as this was done in several of the included studies. Odds ratios were calculated for the odds of achieving a response in those with minor alleles compared with those with major alleles. The response was assessed according to criteria set in the study, and only binary response outcomes (i.e., responders/non-responders) were included. If a study reported multiple categorical outcomes (e.g., non-responders, intermediate responders, and good responders), the responders categories were pooled to be non-responders vs. all other types of responders unless otherwise specified in the study. If a study reported a response for multiple time points, the one with the most comprehensive data was selected. Each SNP was categorized according to drug class, treatment response, and the distribution of responders and non-responders according to minor and major alleles. Minor alleles were categorized according to the National Center of Biotechnology Information in the National Institute of Health (NCBI, NIH), unless otherwise specified.

## 3. Results

In total, 3104 studies were assessed, of which 267 were included for full-text screening. Of these, 185 studies comprising a total of 62,774 patients met the prespecified inclusion criteria and were included in the systematic review (Figure 1). Of the 185 studies, 32 (17.3%) included patients with psoriasis, 7 (3.8%) included patients with PsA, 100 (54.1%) included patients with RA, and 46 (24.9%) included patients with IBD (Supplementary Tables S2–S5).

### 3.1. Association between SNPs and Response to Biologics in Psoriasis

In total, 32 studies including 4413 patients assessed the association between genetic variants and response to biologics in patients with psoriasis. Of the assessed genetic variants, 126 have been associated with response to treatment with biologics among patients with psoriasis. Most SNPs were assessed in patients treated with TNFi, and most SNPs were only assessed in one study (Supplementary Table S2).

#### 3.1.1. Association between SNPs and Response to TNFi in Psoriasis

In total, 27 studies including 3647 patients assessed the association between genetic variants and response to treatment with TNFi for patients with psoriasis. Of the assessed genetic variants, 118 across 85 genes have been associated with response to treatment with TNFi (Supplementary Table S2). The SNPs assessed several times were primarily in the genes linked to the working mechanism of the TNFi where SNPs in the TNF-α gene, TNF-α receptor, and TNFAIP3 were the most frequently investigated.

**Figure 2:**
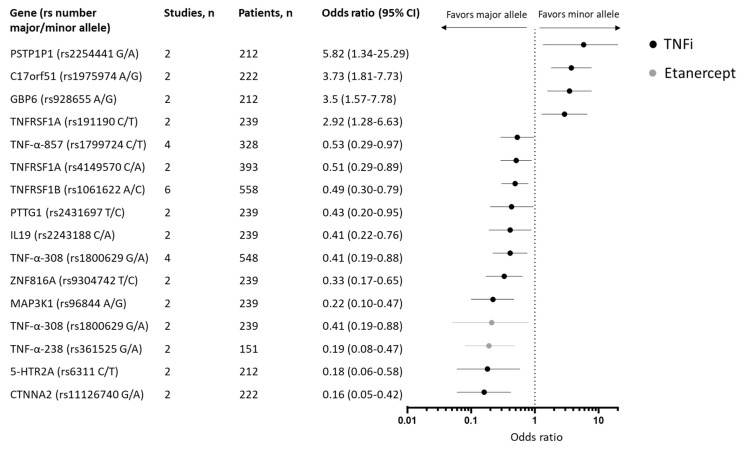
Genetic variants significantly associated with response to biologics among patients with psoriasis in the meta-analysis.

Nineteen studies (n = 2.294 patients) were included in the meta-analysis, and 29 genetic variants were assessed, of which 15 were associated with response in the meta-analysis (Supplementary Table S6). The strongest associations were observed for SNPs in the CTNNA2, 5-HTR2A, and TNFα genes (Figure 2). The minor alleles of CTNNA2 (rs11126740) (two studies, n = 222 patients [32,35]) and 5-HTR2A (rs6311) (two studies, n = 212 patients [32,35]) were associated with poor response to treatment with TNFi (OR: 0.16 [95% CI: 0.06 to 0.42] and OR: 0.18 [95% CI: 0.06–0.58], respectively). The minor allele of PSTP1P1 (rs2254441) (two studies, n = 239 patients [32,35]) was associated with good response (OR: 5.82 [95% CI: 1.34–25.29]).

Several SNPs in the TNF-α gene have been assessed and found to be associated with response. In the meta-analysis, the minor allele of TNF-α-238 (rs361525) was found to be associated with poor response to etanercept (OR: 0.19 [95% CI: 0.08–0.47]) based on two studies (n = 151 patients [36,37]) but not when assessing all TNFi together. The minor alleles of TNF-α-308 (rs1800629) (four studies, n = 548 patients [24,36,37,38]) and TNF-α-857 (rs1799724) (four studies (n = 328 patients [36,37,39,40]) were associated with poor response to treatment with TNFi (OR: 0.41 [95% CI: 0.19–0.88], and OR: 0.53 [95% CI: 0.29–0.97], respectively). The association between SNPs in TNF receptors and response to TNFi was assessed in multiple studies. Of these, the minor allele of TNFRSF1A (rs191190) was associated with good response to TNFi (OR: 2.86 [95% CI: 1.25–6.52]) based on two studies and 239 patients [32,41], while the minor alleles of TNFRSF1B (rs1061622) (six studies, n = 55,832, [38,39,40,41,42]) and TNFRSF1A (rs4149570) two studies, n = 23,932, [41]) were associated with poor response to treatment with TNFi (OR: 0.49 [95% CI: 0.30–0.79] and OR: 0.51 [95% 0.29–0.89], respectively).

#### 3.1.2. Association between SNPs and Response to IL-12/-23i in Psoriasis

In total, seven studies including 572 patients assessed the association between genetic variants and response to treatment of psoriasis with IL-12/23i. Of the assessed genetic variants, 44 SNPs across 36 genes have been associated with response or lack of response to IL-12/23i. Four studies (n = 416 patients) and four genetic variants were assessed in the meta-analysis, none of which were associated with a response to treatment with IL-12/23i (Supplementary Table S6).

#### 3.1.3. Association between SNPs and Response to IL-17i in Psoriasis

Two studies including 194 patients assessed the association between genetic variants and response to treatment with IL-17i. Here, TYK2 (rs2304255), DDX58_v1 (rs34085293), and MICB-DT (rs9267325) were associated with response [43], while IL-17A (rs2275913) was not associated with response [44].

### 3.2. Association between SNPs and Response to Biologics in Psoriatic Arthritis

In total, seven studies including 469 patients assessed the association between genetic variants and response to biologics in patients with PsA. Of the genetic variants, nine SNPs across eight genes have been associated with response (Supplementary Table S3). All variants were assessed in patients treated with TNFi. Only FCGR2A (rs1801274) could be included in the meta-analysis, and no association with a response (OR: 3.29 [95% CI: 0.20–52.96]) was observed, including two studies (n = 114 patients [45,46]).

### 3.3. Association between SNPs and Response to Biologics in Rheumatoid Arthritis

In total, 100 studies including 42,886 patients assessed the association between SNPs and response to biologics in RA. Of the assessed genetic variants, 232 have been associated with response to treatment with biologics among patients with RA of which most were assessed for TNFi and most were only assessed in one study (Supplementary Table S4).

#### 3.3.1. Association between SNPs and Response to TNFi in Rheumatoid Arthritis

In total, 77 studies including 38,225 patients assessed the association between genetic variants and response to treatment with TNFi in patients with RA. Of the genetic variants, 211 across 162 genes were associated with response to TNFi (Supplementary Table S4).

The SNPs assessed were primarily in the genes linked to the working mechanisms of the therapeutic targets or in mechanisms important for the RA pathogenesis.

**Figure 3:**
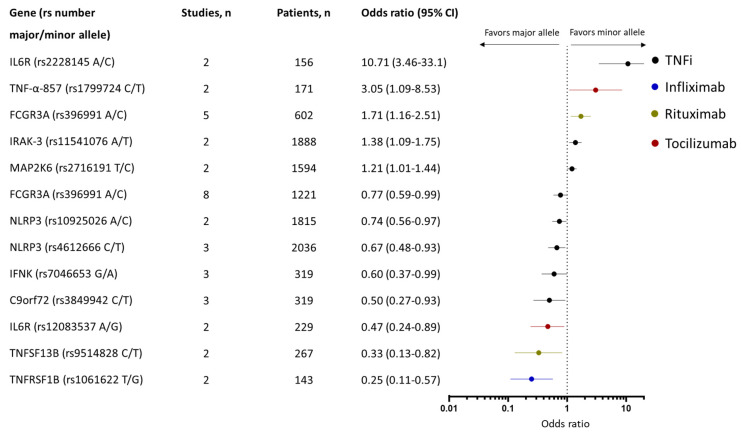
Genetic variants significantly associated with response to biologics among patients with rheumatoid arthritis in the meta-analysis.

In total, 64 studies were included in the meta-analyses (n = 17,544 patients), 46 genetic variants were assessed, and 9 were associated with a response (Supplementary Table S7). The strongest associations were observed for SNPs in the C9orf72, IFNK, NLRP3, TNFα, and TNFα receptor genes (Figure 3). The minor alleles of C9orf72 (rs3849942) (three studies, n = 319 [47,48,49]), IFNK (rs7046653) (three studies, n = 319 [47,48,49]), and NLRP3 (rs4612666) (three studies, n = 2036 [50,51,52]) were associated with poor response to TNFi (OR: 0.50 [95% CI: 0.27–0.93], 0.60 [95% CI: 0.37–0.99], and 0.67 [95% CI: 0.48–0.93], respectively).

Three SNPs in the TNF-α gene (TNFα-238 (rs361525), TNFα-308 (rs1800629), and TNF-α-857 (rs1799724)) have been assessed in multiple studies with conflicting results (Supplementary Table S4). In the meta-analysis, only the minor allele of TNF-α-857 (rs1799724) was associated with a response to treatment with etanercept (OR: 3.05 [95% CI: 1.09–8.53]) based on two studies and 171 patients [29,53]. Of the TNF receptors, the minor allele of TNFRSF1B (rs1061622) was associated with poor response in RA (OR: 0.25 [95% CI: 0.11–0.57]) based on two studies (n = 143 patients) when only including infliximab [29,54].

Two genetic variants in the Fcγ receptor (FCGR2A (rs1801274) and FCGR3A (rs396991)) have been associated with response to treatment with TNFi in RA. In the meta-analysis, having the minor allele of FCGR3A (rs396991) was associated with poor response to treatment with TNFi (OR: 0.77 [95% CI: 0.59–0.99]) across eight studies (n = 1221 patients) [45,54,55,56,57,58,59,60], whereas no association was observed for FCGR2A (rs1801274) across five studies (n = 1228 patients) [57,58,61,62,63].

#### 3.3.2. Association between SNPs and Response to Rituximab in Rheumatoid Arthritis

In total, 12 studies including 1998 patients assessed the association between genetic variants and response to treatment of RA patients with rituximab. Of the assessed genetic variants, nine across eight genes were associated with response to rituximab (Supplementary Table S4). Of the genetic variants, three could be included in the meta-analysis, of which two were associated with a response to rituximab in RA. The minor allele of TNFSF13B (rs9514828) was associated with poor response (OR: 0.33 [95% CI: 0.13–0.82]) to rituximab across two studies (n = 267 patients [64,65]). The minor allele of FCGR3A (rs396991) was associated with a good response to rituximab (OR: 1.71 [95% CI: 1.16–2.51]) across five studies (n = 602 patients [66,67,68,69,70]).

#### 3.3.3. Association between SNPs and Response to Tocilizumab in Rheumatoid Arthritis

In total, ten studies including 2311 patients assessed the association between genetic variants and response to treatment tocilizumab in patients with RA. Of the assessed genetic variants, 14 across 12 genes were associated with response to tocilizumab. Four studies (n = 397 patients [68,71,72,73]) were included in the meta-analysis assessing five SNPs, two of which were associated with responses (Supplementary Table S4). The minor allele of IL-6R (rs12083537) (two studies, n = 229 patients [71,72]) was associated with poor response (OR: 0.47 [95% CI: 0.24–0.89] and the minor allele of IL-6R (rs2228145) (two studies, n = 156 patients [71,73]) was associated with good response to tocilizumab (OR: 10.71 [95% CI: 3.46–33.10]), Figure 3.

#### 3.3.4. Association between SNPs and Response to IL-1Ri in Rheumatoid Arthritis

One study including 80 patients assessed the association between six genetic variants and response to treatment of RA patients with anti-IL-R1 and found three SNPs (IL-1B (+3954) (rs1143634), IL-1A (+4845) (rs17561) and IL-1A (−889) (rs1800587)) across two genes were associated with response to anti-IL-R1 [74] (Supplementary Table S4).

#### 3.3.5. Association between SNPs and Response to Abatacept in Rheumatoid Arthritis

In total, three studies including 272 patients assessed the association between genetic variants and response to treatment with abatacept in RA patients. Of the six assessed genetic variants, three genetic variants across two genes were associated with response to abatacept (Supplementary Table S4). Only the SNP in FCGR3A (rs396991) could be included in the meta-analysis, showing no association with response to abatacept across two studies including 141 patients [75,76].

### 3.4. Association between SNPs and Response to Biologics in Inflammatory Bowel Diseases

In total, 46 studies including 15,006 patients assessed the association between genetic variants and response to biologics in patients with IBD. Of the genetic variants, 166 were associated with response to treatment with biologics among patients with IBD of which most were assessed for TNFi, and most were only assessed in one study (Supplementary Table S5).

#### 3.4.1. Association between SNPs and Response to TNFi in Inflammatory Bowel Diseases

In total, 46 studies including 14,896 patients assessed the association between genetic variants and response to treatment of IBD with TNFi. Of the genetic variants, 166 across 132 genes were associated with response to TNFi (Supplementary Table S5). Thirteen studies assessed SNPs for TNFi as a class, and 33 studies investigated the individual TNFi. The SNPs were most often assessed in the genes linked to TNF-α signaling or in mechanisms important for the IBD pathogenesis.

**Figure 4:**
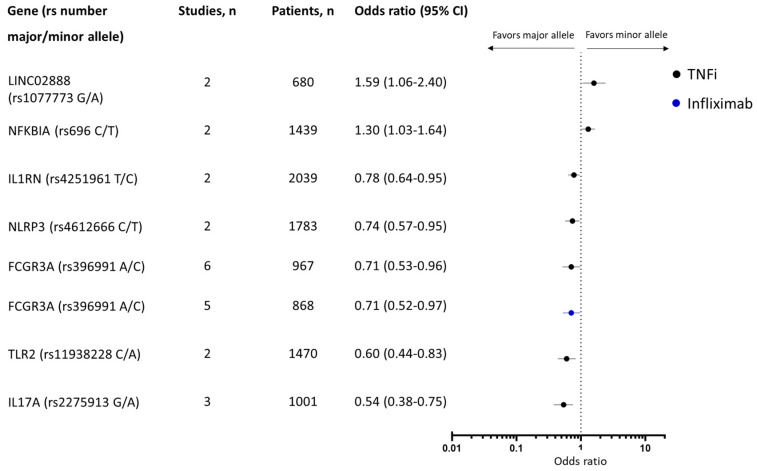
Genetic variants significantly associated with response to biologics among patients with inflammatory bowel diseases in the meta-analysis.

In the meta-analysis, 33 genetic variants were assessed, and 7 were associated with response (Figure 4). The minor alleles of LINC02888 (rs1077773) (two studies, n = 680 patients [77,78]) and NFKBIA (rs696) (two studies, n = 1778 patients [30,79]) were associated with good response to TNFi (OR: 1.59 [95% CI: 1.06–2.40] and OR: 1.30 [95% CI: 1.03–1.64]). The minor alleles of IL-1RN (rs4251961) (two studies, n = 2039 patients [30,79]), IL-17A (rs2275913) (three studies, n = 1001 patients [30,80,81]), TLR2 (rs11938228) (two studies, n = 1470 patients [30,79]), and NLRP3 (rs4612666) (two studies, n = 1783 patients [30,79]) were all associated with poor response (OR: 0.78 [95% CI: 0.64–0.95], OR: 0.54 [95% CI: 0.38–0.75]), OR: 0.60 [95% CI: 0.44–0.83], and 0.74 [95% CI: 0.57–0.95], respectively), Figure 4. None of the SNPs in the TNF-α gene (TNF-α-238 (rs361525), TNF-α-308 (rs1800629), and TNF-α-857 (rs1799724)) were significantly associated with response to TNFi in IBD. Likewise, none of the SNPs in the TNF receptors (TNFRSF1B (rs1061622), TNFRSF1A (rs767455), TNFRSF1A (rs4149570), and TNFRSF1B (rs3397)) were found associated with response to TNFi in IBD (Supplementary Table S5).

Two genetic variants in the Fcγ receptor (FCGR2A (rs1801274) and FCGR3A (rs396991)) have been associated with response to treatment with TNFi in IBD in several studies. In the meta-analysis, having the minor allele of FCGR3A (rs396991) was associated with poor response to treatment both with TNFi overall (OR: 0.71 [95% CI: 0.53–0.96], six studies and 967 patients [82,83,84,85,86,87]) and to infliximab alone (0.71 [95% CI: 0.52–0.97], five studies and 868 patients [83,84,85,86,87]).

#### 3.4.2. Association between SNPs and Response to IL-12/-23i in Inflammatory Bowel Diseases

One study including 110 patients assessed the association between 7 genetic variants and response to treatment of IBD with IL-12/-23i. Here, only PTPN2 (rs7234029) was associated with a non-response to IL-12/-23i [88] (Supplementary Table S5).

### 3.5. Association between SNPs and Response to Biologics for All Chronic Inflammatory Diseases Together

**Figure 5:**
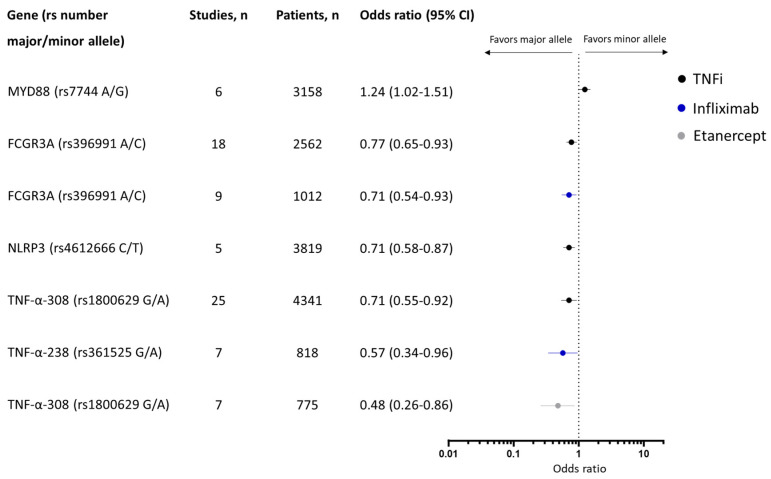
Genetic variants significantly associated with response to biologics among patients across all indications in the meta-analysis.

In total, 13 SNPs were included in the meta-analyses in ≥ two diseases for the same drug and/or drug class and were pooled across diseases (Table 1). Here, 5 SNPs were associated with response to TNFi (Figure 5). The minor allele of MYD [88] (rs7744) was associated with response to TNFi (OR: 1.24 [95% CI: 1.02–1.51]) across six studies including 3158 patients with psoriasis [24,32] or RA51 [89,90,91], and the minor allele of NLRP3 (rs4612666) was associated with poor response to TNFi (OR: 0.71 [95% CI: 0.58–0.87]) across five studies including 3819 patients with RA [50,51,52] or IBD [30,79]. In the TNFα gene, both TNF-α-238 (rs361525) and TNF-α-308 (rs1800629) were associated with response. The minor allele of TNF-α-308 (rs1800629) was associated with poor response both to TNFi overall (0.71 [95% CI: 0.55–0.92]) across 25 studies including 4341 patients with psoriasis [24,36,37,38], RA [29,62,92,93,94,95,96,97,98,99,100], or IBD [30,82,83,85,86,101,102,103,104] and response to etanercept (0.48 [95% CI: 0.26–0.86]) across seven studies including patients with psoriasis [36,38] and RA [29,93,95,96,98]. While no association was observed when TNFi were analyzed together, the minor allele of TNF-α-238 (rs361525) was associated with poor response to infliximab (OR: 0.57 [95% CI: 0.34–0.96]) across seven studies including 818 patients with psoriasis [29,37,97,98], or IBD [83,85,102]. Of genetic variants in the Fcγ receptor, only FCGR3A (rs396991) was associated with response to TNFi. The minor allele of FCGR3A (rs396991) was associated with poor response both to TNFi overall (OR: 0.77 [95% CI: 0.65–0.93]) across 18 studies including 2562 patients with psoriasis [105,106,107], PsA [46], RA [45,54,55,56,57,58,59,60], or IBD [82,83,84,85,86,87] and infliximab (OR: 0.71 [95% CI: 0.54–0.93]) across studies including 1012 patients with R [45,54,58,59] or IBD [83,84,85,86,87].

## 4. Discussion

In this systematic review and meta-analysis, we identified and systematically reported all SNPs outside the HLA region that were associated with a response to treatment with biologics for patients with psoriasis, PsA, RA, and IBD. In total, 126 SNPs have been associated with response in psoriasis, 9 in PsA, 232 in RA, and 166 in IBD. In the meta-analysis, 5 SNPS showed significant association with a response to the CIDs together, 15 in psoriasis, none in PsA, 9 in RA, and 7 in IBD.

Identification of genetic markers of treatment response is of great importance as a considerable proportion of patients treated with biologics do not respond to the given biologic. As shown in this systematic review and meta-analysis, multiple studies have assessed genetic markers and found them associated with response. Across all CIDs, genetic variants in TNF-α and Fc-γ receptor genes in relation to response to TNFi were the most heavily investigated.

A previous meta-analysis found no association between the minor allele of TNF-α-308 (rs1800629) and response to TNFi among patients with psoriasis or IBD108, but did find an association for patients with RA [121,122]. Interestingly, we found the minor allele of TNF-α-308 (rs1800629) to be associated with a poor response to TNFi when assessing all CIDs together, but only for psoriasis when the diseases were examined individually. We did see a similar direction of response across the diseases, and the lack of association might be due to differences in design and the number of patients. The minor allele of TNF-α-238 (rs361525) was not associated with response to TNFi for any of the diseases when examining TNFi together. However, when evaluating etanercept alone, the minor allele was associated with poor response to TNFi in psoriasis and infliximab alone when all diseases were pooled. This could be due to differences in the mode of action of the individual TNFi, as the association with the response to adalimumab appeared to be in the opposite direction than the other TNFis although this was not significant. In agreement with Antonatos et al. [121], the current meta-analysis only found an association between the minor allele of TNF-α-857 (rs1799724) and the poor response to TNFi in patients with psoriasis. The persistent association between genetic variants in the TNF-α genes and response to TNFi underlines the importance of these genes. The genetic variants have been shown to alter the expression and/or levels of TNF-α, which is a plausible mechanism for association [123]. However, the conflicting results and potential differences in the drugs warrant further studies differentiating the individual drugs.

The association between genetic variants in the Fcγ-receptors (FCGR3A (rs396991) and FCGR2A (rs1801274)) and response to biologics have been assessed in several studies. The FcγR2A- and FcγR3A receptors play a central role in antibody-dependent immune response. FCGR3A (rs396991) has been associated with the development of RA [124] and FCGR2A (rs1801274) with UC [125]. In the meta-analysis, we found the minor allele of FCGR3A (rs396991) to be associated with poor response to TNFi and infliximab alone when pooling all CIDs together; similarly, this was observed for RA and IBD alone but not for psoriasis. For rituximab, the opposite association was observed, with the minor allele being associated with a good response to treatment in patients with RA. Interestingly, the minor allele of FCGR3A (rs396991) has been shown to lead to higher surface expression of FCGR3A [126] and also has a higher affinity to IgG than the major allele [127]. For TNFi, the lower response rate has been suggested to be a result of higher FcγR-mediated drug clearance resulting in lower drug levels and, therefore, a worse response [58], while higher levels of FcγRIIIa/CD16 expression leading to higher B-cell depletion have been suggested to be the reason for better response to rituximab in patients having the minor allele [70,128]. These results suggest FCGR3A (rs396991) to be a potential variant for choice of drug in patients with RA. However, further investigations into the association between SNPs in the Fcγ receptors, drug levels, and response to biologics are warranted.

Some limitations should be considered when interpreting the results of the meta-analyses. First, the included studies were heterogeneous with heterogeneous populations, response criteria, and sample size, which complicates the meta-analysis and interpretation of the results. Furthermore, most studies assessed TNFi as a group, and differences in structure, affinity, and specific target of the individual target might lead to different effects of the SNPs. Few studies have assessed drugs other than TNFi, and to be able to use genetic variants to stratify patients, the association between SNPs and other treatments needs to be conducted. Other factors, including biomarkers like serostatus for RA [129], and clinical factors like smoking and body weight [130,131,132,133,134], can influence response and might dilute the result of the meta-analysis. However, these should not be considered classical confounders as few of the SNPs are believed to be associated with these. Lastly, only individual SNPs could be included, and the inclusion of pathway analyses might have revealed other genetic variants associated with response. Similarly, the identification of underlying biological mechanisms may improve the predictive potential. On the other hand, the identification of markers across several diseases, including heterogeneous studies, underlines the robustness of the observed associations.

Taken together, numerous SNPs have been associated with a response to treatment with biologics in psoriasis, PsA, RA, and IBD. The current systematic review provides an overview of these SNPs. However, most of the studied SNPs have only been assessed once or twice, and in the meta-analysis, only a fraction of the SNPs was significant and primarily in treatment with TNFi. This indicates that several of the SNPs that were found to be associated in individual studies could be due to chance findings and require further investigation and validation in other cohorts as well as assessed for biologics with other targets. Furthermore, the results suggest that none of the assessed SNPs alone can predict whether a patient will respond or not to a given biologic, and collaborations working toward polygenic risk gene scores should be established.

In conclusion, we present a systematic overview of all SNPs that have been associated with response to biologics in the treatment of psoriasis, PsA, RA, and IBD. Genetic variants in TNFα, NLRP3, MYD [88], and FcγR genes are associated with response to TNFi when assessing several inflammatory diseases together. For the individual diseases, 126 genetic variants were found to be associated with a response to biologics in psoriasis, 9 in PsA, 232 in RA, and 166 in IBD. In the meta-analysis, 15 genetic variants showed significant association with a response in psoriasis, 12 in RA, 7 in IBD, and none in PsA. None of the individual SNPs alone can predict whether a patient will respond to a given biologic. More studies are needed to test and validate these genetic variants in the search for predictive biomarkers.

## Figures and Tables

**Table 1:** Pooled odds ratio using random effects meta-analysis for all diseases combined.

Gene (rs Number)	Drug	Studies (n)	Patients (n)	Odds Ratio (95% CI) #	I2 (95% CI)
**FCGR2A** (rs1801274)	TNFi-combined	10	Total: 1675 PsO: 202 [61] + 302 [57] + 291 [62] + 85 [58] + 348 [63] = 1228 RA: 100 [105] + 70 [106] + 144 [32] + 30 [107] = 344 PsA: 103 [46]	0.88 (0.54–1.45)	68.1% (24.9% to 81.9%)
***FCGR2A *** *(rs1801274)*	*Etanercept*	*4*	*Total: 264* *PsO: 55* [105] *+ 30* [107] *= 85* *RA: 124* [63] *PsA: 55* [46]	*1.12 (0.57–2.24)*	*6% (0% to 69.8%)*
**FCGR3A ** (rs396991)	TNFi-combined	18	Total: 2562 PsO: 100 [105] + 56 [106] + 115 [107] = 271 RA: 301 [57] + 377 [55] + 37 [45] + 77 [58] + 282 [56] + 78 [54] + 36 [59] + 33 [60] = 1221 IBD: 120 [82] + 121 [83] + 200 [84] + 106 [85] + 76 [86] + +344 [87] = 967 PsA: 103 [46]	0.77 (0.65–0.93) *	46.7% (0% to 68.1%)
***FCGR3A *** *(rs396991)*	*Etanercept*	*4*	*Total: 264* *PsO: 55* [105] *+ 30* [107] *= 85* *RA: 124* [63] *PsA: 55* [46]	*1.04 (0.15–7.35)*	*79.7% (0% to 91.7%)*
***FCGR3A *** *(rs396991)*	*Infliximab*	*9*	*Total: 1012* *RA: 37* [45] *+ 78* [54] *+ 77* [58] *+ 29* [59] *= 144* *IBD: 121* [83] *+ 200* [84] *+ 106* [85] *+ 76* [86] *+ 344* [87] *= 868*	0.71 (0.54–0.93) *	*38% (0% to 70.2%)*
**GBP6** (rs928655)	TNFi-combined	5	Total: 531 PsO: 144 [32] + 68 [35] = 212 RA: 135 [48] + 89 [49] + 95 [47] = 319	2.06 (0.83–5.10)	74.2% (1.9% to 87.7%)
**IL-17A** (rs2275913)	TNFi-overall	6	Total: 1525 PsO: 143 [108] + 132 [44] + 249 [24] = 524 IBD: 103 [80] + 209 [81] + 689 [30] = 1001	0.79 (0.48–1.31)	61.8% (0% to 82.3%)
**MYD88** (rs7744)	TNFi-overall	6	Total: 3168 PsO: 144 [32] + 249 [24] = 393 RA: 902 [89] + 183 [90] + 689 [91] + 991 [51] = 2765	1.24 (1.02–1.51) *	31.1% (0% to 72%)
**NFKBIA** (rs696)	TNFi-combined	4	Total: 2121 PsO: 96 [109] + 247 [24] = 343 IBD: 725 [30] + 1053 [79] =1778	1.31 (0.95–1.80)	35.8 (0% to 78.1%)
**NLRP3** (rs4612666)	TNFi-overall	5	Total: 3819 RA: 516 [50] + 988 [51] + 532 [52] = 2036 IBD: 1053 [79] + 730 [30] = 1783	0.71 (0.58–0.87) *	0% (0% to 64.1%)
**TNF-α-238 ** (rs361525)	TNFi-combined	14	Total: 2989 PsO: 102 [37] + 97 [36] + 249 [24] = 448 RA: 70 [53] + 360 [99] + 113 [97] + 476 [98] + 190 [29] = 1209 IBD: 222 [102] + 34 [103] + 729 [30] + 120 [82] + 121 [83] + 106 [85] = 1332	0.77 (0.52–1.13)	40.1% (0% to 66.9%)
***TNF-*α-*238 *** *(rs361525)*	*Adalimumab*	*4*	*Total: 516* *PsO: 28* [37] *RA: 59* [29] *+ 360* [99] *+ 69* [98] *= 488*	*1.61 (0.78– 3.33)*	*0% (0% to 67.9%)*
***TNF-*α-*238 *** *(rs361525)*	*Etanercept*	*5*	*Total:521* *RA: 70* [53] *+ 198* [98] *+ 102* [29] *= 370* *PsO: 97* [36] *+ 54* [37] *= 151*	*0.44 (0.18–1.02)*	*40.3% (0% to 76.9%)*
***TNF-*α-*238 *** *(rs361525)*	*Infliximab*	*7*	*Total: 818* *PsO: 27* [37] *RA:113* [97] *+ 209* [98] *+ 20* [29] *= 342* *IBD: 222* [102] *+ 121* [83] *+ 106* [85] *= 449*	0.57 (0.34–0.96) *	*0% (0% to 58.5%)*
**TNF-α-308** (rs1800629)	TNFi-combined	25	*Total: 4341* *PsO: 102* [37] + 97 [36] + 249 [24] + 100 [38] = 548 *RA:* 187 [29] + 73 [93] + 78 [94] + 123 [95] + 260 [62] + 53 [96] + 86 [96] + 113 [97] + 474 [98] + 369 [99] + 198 [100] + 100 [92] = 2114 *IBD:* 214 [101] + 222 [102] + 34 [103] + 119 [82] + 121 [83] + 734 [30] + 107 [85] + 76 [86] + 52 [104] = 1679	0.71 (0.55–0.92) *	53% (17.9% to 69.2%)
***TNF-*α-*308*** *(rs1800629)*	*Etanercept*	*7*	*Total: 775* *RA: 73* [93] *+ 123* [95] *+ 86* [96] *+ 197* [98] *+ 99* [29] *=578* *PsO: 97* [36] *+ 100* [38] =197	0.48 (0.26–0.86) *	*57.3% (0% to 79.6%)*
***TNF-*α-*308*** *(rs1800629)*	*Infliximab*	*11*	*Total: 1195* *RA: 53* [110] *+ 113* [97] *+ 198* [100] *+ 19* [29] *+ 20* [111] *= 403* *IBD: 222* [102] *+ 214* [101] *+ 121* [83] *+ 107* [85] *+ 76* [86] *+ 52* [104] *= 792*	*0.84 (0.58–1.21)*	*17.4% (0% to 59.3%)*
**TNF-α-857** (rs1799724)	TNFi-combined	12	Total: 1218 PsO: 80 [39] + 102 [37] + 97 [36] + 49 [40] = 328 RA: 190 [29] + 357 [99] + 70 [53] + 100 [92] = 717 IBD: *222* [102] *+ 121* [83] *+ 106* [85] *+ 52* [104] *= 501*	0.71 (0.42–1.19)	67.1% (29.1% to 80.6%)
***TNF-*α-*857*** *(rs1799724)*	*Etanercept*	*5*	*Total: 366* *PsO: 97* [36] *+ 54* [37] *+ 44* [39] *= 195* *RA: 101* [29] *+ 70* [53] = 171	*1.15 (0.36–3.71)*	*73.1% (0% to 87.3%)*
***TNF-*α-*857*** *(rs1799724)*	*Infliximab*	*6*	*Total: 543* *PsO: 27* [37] *RA: 15* [29] *IBD: 222* [102] *+ 121* [83] *+ 106* [85] *+ 52* [104] *= 501*	*0.40 (0.15–1.04)*	*55.7% (0% to 80.2%)*
**TNFR1A** (rs767455)	TNFi-combined	6	Total: 819 RA: 58 [112] + 89 [113] + 187 [29] = 334 IBD: *81* [114] *+ 283* [115] *+ 121* [83] *=485*	1.06 (0.76–1.46)	0% (0% to 61%)
***TNFR1A *** *(rs767455)*	*Infliximab*	*6*	*Total: 653* *RA: 58* [112] *+ 89* [113] *+ 21* [29] *= 168* *IBD: 81* [114] *+ 283* [115] *+ 121* [83] *= 485*	*1.00 (0.69–1.46)*	*0% (0% to 61%)*
**TNFRSF1A** (rs4149570)	TNFi-combined	5	Total: 2260 PsO: 144 [32] + 249 [24] = 393 IBD: 80 [114] + 718 [30] + 1069 [79] = 1867	0.97 (0.59–1.60)	65% (0% to 84.5%)
**TNFRSF1B ** (rs1061622)	TNFi-combined	17	Total: 2004 PsO: 80 [39] + 90 [42] + 144 [32] + 95 [41] + 49 [40] + 100 [38] = 558 RA: 212 [116] + 122 [54] + 190 [29] + 15 [117] + 456 [118] + 100 [92] = 1095 IBD: 90 [119] + 80 [114] + 67 [120] + 293 [115] + 121 [83] = 651	0.77 (0.56–1.08)	62.5% (28.4% to 76.5%)
***TNFRSF1B *** *(rs1061622)*	*Infliximab*	*7*	*Total: 794* *RA: 122* [54] *+ 21* [29] *= 143* *IBD: 90* [119] *+ 80* [114] *+ 67* [120] *+ 293* [115] *+ 121* [83] *= 651*	*1.06 (0.62–1.81)*	*62.8% (0% to 81.7%)*

TNFi combined: includes studies assessing TNFi overall but also individual drugs. TNFi overall: includes only studies assessing TNFi overall as a drug class. * *p* < 0.05. # odds ratio (OR) of response comparing minor allele with major allele, OR > 1 favors major allele, OR < 1 favors minor allele.

## Supplementary Materials

The following supporting information can be downloaded at: https://www.mdpi.com/article/10.3390/ijms25115793/s1.

## Abbreviations

ACR	American College of Rheumatology
bDMARDs	biologic disease-modifying anti-rheumatic drugs
CAI	clinical activity index
CD	Crohn’s disease
CD-20	cluster of differentiate-20
CDAI	Crohn’s Disease Activity Index
CRP	C-reactive protein
DMARDs	disease-modifying anti-rheumatic drugs
DAS28	disease activity score 28 joints
EULAR	European League Against Rheumatism
HBI	Harvey–Bradshaw index
HLA	human leukocyte antigen
IBD	inflammatory bowel disease
IBDQ	Inflammatory Bowel Disease Questionnaire
IFN-γ	Interferon-γ
IL-17	Interleukin-17
IL12/23i	Interleukin-12/23-inhibitor
NCBI	National Center of Biotechnology Information
NIH	National Institute of Health
OR (95%) CI	odds ratio (95% Confidence interval)
PASI	psoriasis area and severity index
PRISMA	Preferred Reporting Items for systematic Reviews and Meta-analyses
PsA	psoriatic arthritis
PUCAI	Pediatric Ulcerative Colitis Activity Index
RA	rheumatoid arthritis
SNP	single nucleotide polymorphism
Th-cell	T-helper-cell
TNF-α	tumor necrosis factor-α
TNFi	tumor necrosis factor-inhibitor
UC	ulcerative colitis

## References

[1] Parisi R., Symmons D.P.M., Griffiths C.E., Ashcroft D.M., on behalf of the Identification and Management of Psoriasis and Associated ComorbidiTy (IMPACT) Project Team (2013). Global Epidemiology of Psoriasis: A Systematic Review of Incidence and Prevalence. J. Investig. Dermatol..

[2] Scotti L., Franchi M., Marchesoni A., Corrao G. (2018). Prevalence and incidence of psoriatic arthritis: A systematic review and meta-analysis. Semin. Arthritis Rheum..

[3] Lees C.W., Barrett J.C., Parkes M., Satsangi J. (2011). New IBD genetics: Common pathways with other diseases. Gut.

[4] Li Y., Begovich A.B. (2009). Unraveling the genetics of complex diseases: Susceptibility genes for rheumatoid arthritis and psoriasis. Semin. Immunol..

[5] Rendon A., Schäkel K. (2019). Psoriasis Pathogenesis and Treatment. Int. J. Mol. Sci..

[6] McInnes I.B.P., Schett G.P. (2017). Pathogenetic insights from the treatment of rheumatoid arthritis. Lancet.

[7] Zhang Y.-Z., Li Y.-Y. (2014). Inflammatory bowel disease: Pathogenesis. World J. Gastroenterol..

[8] Gálvez J. (2014). Role of Th17 Cells in the Pathogenesis of Human IBD. ISRN Inflamm..

[9] Kany S., Vollrath J.T., Relja B. (2019). Cytokines in Inflammatory Disease. Int. J. Mol. Sci..

[10] Langer V., Vivi E., Regensburger D., Winkler T.H., Waldner M.J., Rath T., Schmid B., Skottke L., Lee S., Jeon N.L. (2019). IFN-γ drives inflammatory bowel disease pathogenesis through VE-cadherin–directed vascular barrier disruption. J. Clin. Investig..

[11] Schurich A., Raine C., Morris V., Ciurtin C. (2018). The role of IL-12/23 in T cell-related chronic inflammation: Implications of immunodeficiency and therapeutic blockade. Rheumatology.

[12] Li P., Zheng Y., Chen X. (2017). Drugs for Autoimmune Inflammatory Diseases: From Small Molecule Compounds to Anti-TNF Biologics. Front. Pharmacol..

[13] Nielsen O.H., Ainsworth M.A., Nielsen O.H., Ainsworth M.A. (2013). Tumor Necrosis Factor Inhibitors for Inflammatory Bowel Disease. New Engl. J. Med..

[14] Smolen J.S., Aletaha D., Koeller M., Weisman M.H., Emery P. (2007). New therapies for treatment of rheumatoid arthritis. Lancet.

[15] Honap S., Meade S., Ibraheim H., Irving P.M., Jones M.P., Samaan M.A. (2021). Effectiveness and Safety of Ustekinumab in Inflammatory Bowel Disease: A Systematic Review and Meta-Analysis. Dig. Dis. Sci..

[16] Thatiparthi A., Martin A., Liu J., Egeberg A., Wu J.J. (2021). Biologic Treatment Algorithms for Moderate-to-Severe Psoriasis with Comorbid Conditions and Special Populations: A Review. Am. J. Clin. Dermatol..

[17] Geale K., Lindberg I., Paulsson E.C., Wennerström E.C.M., Tjärnlund A., Noel W., Enkusson D., Theander E. (2020). Persistence of biologic treatments in psoriatic arthritis: A population-based study in Sweden. Rheumatol. Adv. Pract..

[18] Nast A., Jacobs A., Rosumeck S., Werner R.N. (2015). Efficacy and Safety of Systemic Long-Term Treatments for Moderate-to-Severe Psoriasis: A Systematic Review and Meta-Analysis. J. Investig. Dermatol..

[19] Grant R.K., Jones G.-R., Plevris N., Lynch R.W., Jenkinson P.W., Lees C.W., A Manship T., Jagger F.A.M., Brindle W.M., Shivakumar M. (2020). The ACE (Albumin, CRP and Endoscopy) Index in Acute Colitis: A Simple Clinical Index on Admission that Predicts Outcome in Patients With Acute Ulcerative Colitis. Inflamm. Bowel Dis..

[20] Baert F., Casteele N.V., Tops S., Noman M., Van Assche G., Rutgeerts P., Gils A., Vermeire S., Ferrante M. (2014). Prior response to infliximab and early serum drug concentrations predict effects of adalimumab in ulcerative colitis. Aliment. Pharmacol. Ther..

[21] Enevold C., Loft N., Bregnhøj A., Zachariae C., Iversen L., Skov L., Nielsen C.H. (2022). Circulating Brodalumab Levels and Therapy Outcomes in Patients With Psoriasis Treated With Brodalumab. JAMA Dermatol..

[22] Loft N., Bregnhoj A., Fage S., Nielsen C.H., Enevold C., Zachariae C., Iversen L., Skov L. (2021). Effectiveness of brodalumab after previous treatment failure of interleukin-17A inhibitors in patients with psoriasis. Dermatol. Ther..

[23] Andersen C.S.B., Kvist-Hansen A., Siewertsen M., Enevold C., Hansen P.R., Kaur-Knudsen D., Zachariae C., Nielsen C.H., Loft N., Skov L. (2023). Blood Cell Biomarkers of Inflammation and Cytokine Levels as Predictors of Response to Biologics in Patients with Psoriasis. Int. J. Mol. Sci..

[24] Loft N.D., Skov L., Iversen L., Gniadecki R., Dam T.N., Brandslund I., Hoffmann H.J., Andersen M.R., Dessau R.B., Bergmann A.C. (2017). Associations between functional polymorphisms and response to biological treatment in Danish patients with psoriasis. Pharmacogenomics J..

[25] Conigliaro P., Ciccacci C., Politi C., Triggianese P., Rufini S., Kroegler B., Perricone C., Latini A., Novelli G., Borgiani P. (2017). Polymorphisms in STAT4, PTPN2, PSORS1C1 and TRAF3IP2 Genes Are Associated with the Response to TNF Inhibitors in Patients with Rheumatoid Arthritis. PLoS ONE.

[26] Maldonado-Montoro M., Cañadas-Garre M., González-Utrilla A., Plaza-Plaza J.C., Calleja-Hernández M. (2016). Genetic and clinical biomarkers of tocilizumab response in patients with rheumatoid arthritis. Pharmacol. Res..

[27] Thomas D. (2014). Association of rs1568885, rs1813443 and rs4411591 polymorphisms with anti-TNF medication response in Greek patients with Crohn’s disease. World J. Gastroenterol..

[28] Julià A., Fernandez-Nebro A., Blanco F., Ortiz A., Cañete J.D., Maymó J., Alperi-López M., Fernández-Gutierrez B., Olivè A., Corominas H. (2015). A genome-wide association study identifies a new locus associated with the response to anti-TNF therapy in rheumatoid arthritis. Pharmacogenomics J..

[29] Swierkot J., Bogunia-Kubik K., Nowak B., Bialowas K., Korman L., Gebura K., Kolossa K., Jeka S., Wiland P. (2015). Analysis of associations between polymorphisms within genes coding for tumour necrosis factor (TNF)-alpha and TNF receptors and responsiveness to TNF-alpha blockers in patients with rheumatoid arthritis. Jt. Bone Spine.

[30] Bank S., Andersen P.S., Burisch J., Pedersen N., Roug S., Galsgaard J., Turino S.Y., Brodersen J.B., Rashid S., Rasmussen B.K. (2014). Associations between functional polymorphisms in the NFκB signaling pathway and response to anti-TNF treatment in Danish patients with inflammatory bowel disease. Pharmacogenomics J..

[31] Ovejero-Benito M.C., Munoz-Aceituno E., Sabador D., Almoguera B., Prieto-Perez R., Hakonarson H., Coto-Segura P., Carretero G., Reolid A., Llamas-Velasco M. (2020). Genome-wide association analysis of psoriasis patients treated with anti-TNF drugs. Exp. Dermatol..

[32] Prieto-Pérez R., Solano-López G., Cabaleiro T., Román M., Ochoa D., Talegón M., Baniandrés O., López-Estebaranz J.L., de la Cueva P., Daudén E. (2016). New polymorphisms associated with response to anti-TNF drugs in patients with moderate-to-severe plaque psoriasis. Pharmacogenomics J..

[33] Siewertsen M., Al-Sofi R., Dridi H., Ajenthen G.D., Zachariae C., Skov L., Loft N. (2023). Association between HLA-Cw6 and response to treatment with biologics in patients with psoriasis: A systematic review and meta-analysis. J. Eur. Acad. Dermatol. Venereol..

[34] Ouzzani M., Hammady H., Fedorowicz Z., Elmagarmid A. (2016). Rayyan—A web and mobile app for systematic reviews. Syst. Rev..

[35] Ovejero-Benito M.C., Prieto-Pérez R., Llamas-Velasco M., Belmonte C., Cabaleiro T., Román M., Ochoa D., Talegón M., Saiz-Rodríguez M., Daudén E. (2017). Polymorphisms associated with etanercept response in moderate-to-severe plaque psoriasis. Pharmacogenomics.

[36] De Simone C., Farina M., Maiorino A., Fanali C., Perino F., Flamini A., Caldarola G., Sgambato A. (2015). TNF-alpha gene polymorphisms can help to predict response to etanercept in psoriatic patients. J. Eur. Acad. Dermatol. Venereol..

[37] Gallo E., Cabaleiro T., Román M., Solano-López G., Abad-Santos F., García-Díez A., Daudén E. (2013). The relationship between tumour necrosis factor (TNF)-α promoter andIL12B/IL-23Rgenes polymorphisms and the efficacy of anti-TNF-α therapy in psoriasis: A case-control study. Br. J. Dermatol..

[38] Hassan Hadi A.M., Abbas A.A.H., Abdulamir A.S., Fadheel B.M. (2020). The effect of TnFaip3 gene polymorphism on disease susceptibility and response of etanercept in psoriatic patients. Eur. J. Mol. Clin. Med..

[39] Vasilopoulos Y., Manolika M., Zafiriou E., Sarafidou T., Bagiatis V., Krüger-Krasagaki S., Tosca A., Patsatsi A., Sotiriadis D., Mamuris Z. (2012). Pharmacogenetic Analysis of TNF, TNFRSF1A, and TNFRSF1B Gene Polymorphisms and Prediction of Response to Anti-TNF Therapy in Psoriasis Patients in the Greek Population. Mol. Diagn. Ther..

[40] Ito M., Hirota T., Momose M., Ito T., Umezawa Y., Fukuchi O., Asahina A., Nakagawa H., Tamari M., Saeki H. (2019). Lack of association of TNFA, TNFRSF1B and TNFAIP3 gene polymorphisms with response to anti-tumor necrosis factor therapy in Japanese patients with psoriasis. J. Dermatol..

[41] Ovejero-Benito M.C., Prieto-Pérez R., Llamas-Velasco M., Muñoz-Aceituno E., Reolid A., Saiz-Rodríguez M., Belmonte C., Román M., Ochoa D., Talegón M. (2018). Polymorphisms associated with adalimumab and infliximab response in moderate-to-severe plaque psoriasis. Pharmacogenomics.

[42] González-Lara L., Batalla A., Coto E., Gómez J., Eiris N., Santos-Juanes J., Queiro R., Coto-Segura P. (2014). The TNFRSF1B rs1061622 polymorphism (p.M196R) is associated with biological drug outcome in Psoriasis patients. Arch. Dermatol. Res..

[43] Morelli M., Galluzzo M., Madonna S., Scarponi C., Scaglione G.L., Galluccio T., Andreani M., Pallotta S., Girolomoni G., Bianchi L. (2020). HLA-Cw6 and other HLA-C alleles, as well as MICB-DT, DDX58, and TYK2 genetic variants associate with optimal response to anti-IL-17A treatment in patients with psoriasis. Expert Opin. Biol. Ther..

[44] van Vugt L., Reek J.v.D., Meulewaeter E., Hakobjan M., Heddes N., Traks T., Kingo K., Galluzzo M., Talamonti M., Lambert J. (2019). Response to IL-17A inhibitors secukinumab and ixekizumab cannot be explained by genetic variation in the protein-coding and untranslated regions of the IL-17A gene: Results from a multicentre study of four European psoriasis cohorts. J. Eur. Acad. Dermatol. Venereol..

[45] Morales-Lara M., Conesa-Zamora P., Simón G., Pedrero F., Santaclara V., Perez-Guillermo M., Soriano-Navarro E. (2010). Association between theFCGR3AV158F polymorphism and the clinical response to infliximab in rheumatoid arthritis and spondyloarthritis patients. Scand. J. Rheumatol..

[46] Ramírez J., Fernández-Sueiro J.L., López-Mejías R., Montilla C., Arias M., Moll C., Alsina M., Sanmarti R., Lozano F., Cañete J.D. (2012). FCGR2A/CD32AandFCGR3A/CD16AVariants and EULAR Response to Tumor Necrosis Factor-α Blockers in Psoriatic Arthritis: A Longitudinal Study with 6 Months of Followup. J. Rheumatol..

[47] Suarez-Gestal M., Perez-Pampin E., Calaza M., Gomez-Reino J.J., Gonzalez A. (2010). Lack of replication of genetic predictors for the rheumatoid arthritis response to anti-TNF treatments: A prospective case-only study. Arthritis Res. Ther..

[48] Krintel S.B., Palermo G., Johansen J.S., Germer S., Essioux L., Benayed R., Badi L., Østergaard M., Hetland M.L. (2012). Investigation of single nucleotide polymorphisms and biological pathways associated with response to TNFα inhibitors in patients with rheumatoid arthritis. Pharmacogenetics Genom..

[49] Liu C., Batliwalla F., Li W., Lee A., Roubenoff R., Beckman E., Khalili H., Damle A., Kern M., Furie R. (2008). Genome-Wide Association Scan Identifies Candidate Polymorphisms Associated with Differential Response to Anti-TNF Treatment in Rheumatoid Arthritis. Mol. Med..

[50] Sode J., Vogel U., Bank S., Andersen P.S., Thomsen M.K., Hetland M.L., Locht H., Heegaard N.H.H., Andersen V. (2014). Anti-TNF Treatment Response in Rheumatoid Arthritis Patients Is Associated with Genetic Variation in the NLRP3-Inflammasome. PLoS ONE.

[51] Sode J., Vogel U., Bank S., Andersen P.S., Hetland M.L., Locht H., Heegaard N.H.H., Andersen V. (2016). Confirmation of an IRAK3 polymorphism as a genetic marker predicting response to anti-TNF treatment in rheumatoid arthritis. Pharmacogenomics J..

[52] Lopez-Rodriguez R., Perez-Pampin E., Marquez A., Blanco F.J., Joven B., Carreira P., Ferrer M.A., Caliz R., Valor L., Narvaez J. (2018). Validation study of genetic biomarkers of response to TNF inhibitors in rheumatoid arthritis. PLoS ONE.

[53] Kang C.P., Lee K.W., Yoo D.H., Bae S.C. (2005). The influence of a polymorphism at position -857 of the tumour necrosis factor gene on clinical response to etanercept therapy in rheumatoid arthritis. Rheumatology.

[54] Rooryck C., Barnetche T., Richez C., Laleye A., Arveiler B., Schaeverbeke T. (2008). Influence of FCGR3A-V212F and TNFRSF1B-M196R genotypes in patients with rheumatoid arthritis treated with infliximab therapy. Clin. Exp. Rheumatol..

[55] Montes A., Perez-Pampin E., Joven B., Carreira P., Fernandez-Nebro A., del Carmen Ordonez M., Navarro-Sarabia F., Moreira V., Vasilopoulos Y., Sarafidou T. (2015). FCGR polymorphisms in the treatment of rheumatoid arthritis with Fc-containing TNF inhibitors. Pharmacogenomics..

[56] Kastbom A., Bratt J., Ernestam S., Lampa J., Padyukov L., Söderkvist P., Skogh T. (2007). Fcγ receptor type IIIA genotype and response to tumor necrosis factor α–blocking agents in patients with rheumatoid arthritis. Arthritis Rheum..

[57] Dávila-Fajardo C.L., Van Der Straaten T., Baak-Pablo R., Medarde Caballero C., Cabeza Barrera J., Huizinga T.W., Guchelaar H.-J., Swen J.J. (2015). FcGR genetic polymorphisms and the response to adalimumab in patients with rheumatoid arthritis. Pharmacogenomics..

[58] Canete J.D., Suarez B., Hernandez M.V., Sanmarti R., Rego I., Celis R., Moll C., A Pinto J., Blanco F.J., Lozano F. (2009). Influence of variants of Fcγ receptors IIA and IIIA on the American College of Rheumatology and European League Against Rheumatism responses to anti-tumour necrosis factor α therapy in rheumatoid arthritis. Ann. Rheum. Dis..

[59] Tsukahara S., Ikari K., Sato E., Yamanaka H., Hara M., Tomatsu T., Momohara S., Kamatani N., Katsunori Ikari K., Institute of Rheumatology, Tokyo Women’s Medical University (2008). A polymorphism in the gene encoding the Fc [GAMMA] IIIA receptor is a possible genetic marker to predict the primary response to infliximab in Japanese patients with rheumatoid arthritis. Ann Rheum Dis..

[60] Tutuncu Z., Kavanaugh A., Zvaifler N., Corr M., Deutsch R., Boyle D. (2005). Fcγ receptor type IIIA polymorphisms influence treatment outcomes in patients with inflammatory arthritis treated with tumor necrosis factor α–blocking agents. Arthritis Rheum..

[61] Montes A., Perez-Pampin E., Narváez J., Cañete J.D., Navarro-Sarabia F., Moreira V., Fernández-Nebro A., Ordóñez M.d.C., de la Serna A.R., Magallares B. (2014). Association of FCGR2A with the response to infliximab treatment of patients with rheumatoid arthritis. Pharmacogenetics Genom..

[62] Eektimmerman F., Swen J.J., Böhringer S., Huizinga T.W., Kooloos W.M., Allaart C.F., Guchelaar H.-J., I Danila M., Hughes L.B., Bridges S.L. (2017). Pathway analysis to identify genetic variants associated with efficacy of adalimumab in rheumatoid arthritis. Pharmacogenomics.

[63] Avila-Pedretti G., Tornero J., Fernández-Nebro A., Blanco F., González-Alvaro I., Cañete J.D., Maymó J., Alperiz M., Fernández-Gutiérrez B., Olivé A. (2015). Variation at FCGR2A and Functionally Related Genes Is Associated with the Response to Anti-TNF Therapy in Rheumatoid Arthritis. PLoS ONE.

[64] Ruyssen-Witrand A., Rouanet S., Combe B., Dougados M., Le Loët X., Sibilia J., Tebib J., Mariette X., Constantin A. (2012). Association between -871C>T promoter polymorphism in the B-cell activating factor gene and the response to rituximab in rheumatoid arthritis patients. Rheumatology.

[65] Fabris M., Quartuccio L., Vital E., Pontarini E., Salvin S., Fabro C., Zabotti A., Benucci M., Manfredi M., Ravagnani V. (2012). The TTTT B lymphocyte stimulator promoter haplotype is associated with good response to rituximab therapy in seropositive rheumatoid arthritis resistant to tumor necrosis factor blockers. Arthritis Rheum..

[66] Kastbom A., Cöster L., Ärlestig L., Chatzidionysiou A., van Vollenhoven R.F., Padyukov L., Rantapää-Dahlqvist S., Saevarsdottir S. (2012). Influence ofFCGR3Agenotype on the therapeutic response to rituximab in rheumatoid arthritis: An observational cohort study. BMJ Open.

[67] Pál I., Szamosi S., Hodosi K., Szekanecz Z., Váróczy L. (2017). Effect of Fcγ-receptor 3a (FCGR3A) gene polymorphisms on rituximab therapy in Hungarian patients with rheumatoid arthritis. RMD Open.

[68] Morales A.J., Maldonado-Montoro M., de la Plata J.E.M., Ramirez C.P., Daddaoua A., Payer C.A., Exposito-Ruiz M., Collado C.G. (2019). FCGR2A/FCGR3A Gene Polymorphisms and Clinical Variables as Predictors of Response to Tocilizumab and Rituximab in Patients With Rheumatoid Arthritis. J. Clin. Pharmacol..

[69] Quartuccio L., Fabris M., Pontarini E., Salvin S., Zabotti A., Benucci M., Manfredi M., Biasi D., Ravagnani V., Atzeni F. (2013). The 158VV Fcgamma receptor 3A genotype is associated with response to rituximab in rheumatoid arthritis: Results of an Italian multicentre study. Ann. Rheum. Dis..

[70] Ruyssen-Witrand A., Rouanet S., Combe B., Dougados M., Le Loët X., Sibilia J., Tebib J., Mariette X., Constantin A. (2012). Fcγ receptor type IIIA polymorphism influences treatment outcomes in patients with rheumatoid arthritis treated with rituximab. Ann. Rheum. Dis..

[71] Maldonado-Montoro M., Cañadas-Garre M., González-Utrilla A., Calleja-Hernández M. (2016). Influence of IL6R gene polymorphisms in the effectiveness to treatment with tocilizumab in rheumatoid arthritis. Pharmacogenomics J..

[72] Luxembourger C., Ruyssen-Witrand A., Ladhari C., Rittore C., Degboe Y., Maillefert J.-F., Gaudin P., Marotte H., Wendling D., Jorgensen C. (2019). A single nucleotide polymorphism of IL6-receptor is associated with response to tocilizumab in rheumatoid arthritis patients. Pharmacogenomics J..

[73] Enevold C., Baslund B., Linde L., Josephsen N.L., Tarp U., Lindegaard H., Jacobsen S., Nielsen C.H. (2014). Interleukin-6-receptor polymorphisms rs12083537, rs2228145, and rs4329505 as predictors of response to tocilizumab in rheumatoid arthritis. Pharmacogenetics Genom..

[74] Camp N.J., Cox A., di Giovine F.S., McCabe D., Rich W., Duff G.W. (2005). Evidence of a pharmacogenomic response to interleukin-l receptor antagonist in rheumatoid arthritis. Genes Immun..

[75] Pete N.M., Montoro M.d.M.M., Ramírez C.P., Martínez F.M., de la Plata J.E.M., Daddaoua A., Morales A.J. (2021). Influence of the FCGR2A rs1801274 and FCGR3A rs396991 Polymorphisms on Response to Abatacept in Patients with Rheumatoid Arthritis. J. Pers. Med..

[76] Gazeau P., Alegria G.C., Devauchelle-Pensec V., Jamin C., Lemerle J., Bendaoud B., Brooks W.H., Saraux A., Cornec D., Renaudineau Y. (2017). Memory B Cells and Response to Abatacept in Rheumatoid Arthritis. Clin. Rev. Allergy Immunol..

[77] Yoon S.M., Haritunians T., Chhina S., Liu Z., Yang S., Landers C., Li D., Ye B.D., Shih D., Vasiliauskas E.A. (2017). Colonic Phenotypes Are Associated with Poorer Response to Anti-TNF Therapies in Patients with IBD. Inflamm. Bowel Dis..

[78] E Burke K., Khalili H., Garber J.J., Haritunians T., McGovern D.P.B., Xavier R.J., Ananthakrishnan A.N. (2018). Genetic Markers Predict Primary Nonresponse and Durable Response to Anti–Tumor Necrosis Factor Therapy in Ulcerative Colitis. Inflamm. Bowel Dis..

[79] Bank S., Julsgaard M., Abed O.K., Burisch J., Brodersen J.B., Pedersen N.K., Gouliaev A., Ajan R., Rasmussen D.N., Grauslund C.H. (2019). Polymorphisms in the NFkB, TNF-alpha, IL-1beta, and IL-18 pathways are associated with response to anti-TNF therapy in Danish patients with inflammatory bowel disease. Aliment. Pharmacol. Ther..

[80] Urabe S., Isomoto H., Ishida T., Maeda K., Inamine T., Kondo S., Higuchi N., Sato K., Uehara R., Yajima H. (2015). Genetic Polymorphisms ofIL-17FandTRAF3IP2Could Be Predictive Factors of the Long-Term Effect of Infliximab against Crohn’s Disease. BioMed Res. Int..

[81] Salvador-Martin S., Bossacoma F., Pujol-Muncunill G., Navas-Lopez V.M., Gallego-Fernandez C., Viada J., Munoz-Codoceo R., Magallares L., Martinez-Ojinaga E., Moreno-Alvarez A. (2020). Genetic Predictors of Long-term Response to Antitumor Necrosis Factor Agents in Pediatric Inflammatory Bowel Disease. J. Pediatr. Gastroenterol. Nutr..

[82] Netz U., Carter J.V., Eichenberger M.R., Dryden G.W., Pan J., Rai S.N., Galandiuk S. (2017). Genetic polymorphisms predict response to anti-tumor necrosis factor treatment in Crohn’s disease. World J. Gastroenterol..

[83] Matsuoka K., Hamada S., Shimizu M., Nanki K., Mizuno S., Kiyohara H., Arai M., Sugimoto S., Iwao Y., Ogata H. (2018). Factors predicting the therapeutic response to infliximab during maintenance therapy in Japanese patients with Crohn’s disease. PLoS ONE.

[84] Louis E., El Ghoul Z., Vermeire S., Dall’Ozzo S., Rutgeerts P., Paintaud G., Belaiche J., De Vos M., Van Gossum A., Colombel J. (2004). Association between polymorphism in IgG Fc receptor IIIa coding gene and biological response to infliximab in Crohn’s disease. Aliment. Pharmacol. Ther..

[85] Papamichaela K., Gazoulib M., Karakoidasa C., Panayotouc I., Roma-Giannikouc E., Mantzarisa G.J. (2011). Association of TNF and FcγRIIA gene polymorphisms with differential response to infliximab in a Greek cohort of crohn’s disease patients. Ann Gastroenterol..

[86] Curci D., Lucafò M., Cifù A., Fabris M., Bramuzzo M., Martelossi S., Franca R., Decorti G., Stocco G. (2021). Pharmacogenetic variants of infliximab response in young patients with inflammatory bowel disease. Clin. Transl. Sci..

[87] Louis E.J., Watier H.E., Schreiber S., Hampe J., Taillard F., Olson A., Thorne N., Zhang H., Colombel J.-F. (2006). Polymorphism in IgG Fc receptor gene FCGR3A and response to infliximab in Crohn’s disease: A subanalysis of the ACCENT I study. Pharmacogenetics Genom..

[88] Hoffmann P., Lamerz D., Hill P., Kirchner M., Gauss A. (2021). Gene Polymorphisms of NOD2, IL23R, PTPN2 and ATG16L1 in Patients with Crohn’s Disease: On theWay to Personalized Medicine?. Genes.

[89] Potter C., Cordell H.J., Barton A., Daly A.K., Hyrich K.L., Mann D.A., Morgan A.W., Wilson A.G., Isaacs J.D., the Biologics in Rheumatoid Arthritis Genetics and Genomics Study Syndicate (BRAGGSS) (2010). Association between anti-tumour necrosis factor treatment response and genetic variants within the TLR and NF B signalling pathways. Ann. Rheum. Dis..

[90] Zervou M.I., Myrthianou E., Flouri I., Plant D., Chlouverakis G., Castro-Giner F., Rapsomaniki P., Barton A., Boumpas D.T., Sidiropoulos P. (2013). Lack of Association of Variants Previously Associated with Anti-TNF Medication Response in Rheumatoid Arthritis Patients: Results from a Homogeneous Greek Population. PLoS ONE.

[91] Ferreiro-Iglesias A., Montes A., Perez-Pampin E., Cañete J.D., Raya E., Magro-Checa C., Vasilopoulos Y., Sarafidou T., Caliz R., A Ferrer M. (2015). Replication of PTPRC as genetic biomarker of response to TNF inhibitors in patients with rheumatoid arthritis. Pharmacogenomics J..

[92] Vasilopoulos Y., Bagiatis V., Stamatopoulou D., Zisopoulos D., Alexiou I., Sarafidou T., Settas L., Sakkas L., Mamouris Z. (2011). Association of anti-CCP positivity and carriage of TNFRII susceptibility variant with anti-TNF-α response in rheumatoid arthritis. Clin. Exp. Rheumatol..

[93] Jančić I., Šefik-Bukilica M., Živojinović S., Damjanov N., Spasovski V., Kotur N., Klaassen K., Pavlović S., Bufan B., Arsenović-Ranin N. (2015). Influence Of Promoter Polymorphisms Of The Tnf-α (-308g/A) And IL-6 (-174g/C) Genes On Therapeutic Response To Etanercept In Rheumatoid Arthritis. J. Med Biochem..

[94] Cuchacovich M., Soto L., Edwardes M., Gutierrez M., Llanos C., Pacheco D., Sabugo F., Alamo M., Fuentealba C., Villanueva L. (2006). Tumour necrosis factor (TNF)α −308 G/G promoter polymorphism and TNFα levels correlate with a better response to adalimumab in patients with rheumatoid arthritis. Scand. J. Rheumatol..

[95] Padyukov L., Lampa J., Heimbürger M., Ernestam S., Cederholm T., Lundkvist I., Andersson P., Hermansson Y., Harju A., Klareskog L. (2003). Genetic markers for the efficacy of tumour necrosis factor blocking therapy in rheumatoid arthritis. Ann. Rheum. Dis..

[96] Guis S., Balandraud N., Bouvenot J., Auger I., Toussirot E., Wendling D., Mattei J.-P., Nogueira L., Mugnier B., Legeron P. (2007). Influence of −308 A/G polymorphism in the tumor necrosis factor α gene on etanercept treatment in rheumatoid arthritis. Arthritis Rheum..

[97] Pinto J.A., Rego I., Rodríguez-Gomez M., Cañete J.D., Fernandez-López C., Freire M., Fernandez-Sueiro J.L., Sanmarti R., Blanco F.J. (2008). Polymorphisms in genes encoding tumor necrosis factor-α and HLA-DRB1 are not associated with response to infliximab in patients with rheumatoid arthritis. J Rheumatol..

[98] Maxwell J.R., Potter C., Hyrich K.L., Barton A., Worthington J., Isaacs J.D., Morgan A.W., Wilson A.G. (2008). Braggss Association of the tumour necrosis factor-308 variant with differential response to anti-TNF agents in the treatment of rheumatoid arthritis. Hum. Mol. Genet..

[99] Miceli-Richard C., Comets E., Verstuyft C., Tamouza R., Loiseau P., Ravaud P., Kupper H., Becquemont L., Charron D., Mariette X. (2007). A single tumour necrosis factor haplotype influences the response to adalimumab in rheumatoid arthritis. Ann. Rheum. Dis..

[100] Marotte H., Arnaud B., Diasparra J., Zrioual S., Miossec P. (2008). Association between the level of circulating bioactive tumor necrosis factor α and the tumor necrosis factor α gene polymorphism at −308 in patients with rheumatoid arthritis treated with a tumor necrosis factor α inhibitor. Arthritis Rheum..

[101] Louis E., Vermeire S., Rutgeerts P., De Vos M., Van Gossum A., Pescatore P., Fiasse R., Pelckmans P., Reynaert H., D’Haens G. (2002). Inflammatory Bowel Disease A Positive Response to Infliximab in Crohn Disease: Association with a Higher Systemic Inflammation Before Treatment But Not With -308 TNF Gene Polymorphism. Scand. J. Gastroenterol..

[102] Dideberg V., Théâtre E., Farnir F., Vermeire S., Rutgeerts P., De Vos M., Belaiche J., Franchimont D., Van Gossum A., Louis E. (2006). The TNF/ADAM 17 system: Implication of an ADAM 17 haplotype in the clinical response to infliximab in Crohn’s disease. Pharmacogenetics Genom..

[103] López-Hernández R., Valdés M., Campillo J.A., Martínez-Garcia P., Salama H., Salgado G., Boix F., Moya-Quiles M.R., Minguela A., Sánchez-Torres A. (2013). Genetic polymorphisms of tumour necrosis factor alpha (TNF-α) promoter gene and response to TNF-α inhibitors in Spanish patients with inflammatory bowel disease. Int. J. Immunogenetics.

[104] Duricova D., Pedersen N., Lenicek M., Hradsky O., Bronsky J., Adamcova M., Elkjaer M., Andersen P.S., Vitek L., Larsen K. (2009). Infliximab dependency in children with Crohn’s disease. Aliment. Pharmacol. Ther..

[105] Mendrinou E., Patsatsi A., Zafiriou E., Papadopoulou D., Aggelou L., Sarri C., Mamuris Z., Kyriakou A., Sotiriadis D., Roussaki-Schulze A. (2016). FCGR3A-V158F polymorphism is a disease-specific pharmacogenetic marker for the treatment of psoriasis with Fc-containing TNFα inhibitors. Pharmacogenomics J..

[106] Julià M., Guilabert A., Lozano F., Suarez-Casasús B., Moreno N., Carrascosa J.M., Ferrándiz C., Pedrosa E., Alsina-Gibert M., Mascaró J.M. (2013). The Role of Fcγ Receptor Polymorphisms in the Response to Anti–Tumor Necrosis Factor Therapy in Psoriasis. JAMA Dermatol..

[107] Batalla A., Coto E., Coto-Segura P. (2015). Influence of Fcγ Receptor Polymorphisms on Response to Anti–Tumor Necrosis Factor Treatment in Psoriasis. JAMA Dermatol..

[108] Prieto-Perez R., Solano-Lopez G., Cabaleiro T., Roman M., Ochoa D., Talegon M., Baniandres O., Lopez Estebaranz J.L., de la Cueva P., Dauden E. (2015). The polymorphism rs763780 in the IL-17F gene is associated with response to biological drugs in patients with psoriasis. Pharmacogenomics.

[109] Caldarola G., Sgambato A., Fanali C., Moretta G., Farina M., Lucchetti D., Peris K., De Simone C. (2016). HLA-Cw6 allele, NFkB1 and NFkBIA polymorphisms play no role in predicting response to etanercept in psoriatic patients. Pharmacogenetics Genom..

[110] Mugnier B., Balandraud N., Darque A., Roudier C., Roudier J., Reviron D. (2003). Polymorphism at position −308 of the tumor necrosis factor α gene influences outcome of infliximab therapy in rheumatoid arthritis. Arthritis Rheum..

[111] Cuchacovich M., Ferreira L., Aliste M., Soto L., Cuenca J., Cruzat A., Gatica H., Schiattino I., Pérez C., Aguirre A. (2004). Tumour necrosis factor?? (TNF??) levels and influence of ?308 TNF?? promoter polymorphism on the responsiveness to infliximab in patients with rheumatoid arthritis. Scand. J. Rheumatol..

[112] Chatzikyriakidou A., Georgiou I., Voulgari P.V., Venetsanopoulou A.I., Drosos A.A. (2007). Combined tumour necrosis factor- and tumour necrosis factor receptor genotypes could predict rheumatoid arthritis patients’ response to anti-TNF- therapy and explain controversies of studies based on a single polymorphism. Rheumatology.

[113] Morales-Lara M.J., Cañete J.D., Torres-Moreno D., Hernández M.V., Pedrero F., Celis R., García-Simón M.S., Conesa-Zamora P. (2012). Effects of polymorphisms in TRAILR1 and TNFR1A on the response to anti-TNF therapies in patients with rheumatoid and psoriatic arthritis. Jt. Bone Spine.

[114] Matsukura H., Ikeda S., Yoshimura N., Takazoe M., Muramatsu M. (2008). Genetic polymorphisms of tumour necrosis factor receptor superfamily 1A and 1B affect responses to infliximab in Japanese patients with Crohn’s disease. Aliment. Pharmacol. Ther..

[115] Medrano L., Taxonera C., Márquez A., Acosta M.B.-D., Gómez-García M., González-Artacho C., Pérez-Calle J., Bermejo F., Lopez-Sanromán A., Arranz M. (2013). Role of TNFRSF1B polymorphisms in the response of Crohn’s disease patients to infliximab. Hum. Immunol..

[116] Toonen E.J.M., Coenen M.J.H., Kievit W., Fransen J., Eijsbouts A.M., Scheffer H., Radstake T.R.D.J., Creemers M.C.W., Rooij D.-J.R.A.M.d., van Riel P.L.C.M. (2008). The tumour necrosis factor receptor superfamily member 1b 676T>G polymorphism in relation to response to infliximab and adalimumab treatment and disease severity in rheumatoid arthritis. Ann. Rheum. Dis..

[117] Pers Y.-M., Cadart D., Rittore C., Ravel P., Daïen V., Fabre S., Jorgensen C., Touitou I. (2014). TNFRII polymorphism is associated with response to TNF blockers in rheumatoid arthritis patients seronegative for ACPA. Jt. Bone Spine.

[118] Canet L.M., Filipescu I., Cáliz R., Lupiañez C.B., Canhão H., Escudero A., Segura-Catena J., Soto-Pino M.J., Ferrer M.A., García A. (2015). Genetic variants within the TNFRSF1B gene and susceptibility to rheumatoid arthritis and response to anti-TNF drugs. Pharmacogenetics Genom..

[119] Mascheretti S., Hampe J., Kühbacher T., Herfarth H., Krawczak M., Fölsch U.R., Schreiber S. (2002). Pharmacogenetic investigation of the TNF/TNF-receptor system in patients with chronic active Crohn’s disease treated with infliximab. Pharmacogenomics J..

[120] Steenholdt C., Enevold C., Ainsworth M.A., Brynskov J., Thomsen O., Bendtzen K. (2012). Genetic polymorphisms of tumour necrosis factor receptor superfamily 1b and fas ligand are associated with clinical efficacy and/or acute severe infusion reactions to infliximab in Crohn’s disease. Aliment. Pharmacol. Ther..

[121] Antonatos C., Stavrou E.F., Evangelou E., Vasilopoulos Y. (2021). Exploring pharmacogenetic variants for predicting response to anti-TNF therapy in autoimmune diseases: A meta-analysis. Pharmacogenomics.

[122] O’Rielly D.D., Roslin N.M., Beyene J., Pope A., Rahman P. (2009). TNF-α −308 G/A polymorphism and responsiveness to TNF-α blockade therapy in moderate to severe rheumatoid arthritis: A systematic review and meta-analysis. Pharmacogenomics J..

[123] Bank S., Andersen P.S., Burisch J., Pedersen N., Roug S., Galsgaard J., Turino S.Y., Brodersen J.B., Rashid S., Rasmussen B.K. (2014). Polymorphisms in the Inflammatory Pathway Genes TLR2, TLR4, TLR9, LY96, NFKBIA, NFKB1, TNFA, TNFRSF1A, IL6R, IL10, IL23R, PTPN22, and PPARG Are Associated with Susceptibility of Inflammatory Bowel Disease in a Danish Cohort. PLoS ONE.

[124] I Robinson J., Barrett J.H., Taylor J.C., Naven M., Corscadden D., Barton A., Wilson A.G., Emery P., Isaacs J.D., Morgan A.W. (2009). Dissection of the FCGR3A association with RA: Increased association in men and with autoantibody positive disease. Ann. Rheum. Dis..

[125] Asano K., Matsushita T., Umeno J., Hosono N., Takahashi A., Kawaguchi T., Matsumoto T., Matsui T., Kakuta Y., Kinouchi Y. (2009). A genome-wide association study identifies three new susceptibility loci for ulcerative colitis in the Japanese population. Nat. Genet..

[126] Hatjiharissi E., Xu L., Santos D.D., Hunter Z.R., Ciccarelli B.T., Verselis S., Modica M., Cao Y., Manning R.J., Leleu X. (2007). Increased natural killer cell expression of CD16, augmented binding and ADCC activity to rituximab among individuals expressing the FcγRIIIa-158 V/V and V/F polymorphism. Blood J. Am. Soc. Hematol..

[127] Koene H.R., Kleijer M., Algra J., Roos D., EGKr von dem Borne A., de Haas M. (1997). FcγRIIIa-158V/F polymorphism influences the binding of IgG by natural killer cell FcγRIIIa, independently of the FcγRIIIa-48L/R/H phenotype. Blood J. Am. Soc. Hematol..

[128] Robinson J.I., Yusof Y.M., Davies V., Wild D., Morgan M., Taylor J.C., El-Sherbiny Y., Morris D.L., Liu L., Rawstron A.C. (2022). Comprehensive genetic and functional analyses of Fc gamma receptors influence on response to rituximab therapy for autoimmunity. EBioMedicine.

[129] Isaacs J.D., Cohen S.B., Emery P., Tak P.P., Wang J., Lei G., Williams S., Lal P., Read S.J. (2012). Effect of baseline rheumatoid factor and anticitrullinated peptide antibody serotype on rituximab clinical response: A meta-analysis. Ann. Rheum. Dis..

[130] Sandberg M.E.C., Bengtsson C., Källberg H., Wesley A., Klareskog L., Alfredsson L., Saevarsdottir S. (2014). Overweight decreases the chance of achieving good response and low disease activity in early rheumatoid arthritis. Ann. Rheum. Dis..

[131] Ko S.-H., Chi C.-C., Yeh M.-L., Wang S.-H., Tsai Y.-S., Hsu M.-Y. (2019). Lifestyle changes for treating psoriasis. Emergencias.

[132] Upala S., Sanguankeo A. (2015). Effect of lifestyle weight loss intervention on disease severity in patients with psoriasis: A systematic review and meta-analysis. Int. J. Obes..

[133] Højgaard P., Glintborg B., Hetland M.L., Hansen T.H., Lage-Hansen P.R., Petersen M.H., Holland-Fischer M., Nilsson C., Loft A.G., Andersen B.N. (2014). Association between tobacco smoking and response to tumour necrosis factor α inhibitor treatment in psoriatic arthritis: Results from the DANBIO registry. Ann. Rheum. Dis..

[134] Schwarz C.W., Loft N., Rasmussen M.K., Nissen C.V., Dam T.N., Ajgeiy K.K., Egeberg A., Skov L. (2021). Predictors of Response to Biologics in Patients with Moderate-to-severe Psoriasis: A Danish Nationwide Cohort Study. Acta Derm. Venereol..

