# Supplementary material for: The Association between Genetics and Response to Treatment with Biologics in Patients with Psoriasis, Psoriatic Arthritis, Rheumatoid Arthritis, and Inflammatory Bowel Diseases: A Systematic Review and Meta-Analysis
Source: Int J Mol Sci. 2024 May 26;25(11):5793. doi: 10.3390/ijms25115793 (PMC11171831; doi:10.3390/ijms25115793)
Supplement: Supplementary file 1 [file ijms-25-05793-s001.zip › Supplementary Table S1.pdf]

**Supplementary Table S1.** Search string		
(i)	Drugs/biologics	((adalimumab OR etanercept OR infliximab OR certolizumab pegol OR golimumab) OR (ustekinumab) OR (secukinumab OR ixekizumab OR brodalumab) OR (guselkumab OR risankizumab OR tildrakizumab) OR (abatacept) OR (rituximab) OR (anakinra) OR (tocilizumab OR sarilumab))
(ii)	Diseases	((Psoriasis) OR (rheumatoid arthritis OR RA) OR (Inflammatory Bowel Disease OR IBD OR Crohn's disease OR Colitis ulcerosa OR Colitis OR ulcerative colitis))
(iii)	Genetic variants	(Polymorphism OR SNPs OR pharmacogenetics OR pharmacogenomics or genetic biomarker)
**Final search string**		
((adalimumab OR etanercept OR infliximab OR certolizumab pegol OR golimumab) OR (ustekinumab) OR (secukinumab OR ixekizumab OR brodalumab) OR (guselkumab OR risankizumab OR tildrakizumab) OR (abatacept) OR (rituximab) OR (anakinra) OR (tocilizumab OR sarilumab))<br><br>AND<br><br>((Psoriasis) OR (rheumatoid arthritis OR RA) OR (Inflammatory Bowel Disease OR IBD OR Crohn's disease OR Colitis ulcerosa OR Colitis OR ulcerative colitis))<br><br>AND<br><br>(Polymorphism OR SNPs OR pharmacogenetics OR pharmacogenomics or genetic biomarker)		
