# Supplementary material for: The Association between Genetics and Response to Treatment with Biologics in Patients with Psoriasis, Psoriatic Arthritis, Rheumatoid Arthritis, and Inflammatory Bowel Diseases: A Systematic Review and Meta-Analysis
Source: Int J Mol Sci. 2024 May 26;25(11):5793. doi: 10.3390/ijms25115793 (PMC11171831; doi:10.3390/ijms25115793)
Supplement: Supplementary file 1 [file ijms-25-05793-s001.zip › Supplementary Table S2.pdf]

Psoriasis supplementary Table S2

		Anti-TNF				Anti-IL12/23	Anti-IL-17
Gene (rs number)	Chromosome number:location	Adalimumab	Infliximab	Etanercept	Anti-TNF overall	Ustekinumab	Secukinumab
ADAM33 (rs2787094) C/G	chr20:3668514					Association<br>One study with 69 patients found an association with response.<br>OR (95% CI): 6.95(1.04-46.69). P=0.046[1]<br><br>No association<br>No studies	
ADRA2A (rs553668) A/G	chr10:111079821				Association<br>One study with 65 patients found an association with response.<br>OR: 0.1019 P=3.14E-05[2]<br><br>No association<br>No studies		
AG2 (rs3784240) G/A	chr14:105156924				Association<br>One study with 65 patients found an association with response.<br>OR: 9.71 P=9.64E-06[2]<br><br>No association<br>No studies		

TNFRSF1A (rs191190) C/T	chr1:48608973				Association<br>Two studies found an association with response.<br><br>Ovejero-Benito et al: n=95. OR (95% CI): 2.71 (0.88-8.35). P=0.024[3]<br><br>Prieto-Pérez et al: n=144. OR (95% CI): 3.16 (0.95-10.47). P= 0.039[4]<br><br>No association<br>No studies	Association<br>One study with 69 patients found an association with response.<br>OR (95% CI): 38.29 (1.64-513.06) P=0.003[1]<br><br>No association<br>No studies	
AKAP13 (rs28461892) C/A	chr15:85633957				Association<br>One study with 243 patients found an association with response.<br>OR (95% CI): 7.61 (3.02–19.18)<br>P= 9.43×10^{-7} [5]<br><br>No association<br>No studies		
AP4E1 (rs4775912) T/C	chr15:50988782				Association<br>One study with 95 patients found an association with response.<br>OR (95% CI): 0.33 (0.11- 0.99) P= 0.027[3]<br><br>No association<br>No studies		

C17orf51 (rs1975974) A/G	chr17:22180454			Association<br>One study with 78 patients found an association with response.<br>OR (95% CI): 0.17 (0.04–00.62). P= 0.002[6]<br><br>No association<br>No studies	Association<br>One study with 144 patients found an association with response.<br>OR (95% CI): 0.29 (0.12-0.70). P=0.003[4]<br><br>No association<br>No studies	Association<br>One study with 69 patients found an association with response.<br>OR (95% CI): 0.07 (0.01-0.53) P= 0.000[1]<br><br>No association<br>No studies	
C6orf10 (rs2022544) C/T	chr6:32353227				Association<br>One study with 144 patients found an association with response.<br>OR (95% CI): 2.75 (1.10-6.88) P=0.031[4]<br><br>No association<br>No studies		
C6orf10 (rs2073048) G/A	chr6:32367656				Association<br>One study with 144 patients found an association with response.<br>OR (95% CI): 2.75 (1.10-6.88) P=0.031[4]<br><br>No association<br>No studies		
C9orf72 (rs774359) T/C	chr9:27561051				Association<br>One study with 144 patients found an association with response. OR (95%	Association<br>One study with 69 patients found an association with response. OR (95%	

					CI): 2.85 (1.00-8.06). P= 0.041[4]<br><br>No association<br>No studies	CI): 0.29 (0.09-0.98). P= 0.041[1]<br><br>No association<br>No studies	
CARD14 (rs11652075) C/T	chr17:80205094				Association<br>One study with 116 patients found an association with response.<br>OR (95% CI)= 3.71 (1.30-10.51). p=0.01[7]<br><br>No association<br>No studies		
CD84 (rs6427528) A/G	chr1:160546518	Association<br>No studies<br><br>No association<br>One study with 121 patients found no association with response. P=0.08[8]		Association<br>One study with 161 patients found an association with response. n=161. P=0.025[8]<br><br>No association<br>No studies		Association<br>No studies<br><br>No association<br>One study with 66 patients found no association with response. P=0.15[8]	
CDH12 (rs1487419) G/A	chr5:22626006				Association<br>One study with 243 patients found an association with response.<br>OR (95% CI): 5.20 (2.52–10.74).<br>$P = 1.55 \times 10^{-6}$ [5]<br><br>No association<br>No studies		

CDH12<br>(rs77497886) G/T	chr5:22616557				Association<br>One study with 243 patients found an association with response.<br>OR (95% CI): 5.20 (2.52–10.74)<br>P= 1.55 × 10–6[5]**<br><br>No association<br>No studies		
CDH12<br>(rs80063785) A/G	chr5:22398459				Association<br>One study with 243 patients found an association with response.<br>P= 1.03 × 10–7[5]**<br><br>No association<br>No studies		
CDH23<br>(rs10823825) T/C	chr10:71694880				Association<br>One study with 65 patients found an association with response.<br>OR: 9.442. P= 1.76E-05[2]<br><br>No association<br>No studies		
CDKAL1<br>(rs6908425) T/C	chr6:20728500				Association<br>Three studies found an association with response.<br><br>Prieto-Pérez et al: n=144. OR (95% CI): 2.60 (1.19-5.66). P= 0.014[4]		

					Ovejero-Benito et al: n=95. OR (95% CI): 0.41 (0.15-1.09). P=0.050[3]<br><br>Coto-Segura et al: n=116<br>OR (95% CI): 3.14 (1.40–7.05). P=0.005. [9]<br><br>No association<br>No studies		
CHUK (rs11591741) G/C	chr10:100216744				Association<br>No studies<br><br>No association<br>One study with 376 patients found no association with response. OR (95% CI): 0.69 (0.33-1.47). P=0.35[10]	Association<br>Two studies found an association with response.<br>Loft et al: n=230. OR (95% CI): 0.29 (0.09–0.88). P=0.029[10]<br><br>Prieto-Pérez et al: n=69<br>OR (95% CI): 0.03 (0.00-0.47). P=0.002[1]<br><br>No association<br>No studies	
CTLA4 (rs3087243) G/A	chr2:203874196				Association<br>One study with 144 patients found an association with response.<br>OR (95% CI): 0.41 (0.17-0.98). P=0.048[4]<br><br>No association<br>No studies		

CTNNA2 (rs11126740) G/A	chr2:79673969			Association<br>One study with 78 patients found an association with response.<br>OR (95% CI): 5.89 (1.26–27.52).<br>P=0.022[6]<br><br>No association<br>No studies	Association<br>One study with 144 patients found an association with response.<br>OR (95% CI): 6.14 (1.91-19.78).<br>P=0.0018[4]<br><br>No association<br>No studies		
DDX58_v1 (rs34085293) T/G	chr9:32534716						Association<br>One study with 62 patients found an association with response.<br>OR (95% CI): 10.8 (1.23-94.9).<br>P= 0.0078[11]<br><br>No association<br>No studies
ERAP1 (rs151823) A/C	chr5:96824289				Association<br>One study with 22 patients found an association with response.<br>P=0.026[12]<br><br>No association<br>No studies		
ERAP1 (rs26653) C/G	chr5:96803547					Association<br>One study with 22 patients found an association with response.<br>P=0.016[12]	

						No association<br>No studies	
ERAP1 (rs27524) A/G	chr5:96766240	Association<br>No studies<br><br>No association<br>One study with 121 patients found no association with response. P=0.30[8]		Association<br>One study with 158 patients found no association with response. P=0.05[8]<br><br>No association<br>No studies		Association<br>No studies<br><br>No association<br>One study with 66 patients found no association with response. P=0.10[8]	
FCGR2A (rs1801274) G/A#	chr1:161509955			Association<br>No studies<br><br>No association<br>Two studies found no association with response<br><br>Mendrinou et al: n=55 P=0.500[13]<br><br>Batalla et al: n=30. P=0.51.[14]	Association<br>One study with 144 patients found an association with response. OR (95% CI): 5.22 (1.17-23.37). P=0.009[4]<br><br>No association<br>Three studies found no association with response.<br><br>Mendrinou et al: n=100. P=0.749[13]<br><br>Julia et al: n=70. P=0.52[15]<br><br>Batalla et al: n=115. P=NS[14]		
FCGR3A (rs396991) A/C	chr1:161544752			Association<br>Two studies found	Association<br>One study with 100 patients found an		

				an association with response.<br><br>Mendrinou et al: n=55. P=0.017[13]<br><br>Batalla et al: n=30. P=0.04.[14]<br><br>No association<br>No studies	association with response. P=0.019[13]<br><br>No association<br>Two studies found no association with response.<br><br>Julià et al: n=70. P=0.13[15]<br><br>Batalla et al: n=115. P=NS.[14]		
FOXP3 (rs2280883) T/C	chrX:49252667				Association<br>One study with 144 patients found an association with response.<br>OR (95% CI): 3.60 (1.12-11.53). P= 0.024[4]<br><br>No association<br>No studies		
FOXP3 (rs3761548) G/T	chrX:49261784				Association<br>One study with 144 patients found an association with response.<br>OR (95% CI): 4.15 (1.29-13.33). P=0.012[4]<br><br>No association<br>No studies		

GBP6 (rs928655) A/G	chr1:89384015			Association<br>One study with 78 patients found an association with response. OR (95% CI): 0.23 (0.07–00.74). P=0.010[6]<br><br>No association<br>No studies	Association<br>One study with 144 patients found an association with response. OR (95% CI): 0.34 (0.11-1.02). P= 0.042[4]<br><br>No association<br>No studies	Association<br>One study with 69 patients found an association with response. OR (95% CI): 4.12 (1.15-14.79). P= 0.026[1]<br><br>No association<br>No studies	
GUCY1B3 (rs2219538) G/A	chr4:155762129				Association<br>One study with 65 patients found an association with response.<br>OR: 13.75) P= 1.59E-05[2]<br><br>No association<br>No studies		
HNRNPKP3 (rs11037342) A/C	chr11:43196874				Association<br>One study with 243 patients found an association with response. OR (95% CI): 3.34 (1.98–5.61). P= 3.09 × 10–6[5]**<br><br>No association<br>No studies		
HNRNPKP3 (rs11037360) G/A	chr11:43218254				Association<br>One study with 243 patients found an association with response. OR (95%		

					CI): 3.43 (2.04–5.76).<br>P= 1.58 × 10–6[5]**<br><br>No association<br>No studies		
HNRNPKP3<br>(rs145304743)<br>G/T	chr11:43226378				Association<br>One study with 243 patients found an association with response. OR (95% CI): 3.34 (1.98–5.61).<br>P= 3.09 × 10–6[5]**<br><br>No association<br>No studies		
HNRNPKP3<br>(rs1845821) T/C	chr11:43237447				Association<br>One study with 243 patients found an association with response.<br>OR (95% CI): 3.026 (1.84–4.97)<br>P= 8.15 × 10–6[5]**<br><br>No association<br>No studies		
HNRNPKP3<br>(rs7481533) T/C	chr11:43201797				Association<br>One study with 243 patients found an association with response.<br>OR (95% CI): 3.34 (1.98–5.61)<br>P= 3.09 × 10–6[5]**<br><br>No association<br>No studies		

HPC5 (rs2395029) T/G	chr6:31464003				Association<br>One study with 90 patients found an association with response.<br>P=0.039[3]<br><br>No association<br>No studies		
HTR2A (rs6311) C/T	chr13:46897343			Association<br>One study with 68 patients found an association with non-response.<br>OR (95% CI): 4.50 (0.92–22.00).<br>P= 0.036[6]<br><br>No association<br>No studies	Association<br>Two studies found an association with response and poor response:<br><br>Ovejero-Benito et al: n: 90. OR (95% CI): 7.19 (0.89-57.76). P=0.018[3]<br><br>Prieto-Pérez, et al: n=144. OR (95% CI): 4.53 (1.28-16.06). P= 0.007[4]<br><br>No association<br>No studies	Association<br>One study with 69 patients found an association with response. OR (95% CI): 20.04 (1.32-303.34). P=0.003[1]<br><br>No association<br>No studies	
IFIH1 (rs17716942) T/C	chr2:162404181	Association<br>No studies<br><br>No association<br>One study with 121 patients found no association with response.<br>P=0.12[8]		Association<br>No studies<br><br>No association<br>One study with 159 patients found no association with response.<br>P=0.72[8]	Association<br>One study with 144 patients found an association with response.<br>OR (95% CI): 2.90 (1.12-7.54). P=0.032[4]<br><br>No association<br>No studies	Association<br>No studies<br><br>No association<br>One study with 66 patients found no association with response.<br>P=0.43[8]	

IFNG (rs2430561) T/A	chr12:68158742				Association<br>No studies<br><br>No association<br>One study with 376 patients found no association with response. OR (95% CI): 0.83 (0.38-1.82). P=0.64[10]	Association<br>One study with 230 patients found an association with response.<br>OR (95% CI): 3.04 (1.19–7.78). P=0.020[10]<br><br>No association<br>No studies	
IL-10 (rs1800896) T/C	chr1:206773552				Association<br>One study with 95 patients found an association with response.<br>OR (95% CI): 2.19 (1.07-4.47) P=0.028[3]<br><br>No association<br>No studies		
IL-13 (rs848) A/C	chr5:132660808					Association<br>One study with 69 patients found an association with response. OR (95% CI): 3.6 (1.10-11.86). P= 0.035[1]<br><br>No association<br>No studies	
IL-17F (rs763780) T/C	chr6:52236941	Association<br>One study with 67 patients found an association with response. OR (95%	Association<br>One study with 36 patients found an association with response. P=0.023[16]	Association<br>No studies<br><br>No association<br>One study with 77 patients found no	Association<br>No studies<br><br>No association<br>One study with 194 patients found no	Association<br>One study with 70 patients found an association with response. OR (95%	

		CI): 14.00 (2.15-91.12). P=0 0.0044[16]<br><br>No association<br>No studies	No association<br>No studies	association with response. OR (95% CI): 3.18 (0.59-17.23). P= 0.190[16]	association with response. OR (95% CI): 3 1.26 (0.41-3.88). P= 0.690[16]	CI): 12.23 (1.17-127.36). P= 0.022[16]<br><br>No association<br>No studies	
IL10 (rs1800871) A/G	chr1:206773289				Association<br>One study with 144 patients found an association with response.<br>P=0.050[4]<br><br>No association<br>No studies		
IL10 (rs1800872) T/G	chr1:206773062				Association<br>One study with 144 patients found an association with response.<br>P=0.048[4]<br><br>No association<br>One study with 376 patients found no association with response.<br>OR (95% CI): 0.94 (0.46-1.96), P=0.88[10]	Association<br>No studies<br><br>No association<br>One study with 230 patients found no association with response.<br>OR (95% CI): 0.96 (0.38-2.42), P=0.93[10]	
IL10 (rs3024505) G/A	chr1:206766559				Association<br>No studies<br><br>No association<br>One study with 376 patients found no association with response.<br>OR (95% CI): 0.95	Association<br>One study with 230 patients found an association with response.<br>OR (95% CI): 0.32 (0.13–0.81).<br>P=0.015[10]	

					(0.47-1.94). P=0.89[10]	No association<br>No studies	
IL12B<br>(rs2546890) A/G	chr5:159332892			Association<br>One study with 78 patients found an association with response.<br>OR (95% CI): 0.42 (0.20-0.87).<br>P= 0.015[6]<br><br>No association<br>No studies	Association<br>One study with 144 patients found an association with response.<br>OR (95% CI): 2.16 (1.14-4.08)<br>P= 0.014[4]<br><br>No association<br>No studies		
IL12B<br>(rs3213094) C/T	chr5:159323761	Association<br>No studies<br><br>No association<br>One study with 121 patients found no association with response. P=0.35[8]	Association<br>One study with 79 patients found an association with response.<br>OR (95% CI):<br>1.95 (1.31–2.92)<br>P=1.41E-02[17]<br><br>No association<br>No studies	Association<br>No studies<br><br>No association<br>One study with 159 patients found no association with response. P=0.31[8]		Association<br>One study with 66 patients found an association with response. P=0.017[8]<br><br>No association<br>No studies	
IL12B<br>(rs6887695) G/C	chr5:159395637		Association<br>No studies<br><br>No association<br>One study with 79 patients found no association with response. OR (95% CI): 1.65 CI:1.09–2.51. P= 0.24[17]		Association<br>One study with 144 patients found an association with response. OR (95% CI): 2.60 (1.19-5.66). P= 0.014[4]<br><br>No association<br>One study with 376 patients found no association with response.	Association<br>No studies<br><br>No association<br>Two studies found no association with response.<br><br>Galluzzo et al: n=64 OR: 1.21 P=0.756[18]<br><br>Loft et al:<br>n=230	

					OR (95% CI): 0.83 (0.42-1.65), P=0.59[10]	OR (95% CI): 1.17 (0.48-2.89), P=0.73[10]	
IL17A (rs2275913) G/A	chr6:52186235	Association<br>No studies<br><br>No association<br>One study with 67 patients found no association with response.<br>OR (95% CI): 0.63 (0.28-1.42).<br>P=0.250[16]	Association<br>No studies<br><br>No association<br>One study with 36 patients found no association with response.<br>OR (95% CI): 2.78 (0.16-49.22)<br>P= 0.490[16]	Association<br>No studies<br><br>No association<br>One study with 77 patients found no association with response.<br>OR (95% CI): 1.20 (0.41-3.48)<br>P= 0.730[16]	Association<br>No studies<br><br>No association<br>Two studies found no association with response.<br><br>Loft et al: n=376. OR (95% CI): 0.95 (0.46-1.97). P=0.89[10]<br><br>Prieto-Pérez et al: n=194. OR (95% CI): 1.65 (0.38-7.26) P= 0.520[16]	Association<br>One study with 230 patients found an association with response.<br>OR (95% CI): 0.34 (0.12–0.98).<br>P=0.046[10]<br><br>No association<br>One study with 70 patients found no association with response. OR (95% CI): 2.98 (0.84-10.63).<br>P=0.077[16]	Association<br>No studies<br><br>No association<br>One study found no association with response.<br><br>van Vugt et al: n=132 P= 0.5424[19]
IL17RA (rs4819554) G/A	chr22:17084145				Association<br>One study with 238 patients found an association with response.<br>OR (95% CI): 1.86 (1.05-3.27). P=0.03[20]<br><br>No association<br>No studies		
IL18 (rs187238) C/G	chr11:112164265				Association<br>One study with 376 patients found an association with response.<br>OR (95% CI): 0.47 (0.23–0.95).<br>P=0.036[10]	Association<br>No studies<br><br>No association<br>One study with 230 patients found no association with response.	

					No association<br>No studies	OR (95% CI): 0.55 (0.23-1.36), P=0.19[10]	
IL19 (rs2243188) C/A	chr1:206841127			Association<br>One study with 78 patients found an association with response.<br>OR (95% CI): 3.01 (1.03–08.75) P= 0.048[6]<br><br>No association<br>No studies	Association<br>One study with 144 patients found an association with response.<br>OR (95% CI): 2.18 (1.02-4.70) P= 0.007[4]<br><br>No association<br>No studies		
IL1B (rs1143623) C/G	chr2:112838252				Association<br>One study with 376 patients found an association with response. P=0.0041[10]<br><br>No association<br>No studies	Association<br>One study with 230 patients found an association with response. P=0.0049[10]<br><br>No association<br>No studies	
IL1B (rs1143627) T/C	chr2:112836810				Association<br>One study with 376 patients found an association with response. P=0.0016[10]<br><br>No association<br>No studies	Association<br>One study with 230 patients found an association with response. P=0.0045[10]<br><br>No association<br>No studies	
IL1RN (rs397211) T/C	chr2:113134564				Association<br>Two studies found an association with response.<br><br>Prieto-Pérez et al:		

					n=144. OR (95% CI): 0.00 (0.00-ND) P=0.015[4]<br><br>Ovejero-Benito et al: n= OR (95% CI): 0.00 (0.00-ND). P=0.021[3]<br><br>No association<br>No studies		
IL1RN (rs4251961) T/C	chr2:113116890				Association<br>No studies<br><br>No association<br>One study with 376 patients found no association with response.<br>OR (95% CI): 0.81 (0.40-1.62). P=0.55[10]	Association<br>One study with 230 patients found an association with response.<br>OR (95% CI): 2.31 (1.01–5.29). P=0.048[10]<br><br>No association<br>No studies	
IL23A (rs2066808) C/T	chr12:56344189		Association<br>One study with 79 patients found an association with response.<br>OR (95% CI): 10.7 (1.69–444.64). P=4.14E-02[17]<br><br>No association<br>No studies				
IL23R (rs11209026) G/A	chr1:67240275	Association<br>No studies		Association<br>No studies<br>No association<br>Two studies found	Association<br>One study with 109 patients found	Association<br>No studies	

		No association<br>One study with 121 patients found no association with response. P=0.80[8]		no association with response.<br>van den Reek et al: n=161. P=0.71[8]<br><br>Gallo et al: n=61 P= P=0.420[21]	association with response. P=0.049[21]<br><br>No association<br>One study with 376 patients found no association with response.<br>OR (95% CI): 0.74 (0.21-2.62), P=0.65[10]	No association<br>Two studies found no association with response.<br><br>Van den Reek et al: n=66. P=0.68[8]<br><br>Loft et al: n=230. OR (95% CI): 1.06 (0.21-5.40), P=0.94[10]	
IL23R (rs12131065) A/G	chr1:67303323		Association<br>One study with 79 patients found an association with response.<br>OR (95% CI): 2.29 (1.49–3.52). P=1.43E-03[17]<br><br>No association<br>No studies				
IL23R (rs1343152) A/C	chr1:67238649				Association<br>One study with 144 patients found an association with response.<br>OR (95% CI): 0.18 (0.02-1.42). P=0.042[4]<br>No association<br>No studies		
IL23R (rs2201841) A/G	chr1:67228519				Association<br>One study with 144 patients found an association with response.		

					OR (95% CI): 2.98 (1.11-8.03) P=0.034[4]<br><br>No association<br>No studies		
IRAK-3 (rs11541076) A/T	chr12:66254548				Association<br>No studies<br><br>No association<br>One study with 376 patients found no association with response.<br>OR (95% CI): 0.53 (0.26-1.10) P=0.09[10]	Association<br>One study with 230 patients found an association with response.<br>OR (95% CI): 0.38 (0.15–0.96). P=0.042[10]<br><br>No association<br>No studies	
IVL (rs6661932) T/C	chr1:152924258			Association<br>One study with 68 patients found an association with response. OR (95% CI): 0.41 (0.17–11.02). P=0.045[6]<br><br>No association<br>No studies	Association<br>One study with 95 patients found an association with response. OR (95% CI): 2.15 (1.09-4.21). P=0.022[3]<br><br>No association<br>No studies		
KCNIP1 (rs4867965) A/C	chr5:170450803				Association<br>One study with 65 patients found an association with response.<br>OR: 8.148 P=3.45E-05[2]<br><br>No association<br>No studies		
LCE (rs4112788) A/G	chr1:152578800				Association<br>One study with 144		

					patients found an association with response.<br>OR (95% CI): 2.15 (1.00-4.66). P=0.047[4]<br><br>No association<br>No studies		
LINC01185 (rs842636) G/A	chr2:60864815			Association<br>One study with 78 patients found an association with response. OR (95% CI): 4.94 (1.02–23.87). P= 0.032[6]<br><br>No association<br>No studies			
LMO4 (rs983332) G/T	chr1:87666697			Association<br>One study with 68 patients found an association with response. OR (95% CI): NA (0.00–NA). P= 0.026[6]<br><br>No association<br>No studies			
LOC728724 (rs7820834) T/C	chr8:129238197				Association<br>One study with 65 patients found an association with response. OR: 7.857. P= 2.63E-05[2]		

					No association<br>One study with 376 patients found no association with response. OR (95% CI):<br>1.38 (0.60-3.16), P=0.45[10]		
LY96 (rs11465996) C/G	chr8:73989727				Association<br>One study with 376 patients found an association with response.<br>OR (95% CI): 0.33 (0.15-0.71), P=0.0044[10]<br><br>No association<br>No studies	Association<br>No studies<br><br>No association<br>One study with 230 patients found no association with response.<br>OR (95% CI): 0.72 (0.29-1.78), P=0.48[10]	
MACC1 (rs2390256) C/T	chr7:20138259				Association<br>One study with 65 patients found an association with response.<br>OR: 10. P= 1.23E-05[2]<br><br>No association<br>No studies		
MAFB (rs6028945) G/T	chr20:40192165				Association<br>One study with 144 patients found an association with response.<br>OR (95% CI): 0.29 (0.09-0.87) P= 0.014[4]		

					No association<br>No studies		
MAP3K1 (rs96844) A/G	chr5:56900777			Association<br>One study with 78 patients found an association with response. OR (95% CI): 0.33 (0.12–10.91).<br>P= 0.002[6]<br><br>No association<br>No studies	Association<br>Two studies found an association with response.<br><br>Prieto-Pérez et al: n=144. OR (95% CI): 0.18 (0.05-0.67). P= 0.004[4]<br><br>Ovejero-Benito et al: n=90. OR (95% CI): 0.41 (0.21-0.83).P= 0.008[3]<br><br>No association<br>No studies		
MCP1 (rs1024611) A/G	chr17:34252769				Association<br>One study with 144 patients found an association with response.<br>OR (95% CI): 0.28 (0.09-0.83).<br>P=0.008)[4]<br><br>No association<br>No studies		
MICB-DT (rs9267325) G/C	chr6:31493715						Association<br>One study with 62 patients found an association with response. OR (95% CI): 0.19 (0.05 - 0.74)<br>P= 0.0176[11]

							No association<br>No studies
MYD88 (rs7744) A/G	chr3:38142530				Association<br>One study with 144 patients found an association with response. OR (95% CI): 9.00 (0.91-89.35) P=0.039[4]<br><br>No association<br>One study with 376 patients found no association with response.<br>OR (95% CI): 1.38 (0.60-3.16), P=0.45[10]	Association<br>No studies<br><br>No association<br>One study with 230 patients found no association with response. OR (95% CI): 0.79 (0.29-2.07), P=0.63[10]	
NAT2 (rs1799929) C/T	chr8:18400484				Association<br>One study with 144 patients found an association with response.<br>OR (95% CI): 0.17 (0.02-1.33). P=0.033[4]<br><br>No association<br>No studies		
NAT2 (rs1801280) T/C	chr8:18400344				Association<br>One study with 144 patients found an association with response.<br>OR (95% CI): 0.16 (0.02-1.25). P=0.026[4]		

					No association<br>No studies		
NFKBIA (rs2145623) G/C	chr14:35370030				Association<br>One study with 95 patients found an association with response.<br>OR (95% CI): 1.98 (1.04-3.75). P=0.034[3]<br><br>No association<br>No studies	Association<br>One study with 69 patients found an association with response.<br>P=0.001[1]<br><br>No association<br>No studies	
NFKBIA (rs696) C/T	chr14:35401887			Association<br>No studies<br><br>No association<br>One study with 96 patients found no association with response. P < 0.05[22]	Association<br>One study with 376 patients found an association with response. OR (95% CI): 2.42 (1.18–4.94). P=0.015[10]<br><br>No association<br>No studies	Association<br>No studies<br><br>No association<br>One study with 230 patients found no association with response.<br>OR (95% CI): 1.35 (0.55-3.31), P=0.51[10]	
NFKBIB (rs9403) C/G	chr19:38915527				Association<br>One study with 144 patients found an association with response. OR (95% CI): 0.34 (0.13-0.92) P= 0.030[4]<br><br>No association<br>No studies		
NOS2 (rs4795067) A/G	chr17:27779649				Association<br>One study with 144 patients found an association with		

					response.<br>P= 0.013[4]<br><br>No association<br>No studies		
NPFFR2 (rs13139992) G/A	chr4:72176063				Association<br>One study with 243 patients found an association with response.<br>P= 6.33×10^{-8} [5]<br><br>No association<br>No studies		
NPFFR2 (rs77656238) C/T	chr4:72168544				Association<br>One study with 243 patients found an association with response.<br>P= 8.82×10^{-8} [5]<br><br>No association<br>No studies		
PDE6A (rs10515637) A/G	chr5:149862038				Association<br>One study with 65 patients found an association with response.<br>OR: 13.75. P= 1.59E-05[2]<br>No association<br>No studies		
PGLYRP3-19 (rs821421) T/G	chr1:153311047				Association<br>One study with 144 patients found an association with response. OR (95%		

					CI): 2.49 (1.12-5.55). P= 0.027[4]<br><br>No association<br>No studies		
PGLYRP4-07 (rs3006452) C/G	chr1:153344894				Association<br>One study with 144 patients found an association with response.<br>OR (95% CI): 2.49 (1.12-5.55) P= 0.027[4]<br><br>No association<br>No studies		
PGLYRP4-16 (rs3006448) C/A	chr1:153341677				Association<br>One study with 144 patients found an association with response.<br>OR (95% CI): 2.49 (1.12-5.55) P= 0.027[4]<br><br>No association<br>No studies		
PGLYRP4-24 (rs2916205) C/T	chr1:153348279				Association<br>One study with 144 patients found an association with response.<br>OR (95% CI): 2.49 (1.12-5.55) P= 0.027[4]<br><br>No association<br>No studies		

PGLYRP4-30 (rs3006457) T/C	chr1:153347858				Association<br>One study with 144 patients found an association with response.<br>OR (95% CI): 2.46 (1.10-5.49) P= 0.030[4]<br><br>No association<br>No studies		
PSTP1P1 (rs2254441) G/A	chr15:76995677			Association<br>One study with 78 patients found an association with response.<br>OR (95% CI): 0.19 (0.02–1.50). P= 0.048[6]<br><br>No association<br>No studies	Association<br>One study with 144 patients found an association with response.<br>OR (95% CI): 0.14 (0.02-1.11) P= 0.016[4]<br><br>No association<br>No studies		
PTGS2 (rs2206593) A/G	chr1:186673297			Association<br>One study with 78 patients found an association with response.<br>P= 0.025[6]<br><br>No association<br>No studies	Association<br>One study with 144 patients found an association with response.<br>OR (95% CI): 0.16 (0.02-1.21)<br>P= 0.020[4]<br><br>No association<br>No studies		
PTPN22 (rs2476601) A/G	chr1:113834946				Association<br>One study with 144 patients found an association with	Association<br>No studies<br><br>No association<br>One study with 230	

					response.<br>P=0.0098[4]<br><br>No association<br>One study with 376 patients found no association with response.<br>OR (95% CI): 2.01 (0.76-5.29), P=0.16[10]	patients found no association with response.<br>OR (95% CI): 0.86 (0.31-2.39), P=0.77[10]	
PTTG1 (rs2431697) T/C	chr5:160452971			Association<br>One study with 78 patients found an association with non-response.<br>OR (95% CI): 2.72 (1.27–25.86).<br>P= 0.007[6]<br><br>No association<br>No studies	Association<br>Two studies found association with response:<br><br>MOvejero-Benito et al:<br>n=95. OR (95% CI): 2.02 (1.17-3.47)<br>P= 0.009[3]<br><br>Prieto-Pérez et al:<br>n=144.<br>P= 0.0098[4]<br><br>No association<br>No studies		
QKI (rs10945919) A/G	chr6:163765645				Association<br>One study with 144 patients found an association with response.<br>OR (95% CI): 2.78 (1.15-6.71) P=0.028[2]<br><br>No association<br>No studies		

REL (rs13017599) G/A	chr2:60937196				Association<br>One study with 144 patients found an association with response.<br>OR (95% CI): 2.05 (0.93-4.50). P=0.05[4]<br><br>No association<br>No studies		
SDC4 (rs1008953) T/C	chr20:45352086			Association<br>One study with 78 patients found an association with response.<br>P=0.025[6]<br><br>No association<br>No studies			
SHOC2 (rs1927159) A/C	chr10:110986977				Association<br>One study with 65 patients found an association with response.<br>OR: 0.0625. P= 1.97E-05[2]<br><br>No association<br>No studies		
SLC12A8 (rs651630) G/A	chr3:125092470				Association<br>One study with 144 patients found an association with response. OR (95% CI): 2.57 (1.05-6.28). P= 0.043[4]<br><br>No association<br>No studies		

SLC22A4 (rs1050152) C/T	chr5:132340627					Association<br>One study with 69 patients found an association with response.<br>OR (95% CI): 0.17 (0.04-0.74). P= 0.029[1]<br><br>No association<br>No studies	
SLC9A8 (rs645544) A/G	chr20:49885069				Association<br>One study with 95 patients found an association with response. OR (95% CI): 4.30 (1.11-16.65). P=0.033[3]<br><br>No association<br>No studies		
SLCO1C1 (rs3794271) G/A	chr12:20707159	Association<br>No studies<br><br>No association<br>One study with 36 patients found no association with response. P=0.57[23]	Association<br>No studies<br><br>No association<br>One study with 16 patients found no association with response. P=0.14[23]	Association<br>One study with 78 patients found an association with response. P=0.12[23]<br><br>No association<br>No studies			
SPEN (rs6701290) G/A	chr1:15862186				Association<br>One study with 65 patients found an association with response. OR: 9.569. P= 7.11E-06[2]<br><br>No association<br>No studies		

SPRR2F (rs1500941) A/G	chr1:153115185				Association<br>One study with 90 patients found an association with response.<br>OR (95% CI): 0.33 (0.11-0.97). P=0.045[3]<br><br>No association<br>No studies		
STAT4 (rs7574865) T/G	chr2:191099907					Association<br>One study with 69 patients found an association with response. OR (95% CI): 0.24 (0.05-1.18) P= 0.049[1]<br><br>No association<br>No studies	
SUPT3H (rs9472377) A/G	chr6:44834841				Association<br>One study with 243 patients found an association with response.<br>OR (95% CI): 10.61 (3.37–33.48)<br>P= 1.15 × 10−6[5]**<br><br>No association<br>No studies		
TBX21 (rs17250932) T/C	chr17:47731941				Association<br>One study with 376 patients found an association with response.<br>OR (95% CI): 2.39	Association<br>One study with 230 patients found an association with response.<br>OR (95% CI): 0.35	

					(1.06–5.38). P=0.036.[10]<br><br>No association<br>No studies	(0.13–0.93). P=0.034.[10]<br><br>No association<br>No studies	
TIRAP (rs8177374) C/T	chr11:126292948				Association<br>No studies<br><br>No association<br>One study with 376 patients found no association with response. OR (95% CI): 1.36 (0.62-3.01). P=0.44[10]	Association<br>One study with 230 patients found an association with response. OR (95% CI): 8.62 (1.92-38.64) P=0.0051[10]<br><br>No association<br>No studies	
TLR2 (rs11938228) C/A	chr4:153700794				Association<br>One study with 376 patients found an association with response.<br>OR (95% CI): 0.30 (0.14–0.64).<br>P=0.0019[10]<br><br>No association<br>No studies	Association<br>No studies<br><br>No association<br>One study with 230 patients found no association with response. OR (95% CI): 1.69 (0.68-4.21. P=0.26[10]	
TLR2 (rs2289318) G/C	chr4:153712582				Association<br>One study with 144 patients found an association with response.<br>OR (95% CI): 0.20 (0.03-1.53).<br>P= 0.050[4]		

					No association<br>No studies		
TLR2 (rs3804099) T/C	chr4:153703504				Association<br>No studies<br><br>No association<br>One study with 376 patients found no association with response.<br>OR (95% CI): 1.60 (0.77-3.35). P=0.21[10]	Association<br>One study with 230 patients found an association with response.<br>OR (95% CI): 0.24 (0.07–0.88). P=0.031[10]<br><br>No association<br>No studies	
TLR2 (rs4696480) T/A	chr4:153685974				Association<br>One study with 376 patients found an association with response.<br>OR (95% CI): 0.22 (0.08–0.59). P=0.0032[10]<br><br>No association<br>No studies	Association<br>No studies<br><br>No association<br>One study with 230 patients found no association with response.<br>OR (95% CI): 1.99 (0.71-5.54), P=0.19[10]	
TLR4 (rs12377632) T/C	chr9:117710452				Association<br>One study with 376 patients found an association with response.<br>OR (95% CI): 2.06 (1.08–3.94). P =0.029[10]<br><br>No association<br>No studies	Association<br>No studies<br><br>No association<br>One study with 230 patients found no association with response. OR (95% CI): 2.17 (0.82-5.69). P=0.12[10]	

TLR5 (rs5744174) A/G	chr1:223111186				Association<br>No studies<br><br>No association<br>One study with 376 patients found no association with response.<br>OR (95% CI): 0.73 (0.35-1.51). P=0.39[10]	Association<br>One study with 230 patients found an association with response.<br>OR (95% CI): 5.26 (1.93–14.4).<br>P=0.0012[10]<br><br>No association<br>No studies	
TNFα-1031 (rs1799964) T/C	chr6:31574531	Association<br>No studies<br><br>No association<br>One study with 45 patients found no association with response.<br>P=0.694[21]	Association<br>One study with 33 patients found an association with response.<br>P=0.024[21]<br><br>No association<br>No studies	Association<br>No studies<br><br>No association<br>One study with 61 patients found no association with response.<br>P= 0.671[21]	Association<br>One study with 102 patients found an association with response<br>P=0.047[21]<br><br>No association<br>No studies		
TNFα-238 (rs361525) G/A	chr6:31575324	Association<br>No studies<br><br>No association<br>One study with 45 patients found no association with response. P=1.000[21]	Association<br>No studies<br><br>No association<br>One study with 33 patients found no association with response. P=0.233[21]	Association<br>Two studies found an association with response and poor response.<br><br>Gallo et al: n=61. P=0.045[21]<br><br>De Simone et al: n=97. P=0.002[24]<br><br>No association<br>No studies	Association<br>One study with 97 patients found an association with response. P=0.049[21]<br><br>No association<br>One study with 376 patients found no association with response.<br>OR (95% CI): 1.09 (0.47–2.55). P=0.83[10]	Association<br>No studies<br><br>No association<br>One study with 230 patients found no association with response. OR (95% CI): 2.65 (0.91–7.73).<br>P=0.07[10]	
TNFα-308 (rs1800629) G/A	chr6:31575254		Association<br>No studies	Association<br>Two studies found an association with	Association<br>No studies	Association<br>No studies	

			No association<br>One study with 79 patients found no association with response.<br>OR (95% CI): Inf (0.79–Inf). P=0.79[17]	poor response and non-response.<br><br>De Simone et al: n=97. P=0.004[24]<br><br>Hadi et al: n= 100. P<0.0001[25]<br><br>No association<br>No studies	No association<br>Two studies found no association with response.<br><br>Gallo et al: n= 102. P=0.297[21]<br><br>Loft er al: n=376. OR (95% CI): 0.61 (0.29–1.29). P=0.19[10]	No association<br>One study with 230 patients found no association with response.<br>OR (95% CI): 1.66 (0.58–4.71). P=0.34[10]	
TNFα-857 (rs1799724) C/T	chr6:31574705	Association<br>No studies<br><br>No association<br>One study with 45 patients found no association with response.<br>P=0.302[21]	Association<br>No studies<br><br>No association<br>One study with 33 patients found no association with response.<br>P=0.154[21]	Association<br>Two studies found an association with response.<br><br>Gallo et al: n= 62 P=0.041[21]<br><br>Vasilopoulos et al: n=44. P=0.002[26]<br><br>No association<br>One study with 97 patients found no association with response. P=0.05[24]	Association<br>Two studies found an association with response.<br><br>Vasilopoulos et al: n=80. P=0.027[26]<br><br>Gallo et al: n=97. P=0.006[21]<br><br>No association<br>One study with 49 patients found no association with response.<br>P= 0.43[27]		
TNFAIP3 (rs610604) G/T	chr6:137878280	Association<br>No studies<br><br>No association<br>Two studies found no association with	Association<br>No studies<br><br>No association<br>One study with 124 patients found no	Association<br>Three studies found an association with response[12,28] and non-response[29].	Association<br>Two studies found an association with response.<br><br>Ovejero-Benito et al:	Association<br>One study with 66 patients found an association with response. P=0.032[8]	

		response.<br><br>S. Masouri et al:<br>n=40. P=0.16[12]<br><br>J.M.P.A. van den Reek et al:<br>n=121. P=0.66[8]	association with response. P=0.501[12]	Masouri et al:<br>n=35. P=0.007[12]<br><br>Tejasvi et al:<br>n=343. OR = 1.64, P=0.030[28]<br><br>Hadi et al:<br>n=100. P<0.01[29]<br><br>No association<br>One study with 159 patients found no association with response. P=0.43[8]	n=95. OR (95% CI): 0.15 (0.02-1.24)<br>P= 0.027[3]<br><br>Dauden et al:<br>n=20[30]<br><br>No association<br>One study with 49 patients found no association with response. P>0.05[27]	No association<br>One study with 51 patients found no association with response<br>OR: 1.6 P = 0.75[31]	
TNFAIP3 (rs6920220) G/A	chr6:137685367				Association<br>One study with 20 patients found an association with response.[30]<br>P="significant"<br><br>No association<br>No studies		
TNFRSF1A (rs4149570) A/C	chr12:6342424				Association<br>One study with 144 patients found an association with response.<br>OR (95% CI): 2.80 (1.09-7.16). P=0.035[4]<br><br>No association<br>One study with 376 patients found no association with response. OR (95% CI): 0.57 (0.27-1.19),	Association<br>No studies<br><br>No association<br>One study with 230 patients found no association with response. OR (95% CI): 1.63 (0.64-4.13), P=0.30[10]	

					P=0.14[10]		
TNFR1B (rs1061622) T/G	chr1:12192898			Association<br>One study with 44 patients found an association with response.<br>P= 0.0001[26]<br><br>No association<br>One study with 100 patients found no association.<br>P=0.38 [29]	Association<br>Four studies found an association with response (T-allele) or non-response[3,32] (G-allele).<br><br>Vasilopoulos et al: n=80 P= 0.027.[26]<br><br>Prieto-Pérez et al: n=144 OR (95% CI): 2.13 (0.94-4.86) P= 0.038[4]<br><br>González-Lara et al: n=90. OR (95 % CI) = 2.96 (1.09–8.02). P=0.03[32]<br><br>Ovejero-Benito et al: n=90. OR (95 % CI) = 3.76 (1.26-11.22). P= 0.014[3]<br><br>No association<br>One study with 49 patients found no association<br>P > 0.05[27]		
TRAF3IP2 (rs13190932) G/A	chr6:111591867	Association<br>No studies<br><br>No association<br>One study with 124 patients found no	Association<br>One study with 124 patients found an association with response.<br>P=0.041[12]	Association<br>No studies<br><br>No association<br>One study with 124 patients found no			

		association with response. P=0.654[12]	No association<br>No studies	association with response. P=1.00[12]			
TRAF3IP2 (rs240993) T/C	chr6:111352511	Association<br>No studies<br><br>No association<br>One study with 120 patients found no association with response. P=0.43[8]	Association<br>One study with 79 patients found an association with response.<br>OR (95% CI): 2.08 (1.36–3.23) P= 1.00E-02[17]<br><br>No association<br>No studies	Association<br>No studies<br><br>No association<br>One study with 158 patients found no association with response. P=0.31[8]		Association<br>No studies<br><br>No association<br>One study with 65 patients found no association with response. P=0.78[8]	
TSC1 (rs1076160) C/T	chr9:132900647				Association<br>One study with 144 patients found an association with response.<br>OR (95% CI): 0.16 (0.02-1.25) P=0.026[4]<br><br>No association<br>No studies		
TYK2 (rs2304255) C/T	chr19:10364973						Association<br>One study with 62 patients found an association with response.<br>OR (95% CI): 14.6 (1.47-144) P=0.0063[11]<br><br>No association<br>No studies
ZNF816A (rs9304742) T/C	chr19:52948038			Association<br>One study with 78	Association<br>Two studies found an		

				patients found an association with non-response. OR (95% CI): 4.50 (1.07–18.88). P= 0.040[6]<br><br>No association<br>No studies	association with nonresponse and response:<br><br>Prieto-Pérez et al: n=144. OR (95% CI): 2.98 (1.11-8.03). P= 0.034[4]<br><br>Ovejero-Benito et al: n=95. OR (95% CI): 0.32 (0.13-0.80). P=0.013[3]<br><br>No association<br>No studies		
** Genome wide significance. ($p < 5 \times 10^{-8}$) not reached<br>#Different minor allele than National Center of Biotechnology Information in the National Institute of Health (NCBI, NIH) due to use of minor allele in included studies							

References

1. Prieto-Pérez R, Mar, Llamas-Velasco, et al. Pharmacogenetics of ustekinumab in patients with moderate-to-severe plaque psoriasis. *Pharmacogenetics*. 2017;18(2):157-164.
2. Nishikawa R, Nagai H, Bito T, et al. Genetic prediction of the effectiveness of biologics for psoriasis treatment. *J Dermatol*. 2016;43(11):1273-1277. doi:10.1111/1346-8138.13412
3. María Carmen Ovejero-Benito, Rocío Prieto-Pérez, Mar Llamas-Velasco, Ester Muñoz- Aceituno, Alejandra Reolid, Miriam Saiz-Rodríguez, Carmen Belmonte, Manuel Román, Dolores Ochoa, María Talegón, Teresa Cabaleiro, Esteban Daudén FA-S. Polymorphisms associated with adalimumab and infliximab response in moderate- to-severe plaque psoriasis. *Pharmacogenomics*. 2018;19(1):7-16. http://content.ebscohost.com/ContentServer.asp?EbscoContent=dGJyMNLe80Sep7Q4y9f3OLCmr1Gep7JSsKy4Sa6WxWXS&ContentCustomer=dGJyMPGptk%2B3rLJNuePfgeyx43zx1%2B6B&T=P&P=AN&S=R&D=buh&K=134748798%0Ahttp://amg.um.dk/~/media/amg/Documents/Policies and Strategies/S
4. Prieto-Pérez R, Solano-López G, Cabaleiro T, et al. New polymorphisms associated with response to anti-TNF drugs in patients with moderate-to-severe plaque

psoriasis. *Pharmacogenomics J*. 2018;18(1):70-75. doi:10.1038/tpj.2016.64

5. Ovejero-Benito MC, Muñoz-Aceituno E, Sabador D, et al. Genome-wide association analysis of psoriasis patients treated with anti-TNF drugs. *Exp Dermatol*. 2020;29(12):1225-1232. doi:10.1111/exd.14215
6. Ovejero-Benito MC, Prieto-Pérez R, Llamas-Velasco M, et al. Polymorphisms associated with etanercept response in moderate-to-severe plaque psoriasis. *Pharmacogenomics*. 2017;18(7):631-638.
7. Coto-Segura P, González-Fernández D, Batalla A, et al. Common and rare CARD14 gene variants affect the antitumour necrosis factor response among patients with psoriasis. *Br J Dermatol*. 2016;175(1):134-141. doi:10.1111/bjd.14461
8. van den Reek JMPA, Coenen MJH, van de L'Isle Arias M, et al. Polymorphisms in CD84, IL12B and TNFAIP3 are associated with response to biologics in patients with psoriasis. *Br J Dermatol*. 2017;176(5):1288-1296. doi:10.1111/bjd.15005
9. Coto-Segura P, Batalla A, González-Fernández D, et al. CDKAL1 gene variants affect the anti-TNF response among Psoriasis patients. *Int Immunopharmacol*. 2015;29(2):947-949. doi:10.1016/j.intimp.2015.11.008
10. Loft ND, Skov L, Iversen L, et al. Associations between functional polymorphisms and response to biological treatment in Danish patients with psoriasis. *Pharmacogenomics J*. 2018;18(3):494-500. doi:10.1038/tpj.2017.31
11. Morelli M, Galluzzo M, Madonna S, et al. HLA-Cw6 and other HLA-C alleles, as well as MICB-DT, DDX58, and TYK2 genetic variants associate with optimal response to anti-IL-17A treatment in patients with psoriasis. *Expert Opin Biol Ther*. 2021;21(2):259-270. doi:10.1080/14712598.2021.1862082
12. Masouri S, Stefanaki I, Ntritsos G, et al. A Pharmacogenetic Study of Psoriasis Risk Variants in a Greek Population and Prediction of Responses to Anti-TNF-α and Anti-IL-12/23 Agents. *Mol Diagnosis Ther*. 2016;20(3):221-225. doi:10.1007/s40291-016-0198-z
13. Mendrinou E, Patsatsi A, Zafiriou E, et al. FCGR3A-V158F polymorphism is a disease-specific pharmacogenetic marker for the treatment of psoriasis with Fc-containing TNFα inhibitors. *Pharmacogenomics J*. 2017;17(3):237-241. doi:10.1038/tpj.2016.16
14. Batalla A, Coto E, Coto-Segura P. Influence of Fcγ receptor polymorphisms on response to anti-tumor necrosis factor treatment in psoriasis. *JAMA Dermatology*. 2015;151(12):1376-1378. doi:10.1001/jamadermatol.2015.2818
15. Julià M, Guilabert A, Lozano F, et al. The role of Fcγ receptor polymorphisms in the response to anti-tumor necrosis factor therapy in psoriasis: A pharmacogenetic study. *JAMA Dermatology*. 2013;149(9):1033-1039. doi:10.1001/jamadermatol.2013.4632
16. Prieto-Pérez R, Solano-López CT et al. The polymorphism rs763780 in the IL-17F gene is associated with response to biological drugs in patients with psoriasis.

Pharmacogenomics. 2015;16(15):1723-1731.

17. Torii K, Morita A. Specific single nucleotide polymorphism genotypes and association of an IL-12B polymorphism with secondary failure of infliximab therapy in Japanese psoriasis patients. *J Dermatol Sci*. 2020;99(2):135-136. doi:10.1016/j.jdermsci.2020.05.011
18. Galluzzo M, Boca AN, Botti E, et al. IL12B (p40) Gene Polymorphisms Contribute to Ustekinumab Response Prediction in Psoriasis. *Dermatology*. 2016;232(2):230-236. doi:10.1159/000441719
19. van Vugt LJ, van den Reek JMPA, Meulewaeter E, et al. Response to IL-17A inhibitors secukinumab and ixekizumab cannot be explained by genetic variation in the protein-coding and untranslated regions of the IL-17A gene: results from a multicentre study of four European psoriasis cohorts. *J Eur Acad Dermatology Venereol*. 2020;34(1):112-118. doi:10.1111/jdv.15787
20. Batalla A, Coto E, Gómez J, et al. IL17RA gene variants and anti-TNF response among psoriasis patients. *Pharmacogenomics J*. 2018;18(1):76-80. doi:10.1038/tpj.2016.70
21. Gallo E, Cabaleiro T, Román M, et al. The relationship between tumour necrosis factor (TNF)-α promoter and IL12B/IL-23R genes polymorphisms and the efficacy of anti-TNF-α therapy in psoriasis: A case-control study. *Br J Dermatol*. 2013;169(4):819-829. doi:10.1111/bjd.12425
22. Caldarola G, Sgambato A, Fanali C, et al. HLA-Cw6 allele, NFkB1 and NFkBIA polymorphisms play no role in predicting response to etanercept in psoriatic patients. *Pharmacogenet Genomics*. 2016;26(9):423-427. doi:10.1097/FPC.0000000000000233
23. Julià A, Ferrandiz C, Dauden E, et al. Association of the PDE3A-SLCO1C1 locus with the response to anti-TNF agents in psoriasis. *Pharmacogenomics J*. 2015;15(4):322-325. doi:10.1038/tpj.2014.71
24. De Simone C, Farina M, Maiorino A, et al. TNF-alpha gene polymorphisms can help to predict response to etanercept in psoriatic patients. *J Eur Acad Dermatology Venereol*. 2015;29(9):1786-1790. doi:10.1111/jdv.13024
25. Hassan Hadi AM, Abdul-Hassan Abbas A, Abdulamir AS, Fadheel BM. Impact of TNF-α and TNFR1B genes polymorphisms on disease susceptibility and predict response to anti-TNF therapy in psoriatic patients. *Ann Trop Med Public Heal*. 2020;23(14). doi:10.36295/ASRO.2020.231442
26. Vasilopoulos Y, Manolika M, Zafiriou E, et al. Pharmacogenetic analysis of TNF, TNFRSF1A, and TNFRSF1B gene polymorphisms and prediction of response to anti-TNF therapy in psoriasis patients in the greek population. *Mol Diagnosis Ther*. 2012;16(1):29-34. doi:10.2165/11594660
27. Ito M, Hirota T, Momose M, et al. Lack of association of TNFA, TNFRSF1B and TNFAIP3 gene polymorphisms with response to anti-tumor necrosis factor therapy in Japanese patients with psoriasis. *J Dermatol*. 2020;47(4):e110-e111. doi:10.1111/1346-8138.15200

28. Tejasvi, T.; Pstuart, P.E.; Nair, R.P.; Voorhees, J.J.; Elder JT. Polymorphisms in TNFAIP3 as a predictor of response to TNF blockade in psoriasis. *J Invest Dermatol*. 2009;129(0):55.

29. Hassan Hadi AM, Abbas AA-H, Abdulamir AS, Fadheel BM. The effect of TnFaip3 gene polymorphism on disease susceptibility and response of etanercept in psoriatic patients. *Eur J Mol Clin Med*. 2020;7(9):240-246.

30. Dauden, E.; Munoz-aceituno, E.; Reolid, A.; Llamas-velasco, M.; Ovejero-benito, M.C.; Hevia, L.; Prieto-perez, R.; Abad-santos F. Polymorphisms associated with anti-TNF response in patients with psoriasis and psoriatic arthritis. *J Dermatol Nurses Assoc*. 2020;12(2).

31. Talamonti M, Botti E, Galluzzo M, et al. Pharmacogenetics of psoriasis: HLA-Cw6 but not LCE3B/3C deletion nor TNFAIP3 polymorphism predisposes to clinical response to interleukin 12/23 blocker ustekinumab. *Br J Dermatol*. 2013;169(2):458-463. doi:10.1111/bjd.12331

32. González-Lara L, Batalla A, Coto E, et al. The TNFRSF1B rs1061622 polymorphism (p.M196R) is associated with biological drug outcome in Psoriasis patients. *Arch Dermatol Res*. 2015;307(5):405-412. doi:10.1007/s00403-014-1533-z
