# Supplementary material for: The Association between Genetics and Response to Treatment with Biologics in Patients with Psoriasis, Psoriatic Arthritis, Rheumatoid Arthritis, and Inflammatory Bowel Diseases: A Systematic Review and Meta-Analysis
Source: Int J Mol Sci. 2024 May 26;25(11):5793. doi: 10.3390/ijms25115793 (PMC11171831; doi:10.3390/ijms25115793)
Supplement: Supplementary file 1 [file ijms-25-05793-s001.zip › Supplementary Table S3.pdf]

PsA Supplementary Table S3

		Anti-TNF			
Gene<br>(rs number)	Chromosome number:location	Adalimumab	Infliximab	Etanercept	Anti-TNF overall
FCGR2A<br>rs1801274<br>G/A#	chr1:161509955			Association<br>One study with 55 patients found an association with response.<br>P=0.034.[1]<br><br>No association<br>No studies	Association<br>Two studies found an association with response.<br><br>Ramírez et al:<br>n=103. P=0.030.[1]<br><br>Morales-Lara et al:<br>n=11.[2]<br><br>No association<br>No studies
FCGR3A<br>rs396991<br>A/C	chr1:161544752		Association<br>One study with 16 patients found an association with response.<br>P=0.036[2]<br><br>No association<br>No studies	Association<br>No studies<br><br>No association<br>One study with 55 patients found no association with response.<br>P=0.705.[1]	Association<br>No studies<br><br>No association<br>One study with 103 patients found no association with response. P=1.000.[1]
SLCO1C1<br>rs3794271<br>A/G	chr12:20707159				Association<br>One study with 81 patients found an association with response.<br>P=0.0036[3]<br><br>No association<br>No studies

TNF+489<br>(rs80267959)<br>A/G	chr6:31576050			Association<br>One study with 37 patients found an association with response. P=0.021.[4]<br><br>No association<br>No studies	Association<br>No studies<br><br>No association<br>One study with 57 patients found no association with response.[4]
TNFAIP3<br>(rs610604)<br>G/T	chr6:137878280				Association<br>One study with 20 patients found an association with response.[5]<br><br>No association<br>No studies
TNFAIP3<br>(rs6920220)<br>G/A	chr6:137685367				Association<br>One study with 20 patients found an association with response.[5]<br><br>No association<br>No studies
TNFR1A<br>(rs767455)<br>A/G	chr12:6341779				Association<br>One study with 55 patients found an association with response. P=0.042[6]<br><br>No association<br>No studies
TNFRSF1B<br>rs1061624<br>A/G	chr1:12207208				Association<br>One study with 20 patients found an association with response.[5]<br><br>No association<br>No studies

TRAILR1 (rs20575) C/G	chr8:23201811		Association<br>One study with 27 patients found an association with response. P=0.048[6]<br><br>No association<br>No studies		
#Different minor allele than National Center of Biotechnology Information in the National Institute of Health (NCBI, NIH) due to use of minor allele in included studies					

References

1. Ramírez J, Fernández-Sueiro JL, López-Mejías R, et al. FCGR2A/CD32A and FCGR3A/CD16A variants and EULAR response to tumor necrosis factor-α blockers in psoriatic arthritis: A longitudinal study with 6 months of followup. *J Rheumatol*. 2012;39(5):1035-1041. doi:10.3899/jrheum.110980

2. Morales-Lara MJ, Conesa-Zamora P, Garca-Simón MS, et al. Association between the FCGR3A V158F polymorphism and the clinical response to infliximab in rheumatoid arthritis and spondyloarthritis patients. *Scand J Rheumatol*. 2010;39(6):518-520. doi:10.3109/03009741003781969

3. Antonio Julià, Jesús Rodríguez, José Luis Fernández-Sueiro, Jordi Gratacós, Rubén Queiró, Carlos Montilla, Juan Carlos Torre-Alonso, José Javier Pérez-Venegas, Sara Manrique-Arija, Santiago Muñoz-Fernández, Carlos González, Daniel Roig, Pedro Zarco, Alba JD, Marsal* C& S. PDE3A-SLCO1C1 locus is associated with response to anti-tumor necrosis factor therapy in psoriatic arthritis. *Pharmacogenomics*. 2014;15(4):1763-1769.

4. Murdaca G, Gulli R, Spanò F, et al. TNF-α gene polymorphisms: Association with disease susceptibility and response to anti-TNF-α treatment in psoriatic arthritis. *J Invest Dermatol*. 2014;134(10):2503-2509. doi:10.1038/jid.2014.123

5. M.C. OB, E. MA, A. R, et al. Polymorphisms associated with anti-TNF response in psoriatic arthritis. *Basic Clin Pharmacol Toxicol*. 2018;123:89.

6. Morales-Lara MJ, Cañete JD, Torres-Moreno D, et al. Effects of polymorphisms in TRAILR1 and TNFR1A on the response to anti-TNF therapies in patients with rheumatoid and psoriatic arthritis. *Jt Bone Spine*. 2012;79(6):591-596. doi:10.1016/j.jbspin.2012.02.003
