# Supplementary material for: The Association between Genetics and Response to Treatment with Biologics in Patients with Psoriasis, Psoriatic Arthritis, Rheumatoid Arthritis, and Inflammatory Bowel Diseases: A Systematic Review and Meta-Analysis
Source: Int J Mol Sci. 2024 May 26;25(11):5793. doi: 10.3390/ijms25115793 (PMC11171831; doi:10.3390/ijms25115793)
Supplement: Supplementary file 1 [file ijms-25-05793-s001.zip › Supplementary Table S4.pdf]

RA Supplementary Table S4

		Anti-TNF				CD20-antibody	Anti-IL-6	Anti-IL-1R	T-cell costimulation blocker
Gene (rs number)	Chromosome number:location	Adalimumab	Infliximab	Etanercept	Anti-TNF-overall*	Rituximab	Tocilizumab	Unspecific	Abatacept
AFF3 (rs10865035) A/G	chr2:100219272				Association: One study with 1334 patients found an association with response. P=0.013[1]<br><br>No association: One study with 183 patients found no association with response. OR (95% CI): 1.26 (0.72-2.18). P=0.29[2]				
AFF3 (rs1160542) G/A	chr2:100215693				Association: One study with 1334 patients found an association with response. P= 0.013.[1]<br><br>No association: No studies				
ALPL (rs869179) G/A	chr1:21513636				Association: One study with 196 patients found an association with response. P= 1.81E–05.[3]				

					No association:<br>No studies				
ALPL<br>(rs885813)<br>C/T	chr1:21550581				Association:<br>One study with 196 patients found an association with response.<br>P= 9.99E–06.[3]<br><br>No association:<br>One study with 566 patients found no association with non-response.<br>OR (95%): 0.9 (0.5-1.5). P=0.6.[4]				
ALPL<br>(rs885814)<br>C/T	chr1:21549423				Association:<br>One study with 196 patients found an association with response.<br>P= 5.52E-06.[3]<br><br>No association:<br>One study with 566 patients found no association with non-response. OR (95%): 1.4 (1.0-2.1). P=0.07.[4]				
ARHGAP44<br>(rs2072255)<br>G/A	chr17:12949075			Association:<br>One study with 134 patients found an association with secondary failure.					

				OR= 4.189<br>P= 2.42 x 10^{-5}.[5]<br><br>No association:<br>No studies					
ARMC2<br>(rs6941263)<br>T/A	chr6:108840891				Association:<br>One study with 361 patients found an association with response.<br>OR (95% CI):<br>0.2 (0.11-0.37).<br>P=6.22E-0.8.[6]<br><br>No association:<br>No studies				
BRINP1/LINC0161 3<br>(rs10739537)<br>G/T	chr9:119426326	Association:<br>One study with 551 patients found an association with response.<br>P=9.11E-08. [7]**<br><br>No association:<br>No studies							
C10orf90/DOCK1<br>(rs11599217)<br>G/T	chr10:126878395		Association:<br>One study with 434 patients found an association with response.<br>P =7.27E-08. [7]**<br><br>No association:<br>No studies						
C9orf47<br>(rs11525966)<br>T/C	chr9:88947021				Association:<br>One study with 196 patients found an association				

					with response.<br>P = 1.90E–05[3]<br><br>No association:<br>No studies				
C9orf47<br>(rs1875620)<br>G/A	chr9:88925144				Association:<br>One study with 196 patients found an association with response.<br>P = 1.90E–05[3]<br><br>No association:<br>No studies				
C9orf72<br>(rs774359)<br>T/C	chr9:27561051				Association:<br>Two studies found and association with response<br><br>Krintel et al:<br>n=196<br>OR (95% CI):<br>1.87 (1.1 - 3.3).<br>P=0.03.[3]***<br><br>Liu et al:<br>n=89<br>OR (95% CI):<br>5.4 (1.9 - 17.3).<br>P=0.0005.[8]<br><br>No association:<br>Two studies found no association with response:<br>Suarez-Gestal et al: n= 151<br>OR (95% CI): 1.64 (0.8-3.3). P=0.1[9]				

					Mirkov et al: n=882. P=0.89[10]				
C9orf72 (rs3849942) C/T	chr9:27543283				Association: Two studies found and association with response<br><br>Krintel et al: n=196 OR (95% CI): 1.85 (1.0 - 3.3). P=0.03.[3]***<br><br>Liu et al: n=89 OR (95% CI): 5.0 (1.7 - 15.8) P=0.001.[8]<br><br>No association: Two studies found no association with non-response/respons e:<br><br>Suarez-Gestal et al: n= 151 OR (95% CI): 1.41 (0.7-2.5).P=0.3[9]<br><br>Mirkov et al: n=882. P=0.98[10]				
CARD8 (rs10403848) G/A	chr19:48253518				Association: One study with 1278 patients found an association with response.				

					P=0.020[11]<br><br>No association:<br>One study with 566 patients found no association with response. RR (95% CI) = 1.09 (0.97 to 1.23). P=0.15[4]				
CARD8 (rs11672725) C/T	chr19:48243424				Association:<br>One study with 1278 patients found an association with response.<br>RR (95% CI): 1.05 (1.00 to 1.11). P=0.032.[11]<br><br>No association:<br>One study with 566 patients found no association with response. P=0.7.[4]				
CBLN2 (rs1539909) T/C	chr18:71581359				Association:<br>One study with 196 patients found an association with response. P= 4.13E –06.[3]<br><br>No association:<br>No studies				
CCR5 (rs2734648) G/T	chr3:46370349				Association:<br>One study with 281 patients found				

					an association with response.<br>OR (95% CI): 2.06 (1.02–4.13).<br>P=0.04[12]<br><br>No association:<br>No studies				
CD226 (rs763361) T/C	chr18:69864406				Association:<br>One study with 1334 patients found an association with response P=0.048[1]<br><br>No association:<br>One study with 173 patients found no association with response.<br>OR (95% CI): 1.015 (0.58-1.76).<br>P=1[2]				
CD40LG (rs1126535) T/C	chrX:136648396	Association:<br>One study with 291 patients found an association with response.<br>OR (95% CI): 1.871 (1.190–2.942).<br>P=0.0066[13]<br><br>No association:<br>No studies							
CD58 (rs11586238) C/G	chr1:116720516				Association:<br>One study with 1283 patients				

					found an association with response. OR: 1.31. P=0.04.[14]<br><br>No association:<br>No studies				
CD69<br>(7)<br>A/G	chr12:9753094						Association:<br>Two studies found an association with response<br><br>Maldonado-Montoro et al:<br>n=79<br>P=0.013[15]<br><br>Wang et al:<br>n=1091<br>OR: 0.56.<br>P=0.0039[16]<br><br>No association:<br>One study with 184 patients found no association with response:<br>P = 0.17[17]		
CD69<br>(rs10844706)<br>C/A	chr12:9757536	Association:<br>One study with 291 patients found an association with response.<br>OR (95% CI):<br>1.6(1.058–2.419).<br>P=0.026[13]							

		No association:<br>No studies							
CD94<br>(rs2302489)<br>A/T	chr12:10307944				Association:<br>One study with 225 patients found an association with response.<br>OR (95% CI): 0.30 (0.08–0.88)<br>P=0.017[18]<br><br>No association:<br>No studies				
CENTD1<br>(rs437943)<br>T/C	chr4:35370476		Association:<br>No studies<br><br>No association:<br>One study with 101 patients found no association with non-response.<br>OR (95% CI): 1.74 (0.8-4.0), P=0.19[9]		Association:<br>One study with 89 patients found an association with non-response.<br>OR (95% CI): 4.6 (1.8 - 12.3).<br>P= 0.0007[8]<br><br>No association:<br>Three studies found no association with non-response.<br><br>Krintel et al:<br>n = 196<br>OR (95% CI): 1.15 (0.7 - 1.9). P=0.61[3]<br><br>Suarez-Gestal et al:<br>n=151<br>OR (95% CI): 0.53 (0.3-1.0), P=0.06[9]				

					Mirkov et al:<br>n=882. P=0.93[10]				
CHUK<br>(rs11591741)<br>G/C	chr10:100216744		Association:<br>No studies<br><br>No association:<br>One study with 400 patients found no association with response.<br>P=0.328[19]	Association:<br>One study with 386 found an association with response.<br>P=0.008[19]<br><br>No association:<br>No studies	Association:<br>Two studies found an association with response.<br><br>Ferreiro- Iglesias et al:<br>n=755. P=0.041.[20]<br><br>Potter er al:<br>n=883.<br>OR (95%CI): 0.77 (0.61 to 0.99).<br>P=0.042[19]<br><br>No association:<br>Two studies found no association with response.<br><br>Sode et al:<br>n= 971<br>OR (95% CI): 0.90(0.73-1.0)<br>P=0.31[21]<br><br>Zervou et al:<br>n= 173<br>OR (95% CI): 1.27 (0.71-2.26).<br>P=0.32[2]				
CHUK<br>(rs11595324)<br>T/C	chr10:100219661		Association:<br>No studies<br><br>No association:<br>One study with 400 patients	Association:<br>No studies<br><br>No association:<br>One study with 386 patients	Association:<br>One study with 909 patients found an association with response.<br>P= 0.023[19]				

			found no association with response. P>0.05[19]	found no association with response. P>0.05[19]	No association:<br>No studies				
CHUK (rs2230804) C/T	chr10:100218126			Association:<br>One study with 376 patients found an association with response. P=0.041[19]<br><br>No association:<br>No studies	Association:<br>One study with 896 patients found an association with response. P= 0.037[19]<br><br>No association:<br>No studies				
CLEC2D (rs1560011) A/G	chr12:9670356						Association:<br>One study with 1091 patients found an association with response. OR=0.72. P=0.046.[16]<br><br>No association:<br>One study with 79 patients found no association with response. P=0.058.[15]		
CNTN5 (rs1813443) G/C	chr11:100140279				Association:<br>One study with 1821 patients found an association with response. P = 1.37×10^{-4}.[10]<br><br>No association:<br>No studies				

CNTNAP4 (rs17679567) T/C	chr16:76856229				Association:<br>One study with 196 patients found an association with response.<br>P= 9.04E–06.[3]<br><br>No association:<br>No studies				
CNTNAP5 (rs1827596) T/G	chr2:124498866				Association:<br>One study with 196 patients found an association with response.<br>P= 3.04E–05.[3]<br><br>No association:<br>No studies				
CREBBP (rs8046065) C/T	chr16:3788297				Association:<br>One study with 196 patients found an association with response.<br>P = 1.43E –05. [3]<br><br>No association:<br>No studies				
CSF1R (rs11749913) G/C	chr5:150103864	Association:<br>One study with 291 patients found an association with response.<br>OR (95% CI): 1.5 (1.004–2.243).<br>P=0.048[13]<br><br>No association:<br>No studies							

CST5 (rs6138150) T/C	chr20:23866372				Association:<br>Two studies found and association with response<br><br>Krintel et al:<br>n=196<br>OR (95% CI):<br>0.44 (0.2 - 0.9)<br>P=0.04.[3]***<br><br>Liu et al:<br>n=89<br>OR (95% CI):<br>11.1 (2.5 - 103.3)<br>P=0.0002.[8]<br><br>No association:<br>Two studies found no association with response:<br>Suarez-Gestal et al: n= 151<br>OR (95% CI): 0.94 (0.4-2.2). P=0.9[9]<br><br>Mirkov et al:<br>n=882. P=0.66[10]				
CTLA4 (rs231775) A/G	chr2:203867991				Association:<br>No studies<br><br>No association:<br>One study with 109 patients found no association with response.<br>OR (95% CI): 3.48 (1.20–10.09).<br>P=0.022[22]				

CTLA4<br>(rs3087243)<br>G/A	chr2:203874196				Association:<br>No studies<br><br>No association:<br>One study with 1012 patients found no association with response.<br>P=0.427[1]	Association:<br>No studies<br><br>No association:<br>One study with 53 patients found no association with response.[23]			Association:<br>One study with 92 patients found an association with response.<br>OR (95 CI): 4.41 (1.56-14-59).<br>P=0.005[22]<br><br>No association:<br>No studies
CTLA4<br>(rs5742909)<br>C/T	chr2:203867624								Association:<br>One study with 109 patients found an association with response.<br>OR (95% CI): 5.88 (1.48-23.29)<br>P=0.012[22]<br><br>No association:<br>No studies
CYP1A1<br>(rs1799814)<br>G/T	chr15:74720646				Association:<br>One study with 548 patients found an association with response.<br>OR (95% CI): 1.66 (1.19–2.34).<br>P=0.003[24]<br><br>No association:<br>No studies				
CYP1B1<br>(rs1056836)<br>G/C	chr2:38071060				Association:<br>One study with 548 patients found an association with response.				

					OR (95% CI): 1.34 (1.03–1.77). P=0.032.[24]<br><br>No association:<br>No studies				
CYP2C9 (rs1799853) C/T	chr10:94942290				Association:<br>One study with 1985 patients found an association with response.<br>OR (95% CI): 0.81 (0.70–0.94). P=0.006.[24]<br><br>No association:<br>No studies				
CYP3A4 (rs1056836) G/C	chr2:38071060				Association:<br>One study with 1985 patients found an association with response.<br>OR (95% CI): 1.36 (1.14–1.63). P=0.00074.[24]<br><br>No association:<br>No studies				
DBC1 (rs10760019) C/T	chr9:119116099				Association:<br>One study with 196 patients found an association with response.<br>P= 2.94E–05.[3]<br><br>No association:<br>No studies				

DHX32<br>(rs12356233)<br>A/G	chr10:125846361	Association:<br>No studies<br><br>No association:<br>One study with 126 patients found no association with response.<br>OR (95% CI): 0.65(0.37–1.15). P=0.14.[25]	Association:<br>One study with 95 patients found an association with response.<br>OR (95% CI): 2.7(1.3–5.61) P=0.0064.[25]<br><br>No association:<br>No studies	Association:<br>No studies<br><br>No association:<br>One study with 127 patients found no association with response.<br>OR (95% CI): 0.85(0.47–1.56). P=0.61.[25]					
DHX57<br>(rs3112165)<br>A/C	chr2:3886304			Association:<br>One study with 134 patients found an association with non-response.<br>OR=19.29<br>P= 2.73×10^{-6}.[5]<br><br>No association:<br>No studies					
ENOX1<br>(rs9594987)<br>T/C	chr13:43656858						Association:<br>One study with 1091 patients found an association with response.<br>P=0.016[16]<br><br>No association:<br>One study with 79 patients found no association with response.<br>P=0.369[15]		

ESR1 (rs3798577) T/C	chr6:152099995				Association: One study with 882 patients found an association with response. OR (95% CI): 0.87 (0.76–0.99). P= 0.031[24]<br><br>No association: No studies				
ESR1 (rs9340799) A/G	chr6:151842246				Association: One study with 882 patients found an association with response. OR (95% CI): 1.18 (1.02–1.36). P=0.019.[24]<br><br>No association: No studies				
ETV7 (rs776432) C/T	chr12:69580872				Association: One study with 867 patients found an association with response. P=0.01[26]<br><br>No association: No studies				
EYA4 (rs17301249) G/C	chr6:133291776				Association: One study with 566 patients found an association with response. P=3.37-04.[27]<br><br>No association: Three studies				

					found no association with response<br>Krintel et al: n=196. P=0.57.[3]<br><br>Márquez et al: n=634. OR (95% CI): 1.04 (0.67-1.61). P=0.967.[28]<br><br>Mirkov et al: n=882. P=0.36[10]				
FAM155A (rs9301169) T/C	chr13:106902717				Association:<br>One study with 196 patients found an association with esponse.<br>OR (95% CI):<br>0.3 (0.18- 0.58).<br>P= 2.40E – 05[3]<br><br>No association:<br>No studies				
FCGR2A (rs1801274) G/A#	chr1:161509955	Association:<br>Two studies found an association with poor response and one with good response.<br><br>Eektimmerman et al: n=291<br>OR (95% CI): 0.63 (0.45–0.91).<br>p=0.011[13]<br><br>Avila-Pedretti et al: n=95	Association:<br>Two studies found an association with poor response.<br><br>Avila-Pedretti et al: n=90<br>OR (95% CI): 0.62 (0.32–1.22).<br>P=0.035[25]<br><br>Montes et al: n=202<br>P=0.0055[30]	Association:<br>No studies.<br><br>No association:<br>One study with 127 patients found no association with response.<br>OR (95% CI): 1.06(0.6–1.9).<br>P= 0.96[25]	Association:<br>One study with 91 patients found an association with response.<br>P=0.035[31]<br><br>No association:<br>No studies.	Association:<br>One study with 52 patients found an association with response.<br>OR (95%CI) = 4.86 (1.12-21.12).<br>P=0.041[32]<br><br>No association:<br>No studies.	Association:<br>One study with 140 patients found an association with response.<br>OR (95%CI) = 9.52 (1.80–14.70).<br>P= 0.007.[33]<br><br>No association:<br>Two studies found no association with response.		Association:<br>One study with 120 patients found an association with response.<br>OR (95%CI) = 2.43 (1.01-5.92)<br>P= 0.048[35]<br><br>No association:<br>No studies.

		OR (95% CI): 0.39 (0.19–0.84). P=0.022[25]<br><br>Dávila-Fajardo et al:<br>n=302<br>OR (95% CI): 1.43 (1.04–1.98). P=0.026[29]<br><br>No association:<br>No studies	No association:<br>No studies.				Morales et al:<br>n=87<br>P=0.934[32]<br><br>Luxembourger et al:<br>n=154. P=0.691[34]		
FCGR2B I232T (rs1050501) T/C	chr1:161674008				Association:<br>One study with 364 patients found an association with response. OR (95% CI): 2.3 (1.1-5.0)<br>P=0.03[36]<br><br>No association:<br>No studies		Association:<br>No studies<br><br>No association:<br>One study with 122 patients found no association with response.<br>P=0.739[34]		
FCGR3A (rs396991) A/C	chr1:161544752	Association:<br>No studies<br><br>No association:<br>One study with 302 patients found no association with response.<br>OR (95% CI): 0.92 (0.66–1.27).<br>P=0.61[29]	Association:<br>One study with 90 patients found an association with response.<br>P=0.04[37]<br><br>No association:<br>One study with 138 found no association with response.<br>P=0.813[38]		Association:<br>One study with 91 patients found an association with response:<br>P=0.040[31]<br><br>No association:<br>Two studies found no association with response:<br><br>Montes et al:<br>n= 377	Association:<br>Three studies found an association with response.<br><br>Quartuccio et al:<br>n=212. OR (95% CI) 4.4 (1.4 - 13.5)<br>P=0.01[40]<br><br>A. Ruyssen-Witrand et al:<br>n=111<br>OR (95% CI): 3.8	Association:<br>One study with 87 patients found an association with response:<br>OR (95% CI) =5.1 (1.2-21.3)<br>P=0.027[32]<br><br>No association:<br>One study with 148 patients found no association with response.		Association:<br>No studies<br><br>No association:<br>Two studies found no association with response:<br>Pete et al:<br>n=120.<br>P=0.615[35]<br><br>Gazeau et al:<br>n=43. P=NS[45]

					OR (95% CI): 1.0 (0.7-1.5). P=0.9[36]<br><br>Kastbom et al: n=282. P=0.80[39]	1.2 - 11.7). P=0.023[41]<br><br>Kastbom et al: n=177 P=0.017[42]<br><br>No association:<br>Three studies found no association with response<br><br>Pál et al: n=52. OR(95% CI): 1.67 (0.39 to 6.97) P=0.48[43]<br><br>Sarsour et al: n=158 P=0.86[44]<br><br>Morales et al: n=55. P=0.719[32]	P=0.661[34]		
FTO (rs7195994) G/A	chr16:54026293		Association:<br>One study with 434 patients found an association with response.<br>P = 9.74E-09[7]<br><br>No association:<br>No studies						
G1m1,17 (rs1071803) T/C	chr14:105742782		Association:<br>One study with 386 patients found an						

			association with response.<br>P=0.001.[46]<br><br>No association:<br>No studies						
GALNT18<br>(rs4910008)<br>C/T	chr11:11458319						Association:<br>Two studies found an association with response.<br><br>Maldonado-Montoro et al:<br>n=79<br>OR (95% CI):<br>0.078 (0.09-0.67)<br>P=0.02[15]<br><br>Wang et al:<br>n=1091<br>P=0.0063[16]<br><br>No association:<br>No studies		
GBP6<br>(rs928655)<br>A/G	chr1:89384015		Association:<br>No studies<br><br>No association:<br>One study with 101 patients found no association with non-response.<br>OR (95% CI):<br>1.17 (0.4-3.1),<br>P=0.75.[9]		Association:<br>One study with 89 patients found an association with non-response.<br>OR (95% CI):<br>5.5 (1.8, 20.2)<br>P= 0.0009[8]<br><br>No association:<br>Three studies found no association with non-response.				

					Krintel et al: n= 196<br>OR (95% CI): 1.3 (0.8- 2.2)<br>P= 0.36.[3]<br><br>Suarez-Gestal et al:<br>n=151<br>OR (95% CI): 0.60 (0.3-1.2), P=0.1.[9]<br><br>Mirkov et al:<br>n=882. P=0.051[10]				
GFRA1 (rs1679568) G/A	chr10:116058040				Association:<br>One study with 444 patients found an association with response. P=8.1x10-7.[47]<br><br>No association:<br>No studies				
GFRA1 (rs7070180) C/T	chr10:116092959				Association:<br>One study with 566 patients found an association with response. P=2.24-04[27]<br><br>No association:<br>One study with 566 patients found no association with non-response.<br>OR (95% CI): 1.3 (0.9-2.1)<br>P=0.2[4]				

GSTP1 (rs1138272) C/T	chr11:67586108				Association:<br>One study with 548 patients found an association with response. OR (95% CI): 1.55 (1.06–2.27). P= 0.023.[24]<br><br>No association:<br>No studies				
GSTP1 (rs1695) A/G	chr11:67585218				Association:<br>One study with 548 patients found an association with response. OR (95% CI): 1.22 (1.02–1.45). P=0.033.[24]<br><br>No association:<br>No studies				
HAS2AS (rs7816916) G/T	chr8:121885033				Association:<br>One study with 196 patients found an association with response. P= 2.73E–05.[3]<br><br>No association:<br>No studies				
IFNG (rs2430561) T/A					Association:<br>One study with 514 patients found an association with response. OR(95% CI): 0.40 (0.21-0.76), P=0.005[48]				

					No association:<br>One study with 974 patients found no association with response.<br>OR(95% CI): 1.00 (0.82-1.23)<br>P=0.98[21]				
IFNGR2 (rs8126756) T/C	chr21:33403138				Association:<br>Two studies found an association with response:<br><br>Sode et al:<br>n=511 patients<br>P= 0.027[49]<br><br>Hassan et al:<br>n=1050<br>P= 0.027[50]<br><br>No association:<br>No studies				
IFNK (rs7046653) G/A	chr9:27490969		Association:<br>No studies<br><br>No association:<br>One study with 101 patients found no association with response. OR (95% CI): 0.95 (0.4-2.1), P=0.9[9]		Association:<br>One study with 89 patients found an association with response.<br>OR (95% CI):<br>4.9 (1.8-14.0).<br>P=0.0004[8]<br><br>No association:<br>Two studies found no association with response.<br><br>Krintel et al:<br>n=196<br>OR (95% CI):				

					1.53 (0.9-2.6)<br>P=0.13[3]<br><br>Suarez-Gestal et al:<br>n=151<br>OR (95% CI): 1.23 (0.7-2.5), P=0.5[9]				
IKBKB<br>(rs10958713)<br>C/T	chr8:42323198	Association:<br>No studies<br><br>No association:<br>One study with 400 patients found no association with response.<br>P=0.685[19]		Association:<br>One study with 386 patients found an association with response<br>P=0.036.[19]<br><br>No association:<br>No studies	Association:<br>No studies<br><br>No association:<br>One study with 909 patients found no association with response[19]				
IKBKB<br>(rs11986055)<br>A/C	chr8:42277660		Association:<br>No studies<br><br>No association:<br>One study with 400 patients found no association with response.<br>P>0.05.[19]	Association:<br>No studies<br><br>No association:<br>One study with 386 patients found no association with response.<br>P>0.05.[19]	Association:<br>One study with 901 patients found an association with response.<br>OR (95% CI): 3.307 (1.184 to 9.239).<br>P=0.022[19]<br><br>No association:<br>One study with 986 patients found no association with response OR (95% CI): 0.83 (0.51-1.35).<br>P=0.44[21]				
IKZF2<br>(rs3768788)<br>A/G	chr2:213151021			Association:<br>One study with 134 patients found an					

				association with response. OR=0.2074 P= 4.46 x 10^{-5}.[5]<br><br>No association:<br>No studies					
IL-6 (rs1800795) C/G	chr7:22727026			Association:<br>One study found an association with response.<br><br>Jancic et al: n=73. P= 0.006[51]<br><br>No association:<br>No studies	Association:<br>One study with 129 patients found no association with response after 6 months. OR (95% CI): 1.27 (0.68-2.35) P= 0.011[53]<br><br>But found an association after 12 months: OR (95% CI): 2.93 (1.29-6.70) P= 0.011[53]<br><br>No association:<br>No studies	Association:<br>One study with 112 patients found an association with response. OR (95%CI): 3.196 (1.204-8.485) P=0.0234[54]<br><br>No association:<br>One study with 53 patients found no association with response. OR(95% CI): 4.8 (0.9-25.5) P=0.062[23]	Association:<br>One study with 184 patients found an association with response. OR (95% CI): 0.270 (0.072-1.005) P= 0.039.[17]<br><br>No association:<br>One study with 140 patients found no association with response. OR (95% CI): 0.58 (0.12-2.67) P=0.50[55]		
IL10 (rs1800896) T/C	chr1:206773552			Association:<br>No studies<br><br>No association:<br>One study with 113 patients found no association with response. P=0.22[56]	Association:<br>One study with 755 patients found an association with response. P=0.01[20]<br><br>No association:<br>Two studies found no association with response	Association:<br>No studies<br><br>No association:<br>One study with 59 patients found no association with response. P=0.417[58]	Association:<br>No studies<br><br>No association:<br>One study with 154 patients found no association with response. P=0.307[34]		

					Caneta et al:<br>n=149<br>OR (95% CI):<br>1.37 (0.67–2.80).<br>P= 0.39[12]<br><br>Pers et al:<br>n=59. P=0.776[57]				
IL10-2849<br>(rs6703630)<br>G/A	chr1:206775294			Association:<br>One study with 50 patients found an association with response.<br>OR (95% CI):<br>3.0 (1.2–7.6).[59]<br><br>No association:<br>No studies					
IL10RA<br>(rs2229114)<br>C/T	chr11:117999163	Association:<br>One study with 150 patients found an association with response.<br>OR (95% CI):<br>2.571 (1.044–6.334). P=0.040[13]<br><br>No association:<br>No studies							
IL12B<br>(rs6887695)<br>G/C	chr5:159395637				Association:<br>One study with 516 patients found an association with response.<br>OR (95% CI): 0.60 (0.40–0.91).				

					P=0.017[49]<br><br>No association:<br>One study with 1007 patients found no association with response. OR (95% CI)0.91 (0.73-1.14). P=0.43[21]				
IL17A (rs2275913) G/A	chr6:52186235				Association:<br>One study with 392 patients found an association with response. OR (95% CI): 1.73 (1.09-2.75), P=0.021[48]<br><br>No association:<br>No studies				
IL18 (rs187238) C/G	chr11:112164265				Association:<br>One study with 507 patients found an association with response. OR (95% CI): 1.52 (1.05–2.19) P=0.026[49]<br><br>No association:<br>No studies.				
IL18 (rs1946518) T/G	chr11:112164735				Association:<br>One study with 507 patients found an association with response. OR (95% CI): 1.52				

					(1.05–2.19)<br>P=0.026[49]<br><br>No association:<br>No studies				
IL1A(-889)<br>(rs1800587)<br>G/A	chr2:112785383							Association:<br>One study with 80 patients found an association with response.<br>OR (95% CI) =<br>4.32 (1.69 - 11.03)<br>P-value= 0.0018[60]<br><br>No association:<br>No studies	
IL1A(+4845)<br>(rs17561)<br>C/A	chr2:112779646							Association:<br>One study with 80 patients found an association with response.<br>OR (95% CI) = 4.85 (1.85 - 12.70)<br>P=0.0009.[60]<br><br>No association:<br>No studies	
IL1B<br>(rs1143623)<br>C/G	chr2:112838252				Association:<br>One study with 538 patients found an association with response.<br>OR (95% CI): 2.14 (1.05-4.35).<br>P=0.037[48]<br><br>No association:<br>No studies				

IL1B<br>(rs4848306)<br>G/A	chr2:112840530		Association:<br>One study with 168 patients found an association with response.<br>OR (95% CI): 2.84(1.39-5.79). P=0.004[48]<br><br>No association:<br>No studies						
IL1B +3954<br>(rs1143634)<br>G/A	chr2:112832813							Association:<br>One study with 80 patients found an association with response.<br>OR (95% CI) = 2.93 (1.17- 7.34)<br>P= 0.02[60]<br><br>No association:<br>No studies	
IL1R1<br>(rs3917243)<br>G/A	chr2:102158528	Association:<br>One study with 291 patients found an association with response.<br>OR (95% CI): 1.464 (1.017–2.107). p=0.040[13]<br><br>No association:<br>No studies							
IL1RAP<br>(rs13321840)<br>G/T	chr3:190557732	Association:<br>One study with 291 patients found an association with							

		response.<br>OR (95% CI): 1.53 (1.04–2.25).<br>p=0.030[13]<br><br>No association:<br>No studies							
IL2RA (rs11594656) T/A	chr10:6080046				Association:<br>One study with 1115 patients found an association with response.<br>OR (95% CI): 1.47 (1.06-2.04).<br>p=0.02[61]<br><br>No association:<br>No studies				
IL6R (rs12083537) A/G	chr1:154408627						Association:<br>Three studies found an association with response[6234,63]<br><br>Enevold et al:<br>n=79. P=0.02[62]<br><br>Maldonado-Montoro et al:<br>n=77.<br>RR(95% CI):<br>1.14 (1.11-2.0)<br>P=0.021[63]<br><br>Luxembourger et al:<br>n=154. P=0.037[34]		

							No association:<br>No studies		
IL6R<br>(rs4329505)<br>T/C	chr1:154459944						Association:<br>One study with 79 patients found an association with response.<br>P=0.02[62]<br><br>No association:<br>Two studies found no association with response.<br><br>Maldonado-Montoro et al: n=77. P=0.902.[63]<br><br>Luxembourger et al: n=154.<br>P= 0.381[34]		
IL6R<br>(rs11265618)<br>C/T	chr1:154457616						Association:<br>One study with 77 patients found an association with response.<br>P=0.031[15]<br><br>No association:<br>No studies		
INFG<br>(rs2069705)<br>G/A	chr12:68161231				Association:<br>One study with 281 patients found an association with response.<br>OR (95% CI): 0.50 (0.30–0.83)<br>P=0.0075[12]				

					No association:<br>No studies				
IRAK3<br>(rs11541076)<br>A/T	chr12:66254548				Association:<br>Two studies found an association with response.<br><br>Sode et al:<br>n=1007<br>OR (95% CI):<br>1.33 (1.00-1.77)<br>P=0.047[21]<br><br>Potter et al:<br>n=909<br>OR (95% CI): 1.467 (1.029 to 2.092).<br>P= 0.034[19]<br><br>No association:<br>No studies				
IRF5<br>(rs2004640)<br>T/G	chr7:128938247					Association:<br>One study with 107 patients found an association with response.<br>P=0.030[64]<br><br>No association:<br>No studies			
KCNIP1/ KCNMB1<br>(rs703505)<br>A/G	chr5:170382398						Association:<br>Two studies found an association with response<br><br>Wang et al:<br>n=1091<br>P=0.031[16]		

							Díaz-Villamarín et al:<br>n=140<br>OR (95% CI):<br>0.29 (0.09- 0.87).<br>P=0.01[65]<br><br>No association:<br>Two studies found no association with response.<br><br>Maldonado-Montoro et al:<br>n=79<br>P=0.120[15]<br><br>Luxembourger et al:<br>n=154<br>P= 0.904[34]		
LASS6 (rs13393173) G/A	chr2:168532581		Association:<br>No studies<br><br>No association:<br>One study with 101 patients found no association with response.<br>OR (95% CI): 0.85 (0.3-2.1), P=0.72[9]		Association:<br>One study with 89 patients found an association with response.<br>OR (95% CI):<br>6.8 (1.7 - 40.3).<br>P = 0.004.[8]<br><br>No association:<br>Three studies found no association with response:<br><br>Krintel et al:<br>n=196				

					OR (95% CI): 1.49 (0.9- 2.6). P=0.17[3]<br><br>Suarez-Gestal et al:<br>n=151<br>OR (95% CI): 0.83 (0.4-1.7), P=0.6[9]<br><br>Mirkov et al:<br>n=882. P=0.71[10]				
LINC01036 (rs12142623) C/A	chr1:187321274				Association:<br>One study with 1821 patients found an association with response.<br>P= 2.04 x 10^{-4}.[10]<br><br>No association:<br>No studies				
LINC01036 (rs4651370) T/A	chr1:187269960				Association:<br>One study with 1821 patients found an association with response.<br>P=1.09x10^{-4}.[10]<br><br>No association:<br>No studies				
LINC01387 (rs4411591) C/T	chr18:6550118				Association:<br>One study with 1821 patients found an association with response.<br>P=5.14x10^{-5}.[10]				

					No association:<br>No studies				
LINC01619<br>(rs7962316)<br>G/A	chr12:92026179				Association:<br>One study with 566 patients found an association with response.<br>P=5.09 x10^{-4} .[27]<br><br>No association:<br>Two studies found no association with response:<br><br>Krintel et al:<br>n=196. P=0.61[3]<br><br>Mirkov et al:<br>n=882. P=0.92[10]				
LINC01762<br>(rs1885800)<br>C/T	chr1:116471110			Association:<br>One study with 134 patients found an association with non-response.<br>OR=19.29.<br>P=2.73 x 10-6.[5]<br><br>No association:<br>No studies.					
LMO4<br>(rs983332)<br>G/T	chr1:87666697		Association:<br>No studies.<br><br>No association:<br>One study with 101 patients found no association with response.		Association:<br>One study with 89 patients found an association with response.<br>OR (95% CI):<br>10.2 (2.6 - 59.2).<br>P= 0.00007[8]				

			OR (95% CI):<br>0.72 (0.3-1.7),<br>P=0.46.[9]		No association:<br>Three studies found no association with response.<br><br>Krintel et al: n= 196<br>OR (95% CI): 1.14 (0.6-2.2). P=0.74.[3]<br><br>Suarez-Gestal et al:<br>n=151<br>OR (95% CI): 1.11 (0.6-2.0) P=0.8.[9]<br><br>Mirkov et al:<br>n=882. P=0.603[10]				
LOC100507254 (rs728371)<br>G/A	chr6:132161147			Association:<br>One study with 134 patients found an association with response.<br>OR=31.21.<br>P=4.01 x 10-6[5]<br><br>No association:<br>No studies					
LOC101928196 (rs703297)<br>A/C	chr1:161544752						Association:<br>One study with 1091 patients found an association with response.<br>OR=0.68.<br>P=0.022[16]		

							No association:<br>No studies		
LOC102723883<br>(rs7767069)<br>T/A	chr6:68060671				Association:<br>One study with 1821 patients found an association with response.<br>P= 8.34 x 10^{-5}.[10]<br><br>No association:<br>No studies				
LOC105369309<br>(rs717117)<br>A/G	chr11:57127131				Association:<br>One study with 196 patients found an association with response.<br>OR (95% CI):<br>10.7 (3.1-36.2).<br>P=9.60 x10^{-6}.[3]<br><br>No association:<br>One study with 566 patients found no association with response.<br>OR (95% CI): 1.5 (0.6-3.7). P=0.4[4]				
LOC105373260<br>(rs10927136)<br>C/T	chr1:244124215			Association:<br>One study with 134 patients found an association with response.<br>OR=4.368 P= 5.35 x 10^{-6}.[5]<br><br>No association:<br>No studies					

LOC105375523 (rs10954517) T/C	chr7:136187199			Association:<br>One study with 134 patients found an association with response.<br>OR=4.992<br>$P=2.52 \times 10^{-5}$. [5]<br><br>No association:<br>No studies					
LOC105375523 (rs834811) A/G	chr7:136199823			Association:<br>One study with 134 patients found an association with response.<br>OR= 5.271<br>$P=1.05 \times 10^{-6.5}$<br><br>No association:<br>No studies					
LOC105376265 (rs10739625) G/T	chr9:123311401				Association:<br>One study with 566 patients found an association with response.<br>$P=9.92 \times 10^{-04}$.[27]<br><br>No association:<br>No studies				
LOC105377785 (rs10108210) C/A	chr8:2743918						Association:<br>Two studies found an association with response.<br><br>Wang et al:<br>n=1091.<br>P=0.028[16]		

							Maldonado-Montoro et al: n=79. P=0.039[15]<br><br>No association:<br>No studies		
LOC105378654 (rs17534243) A/G	chr1:38185245	Association:<br>One study with 291 patients found an association with response.<br>OR (95% CI): 1.7(1.06–2.6). P=0.026.[13]<br><br>No association:<br>No studies							
LOC107986770 (rs1568885) T/A	chr7:13597906				Association:<br>One study with 1821 patients found an association with response.<br>P = 1.69 x 10^{-4}.[10]<br><br>No association:<br>No studies				
LOC727911 (rs629326) G/T	chr6:159075681				Association:<br>One study with 867 patients found an association with response.<br>OR (95% CI): 0.31 (0.15-0.62)<br>P= 0.00[26]<br><br>No association:<br>No studies				

LRRC55<br>(rs12417609)<br>A/G	chr11:57127812				Association:<br>One study with 196 patients found an association with response.<br>OR (95% CI): 10.7 (3.1 - 36.2). P=9.60 x10^{-6}.[3]<br><br>No association:<br>No studies				
LTBP1<br>(rs1454379)<br>G/A	chr2:33214978			Association:<br>One study with 134 patients found an association with response.<br>OR= 6.364<br>P= 1.80 x 10-5.[5]<br><br>No association:<br>No studies					
LY96 (rs11465996)<br>C/G	chr8:73989727		Association:<br>One study with 168 patients found an association with response.<br>OR (95% CI): 2.20(1.11-4.36). P=0.023[48]<br><br>No association:<br>No studies						
MAFB<br>(rs6065221)<br>C/T	chr20:40248820				Association:<br>One study with 361 patients found an association with response.<br>OR (95% CI):				

					0.24 (0.13–0.41).<br>P= 6.26×10^{-6}.[6]<br><br>No association:<br>No studies				
MAFB<br>(rs6028945)<br>G/T	chr20:40192165				Association:<br>One study with 89 patients found an association with response:<br>OR (95% CI):<br>11.2 (2.3 - 108.1).<br>P=0.0004[8]<br><br>No association:<br>Three studies found no association with response<br><br>Krintel et al:<br>n=196<br>OR (95% CI):<br>0.86 (0.4, 1.6).<br>P=0.72.[3]<br><br>Suarez-Gestal et al: n=151<br>OR (95% CI): 1.09 (0.5-2.5). P=0.9[9]<br><br>Mirkov et al:<br>n=882. P=0.78[10]				
MAFB<br>(rs6071980)<br>T/C	chr20:40239936				Association:<br>One study with 89 patients found an association with response:<br>OR (95% CI):<br>7.6 (1.9 - 44.6)				

					P=0.0009[8]<br><br>No association:<br>Three studies found no association with response<br><br>Krintel et al: n=196<br>OR (95% CI): 0.59 (0.3 - 1.2) P=0.16.[3]<br><br>Suarez-Gestal et al: n=151<br>OR (95% CI): 0.83 (0.4-2.0). P=0.6[9]<br><br>Mirkov et al: n=882. P=0.74[10]				
MAP2K6 (rs2716191) T/C	chr17:6954018				Association:<br>One study with 1102 patients found an association with response. P=0.050[66]<br><br>No association:<br>One study with 755 patients found no association with response.<br>OR (95% CI): 1.11 (0.8-1.6). P=0.5[20]				
MAP2K6 (rs11870477)					Association:<br>One study with 196 patients found				

A/C					an association with response. P= 3.30E–06[3]<br><br>No association:<br>Two studies found no association with response.<br><br>Mirkov et al: n= 882 P=0.67[10]<br><br>Lopez-Rodriguez et al:<br>n=581. OR (95% CI): 1.0 (0.6-1.5) P=0.9[4]				
MAP3K1 (rs96844) A/G,	chr5:56900777				Association:<br>One study with 1070 patients found an association with response. P=0.025[67]<br><br>No association:<br>No studies				
MAP3K14 (rs4792847) G/A	chr17:45306118				Association:<br>One study with 1028 patients found an association with response. P=0.0361[67]<br><br>No association:<br>No studies				
MAPK14 (rs916344)	chr6:36121162				Association:<br>One study with 1102 patients				

C/G					found an association with response. (According to study, but P>0.05).[66]<br><br>No association:<br>One study with 755 patients found no association with response. OR (95% CI): 1.16 (0.7-2.0) P= 0.6.[20]				
MAPKAPK2 (rs4240847) C/A	chr1:206723277				Association:<br>One study with 1102 patients found an association with response. (According to study, but P>0.05).[66]<br><br>No association:<br>One study with 755 patients found no association with response. OR (95% CI): 1.42 (0.9-2.3). P= 0.14.[20]				
MED15 (rs113878252) C/T	chr22:20541640			Association:<br>One study with 361 patients found an association with response.					

				OR (95% CI):<br>0.09 (0.04-<br>0.24). P=1.24 x<br>10^{-8}.[68]<br><br>No association:<br>No studies					
MED29<br>(rs10414216)<br>T/C	chr19:39400521			Association:<br>One study with<br>275 patients<br>found an<br>association<br>with response<br>P=0.003[69]<br><br>No association:<br>No studies		Association:<br>One study with 61<br>patients found an<br>association with<br>response<br>P=0.025[69]<br><br>No association:<br>No studies			
MMP20/MMP27<br>(rs948138) G/A	chr11:102630934			Association:<br>One study with<br>615 patients<br>found an<br>association<br>with response.<br>P= 7.62 x 10^{-8}<br>.[7**]<br><br>No association:<br>No studies					
MOBKL2B<br>(rs868856)<br>A/G	chr9:27489253		Association:<br>One study with 89<br>patients found an<br>association with<br>response.<br>OR (95% CI):<br>4.9 (1.8- 14.0)<br>P= 0.0005[8]<br><br>No association:<br>One study with<br>101 patients		Association:<br>One study with 89<br>patients found an<br>association with<br>response.<br>OR (95% CI):<br>4.9 (1.8- 14.0)<br>P= 0.0005[8]<br><br>No association:<br>Three studies<br>found no				

			found no association with response.<br>OR (95% CI): 1.01 (0.5-2.2), P=0.98[9]		association with response.<br><br>Krintel et al:<br>n=196<br>OR (95% CI):<br>1.53 (0.9- 2.6)<br>P= 0.13[3]<br><br>Suarez-Gestal et al:<br>n=151<br>OR (95% CI): 1.19 (0.6-2.0), P= 0.6[9]<br><br>Mirkov et al:<br>n=882. P=0.65[10]				
MOBKL2B (rs2814707) C/T	chr9:27536399				Association:<br>Two studies found an association with response:<br><br>Krintel et al:<br>n=196<br>OR (95% CI): 1.85 (1.0 - 3.3)<br>P= 0.03.[3] ***<br><br>Liu et al:<br>n= 89<br>OR (95% CI):<br>5.2 (1.8- 16.7)<br>P= 0.0006[8]<br><br>No association:<br>Two studies found no association with response				

					Suarez-Gestal et al:<br>n= 151 patients<br>OR (95% CI):<br>1.22 (0.6-2.5).<br>P= 0.6[9]<br><br>Mirkov et al:<br>N=882. P=0.95[10]				
MyD88<br>(rs7744)<br>A/G	chr3:38142530		Association:<br>No studies<br><br>No association:<br>One study with 400 patients found no association with response.<br>P=0.338[19]	Association:<br>One study with 386 patients found an association with response.<br>P=0.006[19]<br><br>No association:<br>No studies	Association:<br>One study with 909 patients found an association with response.<br>OR (95% CI):<br>1.57 (1.075 - 2.30)<br>P=0.020[19]<br><br>No association:<br>Three studies found no association with response.<br><br>Zervou et al:<br>n=183<br>OR (95% CI):<br>0.67 (0.19-2.29).<br>P=0.5[2]<br><br>Ferreiro- Iglesias et al: n=755<br>OR (95% CI):<br>1.26 (0.8-1.9).<br>P=0.3[20]<br><br>Sode et al:<br>n=1007				

					OR (95% CI): 0.89 (0.67-1.17). P=0.39[21]				
NACC2 (rs884559) C/T	chr9:136047816				Association: One study with 196 patients found an association with response. P= 2.94E–05.[3]<br><br>No association: No studies				
nearBACE2 (rs2837960) T/G	chr21:41139991	Association: One study with 291 patients found an association with response. OR (95% CI): 1.633 (1.006–2.649). P=0.047[13]<br><br>No association: No studies							
NFkBIA (rs2233407) T/A	chr14:35405317		Association: One study with 400 patients found an association with response. P=0.014[19]<br><br>No association: No studies	Association: No studies<br><br>No association: One study with 386 patients found no association with response. P=0.565[19]	Association: One study with 909 patients found an association with response. P=NS[19]<br><br>No association: No studies				
NFkBIB (rs3136645) T/C	chr19:38908009				Association: One study with 902 patients found an association with response. OR (95% CI): 0.647				

					(0.471 to 0.889)<br>P= 0.007[19]<br><br>No association:<br>One study with 982 patients found no association with response.<br>OR (95% CI): 0.90 (0.69-1.17)<br>P=0.45[21]				
NFKBIB (rs9403) C/G	chr19:38915527				Association:<br>One study with 909 patients found an association with response.<br>OR (95% CI): 0.75 (0.579 - 0.982).<br>P=0.036[19]<br><br>No association:<br>One study with 1007 patients found no association with response.<br>OR (95% CI): 0.84 (0.68-1.05).<br>P=0.13[21]				
NFκB-2 (rs11574851) C/T	chr10:102401202		Association:<br>No studies<br><br>No association:<br>One study with 400 patients found no association with response.<br>P>0.05.[19]	Association:<br>No studies<br><br>No association:<br>One study with 386 patients found no association with response.<br>P>0.05.[19]	Association:<br>One study with 909 patients found an association with response.<br>P= 0.023[19]<br><br>No association:<br>No studies				

NKAIN2<br>(rs9401758)<br>C/T	chr6:124616828			Association:<br>One study with 134 patients found an association with response OR=7.167 P=1.00 x 10-5.[5]<br><br>No association:<br>No studies					
NKG2A<br>(rs2734440)<br>A/G	chr12:10449478				Association:<br>One study with 282 patients found an association with response.<br>OR (95% CI):<br>2.79 (1.05–7.98).<br>P= 0.025[18]<br><br>No association:<br>No studies				
NKG2A<br>(rs7301582)<br>C/T	chr12:10449091				Association:<br>One study with 284 patients found an association with response.<br>OR (95% CI) = 3.68 (1.14–18.95).<br>P=0.019[18]<br><br>No association:<br>No studies				
NKG2D<br>(rs1049174)<br>G/C	chr12:10372766	Association:<br>No studies<br><br>No association:<br>One study with 92 patients found no association with			Association:<br>One study with 280 patients found an association with response.<br>OR (95% CI) = 4.02 (1.45, 12.87).				

		response.<br>P>0.05[70]			P=0.004[70]<br><br>No association:<br>No studies				
NKG2D<br>(rs2255336)<br>T/C	chr12:10379727	Association:<br>No studies<br><br>No association:<br>One study with 92 patients found no association with response.<br>P=NS[70]		Association:<br>One study with 151 patients found an association with response., OR (95%CI) = 0.00 (0.00-0.82) P=0.027[70]<br><br>No association:<br>No studies	Association:<br>One study with 280 patients found an association with response. OR (95%) = 6.88 (1.62-61.80). P=0.003[70]<br><br>No association:<br>No studies				
NLRP1<br>(rs878329)<br>G/C	chr17:5649930		Association:<br>One study with 168 patients found an association with response.<br>OR (95% CI):<br>2.54 (1.20-5.38).<br>P= 0.015[49]<br><br>No association:<br>No studies						
NLRP3<br>(rs10754558)<br>G/C	chr1:247448734		Association:<br>One study with 168 patients found an association with response.<br>OR (95% CI):<br>0.46 (0.22-0.94).<br>P=0.032[49]<br><br>No association:<br>No studies		Association:<br>One study with 509 patients found an association with response.<br>OR (95% CI): 0.61 (0.39–0.96),<br>P=0.033[49]<br><br>No association:<br>No studies				

NLRP3<br>(rs10925026)<br>A/C	chr1:247440956				Association:<br>Two studies found an association with poor response[114].<br><br>Mathews et al:<br>n=1278<br>RR (95% CI) = 0.94 (0.90 to 0.99).<br>P=0.017[11]<br><br>Lopez-Rodriguez et al:<br>n=566<br>OR (95% CI): 0.71 (0.53-0.99),<br>P=0.037[4]<br><br>No association:<br>No studies				
NLRP3<br>(rs4612666)<br>C/T	chr17:69404305		Association:<br>One study with 168 patients found an association with response.<br>OR (95% CI): 0.50(0.25-0.97).<br>P=0.041[48]<br><br>No association:<br>No studies		Association:<br>One study found an association with response.<br><br>Sode et al:<br>n=516.<br>OR (95% CI): 0.62 (0.42-0.92).<br>P=0.018.[48]<br><br>No association:<br>Two studies found no association with response.<br>Lopez-Rodriguez				

					et al<br>n=566<br>LOR (95% CI): 0.77 (0.50-1.11).<br>P=0.2.[4]<br><br>Sode et al:<br>n=469<br>OR (95% CI): 0.75 (0.54-1.04)<br>P=0.08.[21]				
NLRP3 (rs4925648) A/G	chr11:23496859				Association:<br>One study with 1278 patients found an association with response.<br>RR (95% CI): 1.15 (1.01 - 1.31).<br>P= 0.035[11]<br><br>No association:<br>One study with 566 patients found no association with response.<br>OR (95% CI): 0.9 (0.4-1.7). P=0.7[4]				
NLRP3 (rs4925659) G/A	chr1:247440161				Association:<br>One study with 1278 patients found an association with response.<br>RR (95% CI): 1.06 (1.02 to 1.11)<br>P= 0.0064[11]<br><br>No association:				

					One study with 566 patients found no association with response.<br>OR (95% CI): 0.8 (0.6-1.1). P=0.2[4]				
No gene<br>(Potenital: MAP3K7, BACH2)<br>(rs284515)<br>A/G	chr6:90499109				Association:<br>One study with 444 patients found an association with response.<br>P= 6.6x10-7[47]<br><br>No association:<br>One study with 532 found no association with response.<br>OR (95% CI): 0.8 (0.6-1.1), P=0.2[4]				
No gene<br>(rs1024125)<br>T/C	chr2:173843471				Association:<br>One study with 566 patients found an association with response.<br>P= 8.54^{-04}[27]<br><br>No association:<br>No studies				
No gene<br>(rs10507815)<br>A/G	chr13:73465744			Association:<br>One study with 134 patients found an association with response.<br>OR=5.08<br>P=1.15 x 10-5[5]<br><br>No association:<br>No studies					

No gene (rs10521044) G/A	chr16:51844512			Association:<br>One study with 134 patients found an association with response.<br>OR= 9.39<br>P=4.10 x 10-6[5]<br><br>No association:<br>No studies					
No gene (rs12081765) A/G	chr1:165372612				Association:<br>One study with 566 patients found an association with response.<br>P=7.52^{-04}.[27]<br><br>No association:<br>Three studies found no association with response.<br><br>Krintel et al n=196 P=0.94.[3]<br><br>Márquez et al:<br>n=634<br>OR (95% CI):<br>1.15 (0.86-1.55).<br>P=0.379.[28]<br><br>Mirkov et al:<br>n=882. P=0.73[10]				
No gene (rs1350948) A/G	chr11:23496859				Association:<br>One study with 566 patients found an association				

					with response.<br>P= 8.65-04.[27]<br><br>No association:<br>Two studies found no association with response.<br><br>Krintel et al: n= 196. P=0.34.[3]<br><br>Mirkov et al: n=882. P=0.36[10]				
No gene (rs1395000) A/G	chr4:83919529			Association:<br>One study with 134 patients found an association with response.<br>OR=7.83.<br>P= 1.15 x 10-5.[5]<br><br>No association:<br>No studies					
No gene (rs1447722) C/G	chr3:139835611				Association:<br>One study with 1821 patients found an association with response.<br>P= 1.62 x 10^{-4}.[10]<br><br>No association:<br>No studies				
No gene (rs2137986) A/G	chr13:61136432			Association:<br>One study with 134 patients found an association with non-					

				response.<br>OR=5.741<br>P= 2.97 x 10^{-6}.[5]<br><br>No association:<br>No studies					
No gene<br>(rs4931337)<br>G/A	chr12:30446149			Association:<br>One study with 134 patients found an association with response.<br>OR=0.1775<br>P=1.50 x 10-5[5]<br><br>No association:<br>No studies					
No gene<br>(rs7305646)<br>T/C	chr12:17111403				Association:<br>One study with 566 patients found an association with response.<br>P=9.16x 10^{-4}.[27]<br><br>No association:<br>Three studies found no association with response.<br><br>Krintel et al:<br>n= 196. P=0.74[3]<br><br>Márquez et al:<br>n=634. OR (95% CI): 0.86 (0.64-1.15). P=0.336.[28]<br><br>Mirkov et al:<br>n=882. P=0.39.[10]				

No gene (rs895822) T/G	chr8:54356354			Association: One study with 134 patients found an association with response OR=6.006 $P=1.73 \times 10^{-6}$.[5]<br><br>No association: No studies					
NR2F2 (rs10520789) G/A	chr15:95598638				Association: One study with 196 patients found an association with response. $P= 5.64 \times 10^{-7}$.[3]<br><br>No association: One study with 882 patients found no association with response. P=0.47[10]				
NR2F2 (rs16973982) T/C	chr15:95619929				Association: One study with 196 patients found an association with response. $P= 2.79 \times 10^{-6}$.[3]<br><br>No association: Two studies found no association with response.<br><br>Mirkov et al: n= 882 P=0.47[10]				

					Lopez-Rodriguez et al:<br>n=581. OR (95% CI): 0.9 (0.5-1.5). P=0.7[4]				
NUBPL (rs2378945) G/A	chr14:31831584				Association:<br>One study with 1821 patients found an association with response.<br>P = 6.88 x 10^{-4}.[10]<br><br>No association:<br>No studies				
OR7E156P (rs9539845) T/C	chr13:63696126				Association:<br>One study with 196 patients found an association with response.<br>OR (95% CI):<br>3.1 (1.8 - 5.5).<br>P=2.80 x10^{-5}.[3]<br><br>No association:<br>No studies				
PADI4 (rs2240340)	chr1:17336144	Association:<br>One study with 291 patients found an association with response.<br>OR (95% CI):<br>1.547 (1.080–2.218). p=0.017[13]<br><br>No association:<br>No studies			Association:<br>No studies<br><br>No association:<br>One study with 208 patients found no association with response.<br>OR (95% CI): 0.79 (0.54, 1.16).<br>P= 0.22[71]				

PDE3A (rs11045392) T/C	chr12:20687905				Association: One study with 196 patients found an association with response. OR (95% CI): 3.3 (1.9, 5.6). P=1.70E – 06[3]<br><br>No association: No studies				
PDSS1 (rs11015244) C/T	chr10:26715912			Association: One study with 134 patients found an association with response OR=3.75 P=4.61 x 10^{-5}.[5]<br><br>No association: No studies					
PDZD2 (rs10039073) G/A	chr5:32042654			Association: One study with 134 patients found an association with response OR: 0.25. P=0.002[5]<br>No association: No studies					
PDZD2 (rs1532269) C/G	chr5:32018735				Association: One study with 566 patients found an association with response.				

					P= 7.11-04[27]<br><br>No association:<br>Three studies found no association with response.<br><br>Krintel et al: n=196 P=0.36.[3]<br><br>Márquez et al: n=634. OR (95% CI): 0.93 (0.68-1.28). P=0.715[28]<br><br>Mirkov et al: n=882. P=0.92[10]				
PGR (rs518162) A/G	chr11:101129770				Association:<br>One study with 548 patients found an association with response. OR (95% CI): 0.80 (0.67–0.96). P=0.017.[24]<br><br>No association:<br>No studies				
PON1 (rs854547) G/A	chr7:95294544		Association:<br>No studies<br><br>No association:<br>One study with 101 patients found no association with response.<br>OR (95% CI): 0.95 (0.45-2.1), P=0.91[9]		Association:<br>One study with 89 patients found an association with response.<br>OR (95% CI): 3.6 (1.5-9.3).<br>P= 0.003[8]<br><br>No association:				

					Three studies found no association with response.<br><br>Krintel et al:<br>n=196<br>OR (95% CI):<br>0.98 (0.6-1.6)<br>P=1.00[3]<br><br>Suarez-Gestal et al:<br>n=151<br>OR (95% CI): 1.12 (0.6-2.1), P=0.7[9]<br><br>Mirkov et al:<br>n=882. P=0.52[10]				
PON1 (rs854548) A/G	chr7:95296508		Association:<br>No studies<br><br>No association:<br>One study with 101 patients found no association with response. OR (95% CI): 0.85 (0.3-2.1), P=0.72[9]		Association:<br>One study with 89 patients found an association with response.<br>OR (95% CI):<br>8.5 (2.6-36.5)<br>P= 0.00004[8]<br><br>No association:<br>Three studies patients found no association with response.<br><br>Krintel et al:<br>n= 196<br>OR (95% CI):<br>1.15 (0.7-2.0)<br>P= 0.68[3]				

					Suarez-Gestal et al:<br>n=151<br>OR (95% CI): 1.03 (0.5-2.0), P=0.9[9]<br><br>Mirkov et al:<br>n=882. P=0.61[10]				
PON1 (rs854555) A/C	chr7:95301079		Association:<br>No studies<br><br>No association:<br>One study with 101 patients found no association with response. OR (95% CI): 0.78 (0.4-1.7), P=0.55[9]		Association:<br>One study with 89 patients found an association with response.<br>OR (95% CI):<br>4.6 (1.8- 12.3)<br>P= 0.0006[8]<br><br>No association:<br>Three studies found no association with response.<br><br>Krintel et al:<br>n=196.<br>OR (95% CI):<br>0.99 (0.6- 1.6)<br>P= 1.00[3]<br><br>Suarez-Gestal et al:<br>n=151<br>OR (95% CI): 1.14 (0.6-2.0), P=0.7[9]<br><br>Mirkov et al:<br>n=882. P=0.61[10]				

PRTG<br>(rs4412918)<br>T/C	chr15:55778930				Association:<br>One study with 196 patients found an association with response.<br>P= 1.76E–05.[3]<br><br>No association:<br>No studies				
PTGS2<br>(rs2206593)<br>A/G	chr1:186673297				Association:<br>One study with 903 patients found an association with response.<br>OR (95% CI): 0.515 (0.307 to 0.862).<br>P= 0.012[19]<br><br>No association:<br>No studies				
PTPN2<br>(rs7234029)<br>A/G	chr18:12877061	Association:<br>One study with 64 patients found an association with response.<br>OR (95% CI):<br>0.26 (0.07-0.97).<br>P=0.038[72]<br><br>No association:<br>No studies		Association:<br>No studies<br><br>No association:<br>One study with 107 patients found no association with response.<br>OR (95% CI):<br>0.82 (0.33-2.02)<br>P=0.66[72]					
PTPRC<br>(rs10919563)<br>A/T	chr1:198731313				Association:<br>Three studies found an association with response.<br><br>Cui et al:<br>n=1283		Association:<br>No studies<br><br>No association:<br>One study with 154 patients found no association with		

					OR: 0.59.<br>P=0.0004[14]<br><br>Plant et al:<br>n=1115<br>OR (95% CI): 0.62 (0.40, 0.95).<br>P=0.03[61]<br><br>Iglesias et al:<br>n=755. P=0.007[20]<br><br>No association:<br>Two studies found no association with response.<br><br>Zervou et al:<br>n=183.<br>OR (95% CI): 1.22 (0.54-2.72).<br>P=0.64[2]<br><br>Canhão et al:<br>N=383<br>OR (95% CI): 1.68 (0.88, 3.21)<br>P= 0.12[71]		response.<br>P=0.071[34]		
PTPRC (rs6683595) C/T	chr1:198734177				Association:<br>One study with 755 patients found an association with response.<br>OR (95% CI): 0.64 (0.4-0.97).<br>P=0.03[20]<br><br>No association:<br>No studies				

QKI (rs10945919) A/G	chr6:163765645		Association:<br>No studies<br><br>No association:<br>One study with 101 patients found no association with response.<br>OR (95% CI): 1.32 (0.5-3.2), P=0.54[9]		Association:<br>One study with 89 patients found an association with response.<br>OR (95% CI): 4.6 (1.8, 12.3). P=0.0007[8]<br><br>No association:<br>Three studies found no association with response.<br><br>Krintel et al:<br>n=196 patients<br>OR (95% CI): 1.25 (0.7-2.1). P=0.43[3]<br><br>Suarez-Gestal et al:<br>n=151. OR (95% CI): 0.79 (0.4-1.7), P=0.5[9]<br><br>Mirkov et al:<br>n=882. P=0.44[10]				
QPCT (rs11124586) G/A	chr2:37501324				Association:<br>One study with 196 patients found an association with response.<br>P= 2.03E –05.[3]<br><br>No association:<br>No studies				
QPCT (rs960902) G/A	chr2:37504522				Association:<br>One study with 196 patients found				

					an association with response. P= 8.02E –06.[3]<br><br>No association:<br>No studies				
RGS12 (rs2857859) C/T	chr4:3320413	Association:<br>No studies<br><br>No association:<br>One study with 95 patients found no association with response.<br>OR (95% CI): 0.98(0.45–2.15). p=0.96[25]	Association:<br>One study with 90 patients found an association with response only in anti-CCP positive patients:<br>OR (95% CI): 0.4(0.17–0.99). P=0.042[25]<br><br>No association:<br>No studies	Association:<br>No studies<br><br>No association:<br>One study with 127 patients found no association with response.<br>OR (95% CI): 0.89(0.47–1.71). P= 0.73[25]					
RGS12 (rs4690093) A/G	chr4:3410469	Association:<br>One study with 95 patients found an association with response. OR (95% CI): 0.4(0.17–0.98). P=0.040.[25]<br><br>No association:<br>No studies	Association:<br>No studies<br><br>No association:<br>One study with 126 patients found no association with response. OR (95% CI): 0.95(0.52–1.71). P=0.85.[25]	Association:<br>No studies<br><br>No association:<br>One study with 127 patients found no association with response.<br>OR (95% CI): 0.88(0.44–1.76) P=0.71.[25]					
SHH (rs12698335) A/G	chr7:155889038				Association:<br>One study with 196 patients found an association with response.<br>OR (95% CI): 3.9 (2.1-7.5). P=1.50E–05.[3]				

					No association:<br>No studies				
SLC9A7<br>(rs7055107)<br>G/T	Chr10:46712049						Association:<br>Two studies found an association with response.<br><br>Díaz-Villamarín et al: n=184<br>OR (95% CI): 0.42 (0.18-0.99)<br>P=0.04[17]<br><br>Wang et al:<br>n=1091<br>P=0.006[16]<br><br>No association:<br>No studies		
SLCO1C1<br>(rs3794271)<br>G/A	chr12:2070715				Association:<br>Two studies found an association with response.<br><br>Acosta-Colman et al: n= 313<br>OR (95% CI): 2.63 (1.68–4.12).<br>P=1.74 × 10^{-5}.[73]<br><br>Krintel et al:<br>n= 196<br>OR being nonresponder 95% CI): 3.2 (1.9, 5.5).<br>P=3.50 x10^{-6}.[3]<br><br>No association:				

					Two studies found no association with response.<br><br>Smith et al:<br>n=1750<br>OR (95% CI): 1.01 (0.84–1.22).<br>P= 0.91[74]<br><br>Lopez-Rodriguez et al:<br>n=566<br>OR (95% CI):<br>0.7 (0.5-1.0)<br>P=0.064[4]				
STAT4 (rs10181656) G/C	chr2:191105153				Association:<br>One study with 1012 patients found an association with response.<br>P=0.038[1]<br><br>No association:<br>No studies				
STAT4 (rs7574865) T/G	chr17:69404305	Association:<br>No studies<br><br>No association:<br>One study with 64 patients found an association with response.<br>OR (95% CI): 0.42 (0.12-1.44).<br>P=0.16[72]		Association:<br>One study with 71 patients found an association with response.<br>OR (95% CI):<br>0.16 (0.03-0.78)<br>P= 0.013[72]<br><br>No association:<br>No studies	Association:<br>One study with 1012 patients found an association with response.<br>P=0.028[1]<br><br>No association:<br>Two studies found no association with response.	Association:<br>No studies<br><br>No association:<br>Two studies found no association with response.<br><br>Daïena et al:<br>n=63. P=0.678.[58]<br><br>Juge et al:<br>n= 115. P=0.284.[64]			

					Zervou et al:<br>n=183<br>OR (95% CI):<br>1.35 (0.73-2.48).<br>P=0.23[2]<br><br>Ozen et al:<br>n=141. P=0.19[75]				
STK10<br>(rs2279515)<br>T/C	chr5:172126829			Association:<br>One study with 134 patients found an association with secondary failure.<br>OR: 4.853<br>P=1.32 x 10^{-5}.[5]<br><br>No association:<br>No studies					
STK10<br>(rs2306963)<br>T/C	chr5:172106625			Association:<br>One study with 134 patients found an association with secondary failure.<br>OR=3.947. P= 3.76 x 10^{-5}.[5]<br><br>No association:<br>No studies					
STK10<br>(rs3111491)<br>A/C	chr5:172115873			Association:<br>One study with 134 patients found an association with secondary failure.					

				OR=4.688<br>P=4.39 x 10^{-5}.[5]<br><br>No association:<br>No studies					
SULT1A1<br>(rs9282861)<br>C/T	chr16:28606193				Association:<br>One study with 548 patients found an association with response.<br>OR (95% CI): 0.74 (0.56–0.99).<br>P=0.042.[24]<br><br>No association:<br>No studies				
TANK<br>(rs1267067)<br>T/C	chr2:161151237	Association:<br>One study with 291 patients found an association with response.<br>OR (95% CI): 1.719 (1.145–2.581).<br>P=0.0090[13]<br><br>No association:<br>No studies							
TEC<br>(rs4694890)<br>C/G	chr14:90946417				Association:<br>One study with 566 patients found an association with response.<br>P=7.09^{-04}.[27]<br><br>No association:<br>Two studies found no association with response:				

					Krintel et al: n=196. P=0.44.[3]<br><br>Mirkov et al: n=882. P=0.73[10]				
TGF-beta-1-10 (rs1800470) G/A	chr19:41353016					Association:<br>One study with 63 patients found an association with response. OR (95% CI) = 1.6 (1.2–2.3) P = 0.002.[58]<br><br>No association:<br>No studies			
TGF-beta-1-25 (rs1800471) C/G	chr19:41352971			Association:<br>No studies<br><br>No association:<br>One study with 123 patients found no association with response. P=1.00[56]		Association:<br>One study with 63 patients found an association with response. OR (95% CI) = 1.6 (1.3–1.9) P = 0.025[58]<br><br>No association:<br>No studies			
TLR-2 (rs11935252) A/G	chr4:153710910		Association:<br>No studies<br><br>No association:<br>One study with 400 patients found no association with response. P=0.794[19]	Association:<br>One study with 386 patients found an association with response. P=0.025[19]<br><br>No association:<br>One studies	Association:<br>No studies<br><br>No association:<br>One study with 909 patients found no association with response. P>0.05[19]				

TLR-2<br>(rs2289318)	chr4:153712582				Association:<br>One study with 901 patients found an association with response.<br>OR (95% CI): 0.736 (0.546 - 0.993).<br>P= 0.045[19]<br><br>No association:<br>No studies				
TLR-2<br>(rs5743704)<br>C/A	chr4:153704799		Association:<br>No studies<br><br>No association:<br>One study with 400 patients found no association with response.<br>P= 0.231[19]	Association:<br>One study with 386 patients found an association with response.<br>P=0.006[19]<br><br>No association:<br>No studies	Association:<br>No studies<br><br>No association:<br>One study with 909 patients found no association with response.<br>P>0.05[19]				
TLR1<br>(rs4833095)<br>T/C	chr4:38798089				Association:<br>One study with 511 patients found an association with response.<br>OR (95% CI):<br>2.80 (1.07–7.35).<br>P=0.037[49]<br><br>No association:<br>One study with 993 patients found no association with response.<br>OR (95% CI):<br>0.87 (0.68-1.11)<br>P=0.27[21]				

TLR10 (rs11096957) T/G	chr4:38774870				Association:<br>One study with 909 patients found an association with response. OR (95% CI): 0.736 (0.546 to 0.993). P= 0.045[76]<br><br>No association:<br>One study with 987 patients found no association with response. OR (95% CI), 0.85(0.65-1.12). P=0.26.[21]				
TLR4 (rs12377632) T/C	chr9:117710452				Association:<br>One study with 386 patients found an association with response. OR (95% CI): 0.63(0.41-0.98). P=0.042.[48]<br><br>No association:<br>No studies				
TLR4 (rs2722824) C/A	chr9:117969847				Association:<br>One study with 196 patients found an association with response. P= $1.93x10^{-5}$.[3]<br><br>No association:<br>No studies				
TLR4 (rs4986790)	chr9:117713024				Association:<br>One study with 87 patients found an				

A/G					association with response. P=0.012[77]<br><br>No association:<br>No studies				
TLR4 (rs5030728) G/A	chr9:117712004				Association:<br>One study with 514 patients found an association with response. OR (95% CI): 1.58 (1.06-2.35). P=0.023[48]<br><br>No association:<br>No studies				
TLR4 (rs7045953) A/G	chr9:117723517		Association:<br>One study with 400 patients found an association with response. P=0.039[19]<br><br>No association:<br>No studies	Association:<br>No studies<br><br>No association:<br>One study with 386 patients found no association with response. P=0.776[19]	Association:<br>No studies<br><br>No association:<br>One study with 909 patients found no association with response. P>0.05[19]				
TLR5 (rs5744174) A/G	chr1:223111186		Association:<br>One study with 168 patients found an association with response. OR (95% CI): 2.65 (1.18-5.91). P= 0.018.[49]<br><br>No association:<br>No studies	Association:<br>One study with 166 patients found an association with response. OR (95% CI): 2.34 (1.13-4.84) P=0.022[49]<br><br>No association:<br>No studies	Association:<br>One study with 511 patients found an association with response. OR (95% CI): 1.96 (1.18–3.25). P=0.009[49]<br><br>No association:<br>One study with 988 patients found no association				

					with response.<br>OR (95% CI):<br>1.06 (0.87-1.30).<br>P=0.55[21]				
TLR9<br>(rs187084)<br>A/G	chr3:52227015				Association:<br>One study with 87 patients found an association with response.<br>P=0.018.[77]<br><br>No association:<br>No studies				
TNFα -857<br>(rs1799724)<br>C/T	chr6:31574705	Association:<br>No studies<br><br>No association:<br>One study with 388 patients found no association with response.<br>P=0.11.[78]	Association:<br>One study with 91 patients found an association with response<br>OR (95% CI) = 0.1 (0.01-0.8)<br>P=0.02[79]<br><br>No association:<br>No studies	Association:<br>One study with 32 patients found an association with response.<br>OR (95% CI):<br>12 (1.4–105).<br>P=0.0077[80]<br><br>No association:<br>No studies	Association:<br>One study with 279 patients found an association with response.<br>P = 0.012[81]<br><br>No association:<br>One study with 100 patients found no association with response.<br>P=0.323.[82]	Association:<br>No studies<br><br>No association:<br>One study with 62 patients found no association with response.<br>P=0.644[58]			
TNFα-238<br>(rs361525)<br>G/A	chr6:31575324	Association:<br>No studies<br><br>No association:<br>One study with 388 patients found no association with response<br>P=0.18[78]	Association:<br>One study with 453 patients found an association with response.<br>P=0.033[83]<br><br>No association:<br>One study with 113 patients found no association with response.	Association:<br>No studies<br><br>No association:<br>Two studies found no association with response.<br><br>Maxwell et al:<br>n=455<br>P=0.424[83]	Association:<br>One study with 1050 patients found an association with response.<br>P=0.028[83]<br><br>No association:<br>Two studies found no association with response.				

			OR (95% CI) 1.19 (0.36- 3.91). P=0.785[84]	Kang et al: n=70. OR (95% CI): 0.70 (0.04–12). P=1.0[80]	Swierkot et al: n=278. P=NS[81]<br><br>Sode et al: n=538<br>OR (95% CI): 1.74 (0.63-4.79). P=0.284[48]				
TNFα-308 (rs1800629) G/A	chr6:31575254	Association:<br>Two studies found an association with response.<br><br>Eektimmerman et al: n=291<br>OR (95% CI): 1.8 (1.037–3.12) P=0.037.[13]<br><br>Cuchacovich et al: n=70. P=0.05.[85]<br><br>No association:<br>One study with 388 patients found no association with response. P=0.18.[78]	Association:<br>One study with 59 patients found an association with response.<br>OR= 1.93<br>P= 0.0086.[86]<br><br>No association:<br>Three studies found no association with response.<br><br>Marotte et al: n=198 P>0.05.[87]<br><br>Maxwell et al: n=206. P=0.179.[83]<br><br>Pinto et al: n=113. OR (95% CI): 0.95 (0.40-2.25) P=0.918.[84]	Association:<br>One study with 455 patients found an association with response. P=0.018.[83]<br><br>No association:<br>Three studies found no association with response.<br><br>Padyukov et al: n=123. P=0.33.[56]<br><br>Guis et al: n=86 P>0.05.[88]<br><br>Jancic et al: n=73 P=0.40[51]	Association:<br>Two studies found an association with response:<br><br>Maxwel et al: n= 1050<br>P=0.001.[83]<br><br>Seitz et al: n=54<br>P<0.0001[89]<br><br>No association:<br>Three studies found no association with response.<br><br>Swierkot et al: n=278. P=NS[81]<br><br>Sode et al: n=538<br>OR (95% CI): 0.96(0.63-1.45). P=0.841.[48]<br><br>Vasilopoulos et al: n=100. P=1. [82]	Association:<br>No studies<br><br>No association:<br>Two studies found no association with response.<br><br>Guseva et al: n=53.[23]<br><br>Daïena et al: n=61. P= 0.332.[58]		Association:<br>No studies<br><br>No association:<br>One study with 80 patients found no association with response.<br>OR (95% CI) = 1.47 (0.51 - 4.30)<br>P= NS.[60]	

TNFAIP3<br>(rs2230926)<br>T/G	chr6:137874929	Association:<br>One study with 291 patients found an association with response.<br>OR (95% CI): 5.395 (1.727–16.86).<br>P=0.0037[13]<br><br>No association:<br>No studies							
TNFAIP3<br>(rs675520)<br>A/G	chr6:137672095					Association:<br>One study with 53 patients found an association with worse results.<br>P=0.01[23]<br><br>No association:<br>No studies			
TNFR1A<br>(rs767455)<br>A/G	chr12:6341779		Association:<br>No studies<br><br>No association:<br>One study with 58 patients found no association with response. P=NS[90]		Association:<br>Two studies found an association with response[9181]<br><br>Morales-Lara et al: n=90. P=0.042[91]<br><br>Swierkot et al: n=280. P=0.011[81]<br><br>No association:<br>No studies				
TNFR2 (rs520916)<br>A>G	chr1:12164396				Association:<br>One study with 979 patients found an association with response.				

					P=0.04[92]<br><br>No association:<br>No studies				
TNFRSF1B (rs1061622) T/G	chr1:12192898		Association:<br>One study with 148 patients found an association with lower response. OR (95% CI) =3.8 (1.30- 11.44), P=0.006[38]<br><br>No association:<br>One study with 58 patients found no association with response. P=NS[90]		Association:<br>Three studies found an association with response[93,949557]:<br><br>Yves-Marie et al: n=15. P=0.017.[57]<br><br>Canet et al: n=471 OR (95% CI): 3.84 (1.45–10.22) P=0.0085.[95]<br><br>Ongaro et al: n=105. OR (95% CI): 2.94 (1.15–7.56). [93]<br><br>Fabris et al: n=66. OR (95% CI): 5.1 (1.3–19.96) P=0.03.[94]<br><br>No association:<br>Three studies found no association with response<br><br>Toonen et al: n=234 P=0.22[96]				

					Swierkot et al:<br>n=280<br>P=NS.[81]<br><br>Vasilopoulos et al:<br>n=100<br>P=0.459.[82]				
TNFRSF11B (rs1485286) C/T	chr8:118938429	Association:<br>One study with 291 patients found an association with response.<br>OR (95% CI): 1.52 (1.026–2.252). P=0.037.[13]<br><br>No association:<br>No studies							
TNFRSF11B (rs2073617) G/A	chr8:118952044	Association:<br>One study with 239 patients found an association with response.<br>OR (95% CI): 1.558. (1.005–2.415). P=0.047[13]<br><br>No association:<br>No studies							
TNFRSF1A (rs4149570) G/T	chr12:6342424			Association:<br>One study with 166 patients found an association with response.<br>OR (95% CI): 0.40(0.18-0.89).	Association:<br>One study with 389 patients found an association with response.<br>OR (95% CI): 0.59 (0.36 - 0.98). P=0.040[48]				

				P=0.025[48]<br><br>No association:<br>No studies	No association:<br>No studies				
TNFRSF1B (rs1061631) G/A	chr1:12208442				Association:<br>One study with 471 patients found an association with response.<br>OR (95% CI): 2.94 (1.16–7.46).<br>P=0.028.[95]<br><br>No association:<br>No studies				
TNFRSF1B (rs3397) C/T	chr1:12207235				Association:<br>One study with 471 patients found an association with response.<br>OR (95% CI): 1.55 (1.08-2.22)<br>P=0.018[95]<br><br>No association:<br>No studies				
TNFSF13B (rs9514828) C/T	chr13:108269025					Association:<br>One study with 115 patients found an association with response .<br>OR (95 CI%): 4.1 (1.3-12.7)<br>P=0.017[97]<br><br>No association:<br>One study with 269 patients (First series of patients:			

						n=152. Replication series of patients: n=117) found no association with response. P=NS.[98]			
TRAF1/C5 (rs3761847) G/A	chr9:12092796				Association:<br>Two studies found an association with response and non-response<br><br>Canhao et al:<br>n=383<br>OR (95% CI):<br>0.61 (0.41- 0.92)<br>P=0.018[71]<br><br>Nishimoto et al:<br>n=101<br>OR (95%CI):<br>16.9 (6.7–41.7).<br>P<0.001.[99]<br><br>No association:<br>No studies				
TRAF3IP2 (rs33980500) C/T	chr6:111592059	Association:<br>One study with 34 patients found an association with response.<br>OR (95% CI): 0.14 (0.02-0.93)<br>P=0.027.[72]<br><br>No association:<br>No studies		Association:<br>No studies<br><br>No association:<br>One study with 100 patients found no association with response.<br>OR (95% CI): 0.78 (0.24-2.55)<br>P=0.68[72]					
TRAILR1 (rs20575) C/G	chr8:23201811		Association:<br>One study with 75 patients found an		Association:<br>One study with 90 patients found an				

			association with response.<br>P=0.03[91]<br><br>No association:<br>No studies		association with response.<br>P=0.019[91]<br><br>No association:<br>No studies				
VEGFA<br>(rs25648)<br>C/T	chr6:43771240	Association:<br>One study with 202 patients found an association with response.<br>OR (95% CI): 2.571 (1.354–4.883).<br>P=0.0039[13]<br><br>No association:<br>No studies							
VWF<br>(rs216897)<br>T/C	chr12:5993441	Association:<br>One study with 291 patients found an association with response.<br>OR (95% CI): 1.52 (1.057–2.175).<br>P=0.024[13]<br><br>No association:<br>No studies							
WDR27<br>(rs75908454)<br>T/C	chr6:169570527				Association:<br>One study with 444 patients found an association with response.<br>P=6.3x10^{-7}.[47]<br><br>No association:<br>No studies				

WDR49 (rs4552347) C/A	chr3:167569890			Association: One study with 134 patients found an association with secondary failure. OR: 4.765. P= 7.45 x 10^{-6}.[5] No association: No studies					
ZNF180 (rs17714261) C/T	chr19:44473045				Association: One study with 196 patients found an association with being non-responder. OR (95% CI): 0.3 (0.16 - 0.53) P= 2.00 x 10^{-5}.[3] No association: No studies				
ZNF214 (rs6578820) A/G	chr11:7016598				Association: One study with 196 patients found an association with non-response. OR (95% CI): 0.3 (0.18, 0.55). P=2.70 x 10^{-5}.[3] No association: No studies				
ZNF595,ZNF718. (rs2187874) G/T	chr4:82429				Association: One study with 1723 patients found an				

					association with response.<br>P= 7.00 x 10^{-8}. [7]** <br>No association:<br>No studies				
ZNF618<br>(rs16911006)<br>G/A	chr9:113962122				Association:<br>One study with 196 patients found an association with response.<br>OR (95% CI): 0.33 (0.2, 0.56).<br>P=2.80E -05[3]<br><br>No association:<br>No studies				

*No differentiation on the individual TNFi
** Genome wide significance. ($p < 5 \times 10^{-8}$) not reached
*** Significance with a P-value less than or equal to 0.003 (corresponding to a Bonferroni corrected P-value equal to 5%) not reached.
#Different minor allele than National Center of Biotechnology Information in the National Institute of Health (NCBI, NIH) due to use of minor allele in included studies

References

1. Tan RJL, Gibbons LJ, Potter C, et al. Investigation of rheumatoid arthritis susceptibility genes identifies association of AFF3 and CD226 variants with response to anti-tumour necrosis factor treatment. *Ann Rheum Dis*. 2010;69(6):1029-1035. doi:10.1136/ard.2009.118406
2. Zervou MI, Myrthianou E, Flouri I, et al. Lack of Association of Variants Previously Associated with Anti-TNF Medication Response in Rheumatoid Arthritis Patients: Results from a Homogeneous Greek Population. *PLoS One*. 2013;8(9):1-5. doi:10.1371/journal.pone.0074375
3. Krintel SB, Palermo G, Johansen JS, et al. Investigation of single nucleotide polymorphisms and biological pathways associated with response to TNFα inhibitors in patients with rheumatoid arthritis. *Pharmacogenet Genomics*. 2012;22(8):577-589. doi:10.1097/FPC.0b013e3283544043
4. Lopez-Rodriguez R, Perez-Pampin E, Marquez A, et al. Validation study of genetic biomarkers of response to TNF inhibitors in rheumatoid arthritis. *PLoS One*. 2018;13(5):1-13. doi:10.1371/journal.pone.0196793
5. Funahashi K, Koyano S, Echizen H, Matsubara T. Whole genome analysis on the genetic backgrounds associated with the secondary failure to etanercept in patients with rheumatoid arthritis. *Mod Rheumatol*. 2017;27(2):271-277. doi:10.1080/14397595.2016.1206172
6. Juli A, Fernandez-Nebro A, Blanco F, et al. A genome-wide association study identifies a new locus associated with the response to anti-TNF therapy in rheumatoid arthritis. *Pharmacogenomics J*. 2016;16(2):147-150. doi:10.1038/tpj.2015.31
7. Massey J, Plant D, Hyrich K, et al. Genome-wide association study of response to tumour necrosis factor inhibitor therapy in rheumatoid arthritis. *Pharmacogenomics J*.

2018;18(5):657-664. doi:10.1038/s41397-018-0040-6
8. Liu C, Batliwalla F, Li W, et al. Genome-wide association scan identifies candidate polymorphisms associated with differential response to anti-TNF treatment in rheumatoid arthritis. *Mol Med*. 2008;14(9-10):575-581. doi:10.2119/2008-00056.Liu
9. Suarez-Gestal M, Perez-Pampin E, Calaza M, Gomez-Reino JJ, Gonzalez A. Lack of replication of genetic predictors for the rheumatoid arthritis response to anti-TNF treatments: A prospective case-only study. *Arthritis Res Ther*. 2010;12(2). doi:10.1186/ar2990
10. Maša Umićević Mirkov, Jing Cui, Sita H Vermeulen, Eli A. Stahl, Erik JM Toonen, Remco R Makkinje, Annette T Lee, Tom WJ Huizinga, Renee Allaart, Anne Barton, Xavier Mariette, Corinne Miceli-Richard, Lindsey A Criswell, Paul P Tak,#, Niek and MJC. Genome-wide association analysis of anti-TNF drug response in rheumatoid arthritis patients. *Ann Rheum Dis*. 2013;72(8):1375-1381. doi:10.1136/annrheumdis-2012-202405.Genome-wide
11. Mathews RJ, Robinson JI, Battellino M, et al. Evidence of NLRP3-inflammasome activation in rheumatoid arthritis (RA); Genetic variants within the NLRP3-inflammasome complex in relation to susceptibility to RA and response to anti-TNF treatment. *Ann Rheum Dis*. 2014;73(6):1202-1210. doi:10.1136/annrheumdis-2013-203276
12. Canet LM, Cáliz R, Lupiañez CB, et al. Genetic variants within immune-modulating genes influence the risk of developing rheumatoid arthritis and anti-TNF drug response: A two-stage case-control study. *Pharmacogenet Genomics*. 2015;25(9):432-443. doi:10.1097/FPC.0000000000000155
13. Eektimmerman F, Swen J, Böhringer S, Huizinga TWJ, Wouter M. Pathway analysis to identify genetic variants associated with efficacy of adalimumab in rheumatoid arthritis. *Pharmacogenomics*. 2017;18(10):945-953.
14. Cui J, Saevarsdottir S, Thomson B, et al. Rheumatoid arthritis risk allele PTPRC is also associated with response to anti-tumor necrosis factor α therapy. *Arthritis Rheum*. 2010;62(7):1849-1861. doi:10.1002/art.27457
15. Maldonado-Montoro M, Cañadas-Garre M, González-Utrilla A, Plaza-Plaza JC, Calleja-Hernández MÁ. Genetic and clinical biomarkers of tocilizumab response in patients with rheumatoid arthritis. *Pharmacol Res*. 2016;111:264-271. doi:10.1016/j.phrs.2016.06.016
16. Wang J, Bansal AT, Martin M, et al. Genome-wide association analysis implicates the involvement of eight loci with response to tocilizumab for the treatment of rheumatoid arthritis. *Pharmacogenomics J*. 2013;13(3):235-241. doi:10.1038/tpj.2012.8
17. Diaz-Villamarin, Xando; LuciaDavila-Fajardo, Cristina; Blanquez-Martinez, David; Caballero-Romero, Alvaro; Perez-Campos, Manuel; Nieto-Gomez, Pelayo; Cabeza-Barrera, Jose; Gonzalez-Medina, Maica; Antunez-Rodriguez, Alba; Martinez-Gonzalez LJ. Genetic polymorphisms influence on the response to tocilizumab. *Int J Clin Pharm*. 2018;40(1):257.
18. Iwaszko M, Świerkot J, Kolossa K, Jeka S, Wiland P, Bogunia-Kubik K. Influence of CD94 and NKG2A variants on susceptibility to rheumatoid arthritis and efficacy of anti-TNF treatment. *Jt Bone Spine*. 2016;83(1):75-79. doi:10.1016/j.jbspin.2015.06.010
19. Potter C, Cordell HJ, Barton A, et al. Association between anti-tumour necrosis factor treatment response and genetic variants within the TLR and NFκB signalling pathways. *Ann Rheum Dis*. 2010;69(7):1315-1320. doi:10.1136/ard.2009.117309
20. Ferreiro-Iglesias A, Montes A, Perez-Pampin E, et al. Replication of PTPRC as genetic biomarker of response to TNF inhibitors in patients with rheumatoid arthritis. *Pharmacogenomics J*. 2016;16(2):137-140. doi:10.1038/tpj.2015.29
21. Sode J, Vogel U, Bank S, et al. Confirmation of an IRAK3 polymorphism as a genetic marker predicting response to anti-TNF treatment in rheumatoid arthritis. *Pharmacogenomics J*. 2018;18(1):81-86. doi:10.1038/tpj.2016.66
22. Pete NM, Montoro MDMM, Ramírez CP, et al. Impact of single-nucleotide polymorphisms of CTLA-4, CD80 and CD86 on the effectiveness of abatacept in patients with rheumatoid arthritis. *J Pers Med*. 2020;10(4):1-17. doi:10.3390/jpm10040220
23. Guseva, I.; Soroka, N.; Trofimov, D.; Devyataykina, A.; Lukina, G.; Aleksandrova, E.; Novikov, A.; Glukhova, S.; Nasonov E. TNFAIP3 RS675520 variant may predict response to rituximab treatment in rheumatoid arthritis. *Ann Rheum Dis*. 2013;71((Suppl 3)):669.
24. Canet LM, Sánchez-Maldonado JM, Cáliz R, et al. Polymorphisms at phase I-metabolizing enzyme and hormone receptor loci influence the response to anti-TNF therapy in rheumatoid arthritis patients. *Pharmacogenomics J*. 2019;19(1):83-96. doi:10.1038/s41397-018-0057-x
25. Avila-Pedretti G, Tornero J, Fernández-Nebro A, et al. Variation at FCGR2A and functionally related genes is associated with the response to anti-TNF therapy in rheumatoid arthritis. *PLoS One*. 2015;10(4):1-12. doi:10.1371/journal.pone.0122088
26. Jiang X, Askling J, Saevarsdottir S, et al. A genetic risk score composed of rheumatoid arthritis risk alleles, HLA-DRB1 haplotypes, and response to TNFi therapy - results from a Swedish cohort study. *Arthritis Res Ther*. 2016;18(1):1-10. doi:10.1186/s13075-016-1174-z
27. Plant D, Bowes J, Potter C, et al. Genome-wide association study of genetic predictors of anti-tumor necrosis factor treatment efficacy in rheumatoid arthritis identifies associations

with polymorphisms at seven loci. *Arthritis Rheum*. 2011;63(3):645-653. doi:10.1002/art.30130
28. Márquez A, Ferreiro-Iglesias A, Dávila-Fajardo CL, et al. Lack of validation of genetic variants associated with anti-tumor necrosis factor therapy response in rheumatoid arthritis: A genome-wide association study replication and meta-analysis. *Arthritis Res Ther*. 2014;16(2):1-7. doi:10.1186/ar4504
29. Dávila- CL, Huizinga W, Dávila-Fajardo CL, et al. FcGR genetic polymorphisms and the response to adalimumab in patients with rheumatoid arthritis. *Pharmacogenomics*. 2015;16(4):373-381. https://pubmed.ncbi.nlm.nih.gov/25823785/
30. Montes A, Perez-Pampin E, Narváez J, et al. Association of FCGR2A with the response to infliximab treatment of patients with rheumatoid arthritis. *Pharmacogenet Genomics*. 2014;24(5):238-245. doi:10.1097/FPC.0000000000000042
31. Cañete JD, Suárez B, Hernández M V., et al. Influence of variants of Fcγ receptors IIA and IIIA on the American College of Rheumatology and European League Against Rheumatism responses to anti-tumour necrosis factor α therapy in rheumatoid arthritis. *Ann Rheum Dis*. 2009;68(10):1547-1552. doi:10.1136/ard.2008.096982
32. Jiménez Morales A, Maldonado-Montoro M, Martínez de la Plata JE, et al. FCGR2A/FCGR3A Gene Polymorphisms and Clinical Variables as Predictors of Response to Tocilizumab and Rituximab in Patients With Rheumatoid Arthritis. *J Clin Pharmacol*. 2019;59(4):517-531. doi:10.1002/jcph.1341
33. MDC González-Medina, CL Dávila-Fajardo, MJ Soto-Pino, X Díaz-Villamarín, A Gómez- Martín, LJ Martínez-González, M Núñez, I Casas-Hidalgo JC-B. The FCGR2A (A >G) (Rs1801274) Genetic variant and the efficacy of Tocilizumab in rheumatoid arthritis patients. *Eur J Hosp Pharm*. 2016;23(Suppl 1):A189.2-A190. doi:10.1136/ejhpharm-2016-000875.428
34. Luxembourger C, Ruyssen-Witrand A, Ladhari C, et al. A single nucleotide polymorphism of IL6-receptor is associated with response to tocilizumab in rheumatoid arthritis patients. *Pharmacogenomics J*. 2019;19(4):368-374. doi:10.1038/s41397-019-0072-6
35. Márquez Pete N, Maldonado Montoro MDM, Pérez Ramírez C, et al. Influence of the fcgr2a rs1801274 and fcgr3a rs396991 polymorphisms on response to abatacept in patients with rheumatoid arthritis. *J Pers Med*. 2021;11(6):1-15. doi:10.3390/jpm11060573
36. A. M, E. P-P, B. J, et al. FCGR polymorphisms in the treatment of rheumatoid arthritis with Fc-containing TNF inhibitors. *Pharmacogenomics*. 2015;16(4):333-345. http://www.futuremedicine.com/loi/pgs?cookieSet=1%5Cnhttp://ovidsp.ovid.com/ovidweb.cgi?T=JS&PAGE=reference&D=emed13&NEWS=N&AN=2015881019
37. Morales-Lara MJ, Conesa-Zamora P, Garca-Simón MS, et al. Association between the FCGR3A V158F polymorphism and the clinical response to infliximab in rheumatoid arthritis and spondyloarthritis patients. *Scand J Rheumatol*. 2010;39(6):518-520. doi:10.3109/03009741003781969
38. Rooryck C, Barnetche T, Richez C, Laleye A, Arveiler B, Schaeverbeke T. Influence of FCGR3A-V212F and TNFRSF1B-M196R genotypes in patients with rheumatoid arthritis treated with infliximab therapy. *Clin Exp Rheumatol*. 2008;26(2):340-342.
39. Kastbom A, Bratt J, Ernestam S, et al. Fcγ receptor type III A genotype and response to tumor necrosis factor α-blocking agents in patients with rheumatoid arthritis. *Arthritis Rheum*. 2007;56(2):448-452. doi:10.1002/art.22390
40. Quartuccio L, Fabris M, Pontarini E, et al. The 158VV Fcgamma receptor 3A genotype is associated with response to rituximab in rheumatoid arthritis: Results of an italian multicentre study. *Ann Rheum Dis*. 2014;73(4):716-721. doi:10.1136/annrheumdis-2012-202435
41. Ruyssen-Witrand A, Rouanet S, Combe B, et al. Fcγ receptor type IIIA polymorphism influences treatment outcomes in patients with rheumatoid arthritis treated with rituximab. *Ann Rheum Dis*. 2012;71(6):875-877. doi:10.1136/annrheumdis-2011-200337
42. Kastbom A, Cöster L, Ärlestig L, et al. Influence of FCGR3A genotype on the therapeutic response to rituximab in rheumatoid arthritis: An observational cohort study. *BMJ Open*. 2012;2(5):1-4. doi:10.1136/bmjopen-2012-001524
43. Pál I, Szamosi S, Hodosi K, Szekanecz Z, Váróczy L. Effect of Fcγ-receptor 3a (FCGR3A) gene polymorphisms on rituximab therapy in Hungarian patients with rheumatoid arthritis. *RMD Open*. 2017;3(2):1-4. doi:10.1136/rmdopen-2017-000485
44. Sarsour K, Greenberg J, Johnston JA, et al. The role of the FcGRIIIa polymorphism in modifying the association between treatment and outcome in patients with rheumatoid arthritis treated with rituximab versus TNF-α antagonist therapies. *Clin Exp Rheumatol*. 2013;31(2):0189-0194.
45. Gazeau P, Alegria GC, Devauchelle-Pensec V, et al. Memory B Cells and Response to Abatacept in Rheumatoid Arthritis. *Clin Rev Allergy Immunol*. 2017;53(2):166-176. doi:10.1007/s12016-017-8603-x
46. Montes A, Perez-Pampin E, Navarro-Sarabia F, et al. Rheumatoid arthritis response to treatment across IgG1 allotype - anti-TNF incompatibility: A case-only study. *Arthritis Res Ther*. 2015;17(1):1-12. doi:10.1186/s13075-015-0571-z
47. Honne K, Hallgrímsdóttir I, Wu C, et al. A longitudinal genome-wide association study of anti-tumor necrosis factor response among Japanese patients with rheumatoid arthritis. *Arthritis Res Ther*. 2016;18(1):1-10. doi:10.1186/s13075-016-0920-6

48. Sode J, Vogel U, Bank S, et al. Anti-TNF treatment response in rheumatoid arthritis patients is associated with genetic variation in the NLRP3-inflammasome. *PLoS One*. 2014;9(6):1-10. doi:10.1371/journal.pone.0100361
49. Sode J, Vogel U, Bank S, et al. Genetic variations in pattern recognition receptor loci are associated with anti-TNF response in patients with rheumatoid arthritis. *PLoS One*. 2015;10(10):1-13. doi:10.1371/journal.pone.0139781
50. Hassan B, Maxwell JR, Hyrich KL, et al. Genotype at the sIL-6R A358C polymorphism does not influence response to anti-TNF therapy in patients with rheumatoid arthritis. *Rheumatology (Oxford)*. 2010;49(1):43-47. doi:10.1093/rheumatology/kep372
51. Jančić I, Šefik-Bukilica M, Živojinović S, et al. Influence of Promoter Polymorphisms of the Tnf-α (-308g/A) and IL-6 (-174g/C) Genes on Therapeutic Response to Etanercept in Rheumatoid Arthritis. *J Med Biochem*. 2015;34(4):414-421. doi:10.2478/jomb-2014-0060
52. Jančić I, Arsenović-Ranin N, Šefik-Bukilica M, et al. -174G/C interleukin-6 gene promoter polymorphism predicts therapeutic response to etanercept in rheumatoid arthritis. *Rheumatol Int*. 2013;33(6):1481-1486. doi:10.1007/s00296-012-2586-y
53. Dávila-Fajardo CL, Márquez A, Pascual-Salcedo D, et al. Confirmation of -174G/C interleukin-6 gene promoter polymorphism as a genetic marker predicting antitumor necrosis factor treatment outcome. *Pharmacogenet Genomics*. 2014;24(1):1-5. doi:10.1097/FPC.0000000000000013
54. Fabris M, Quartuccio L, Lombardi S, et al. Study on the possible role of the -174G>C IL-6 promoter polymorphism in predicting response to rituximab in rheumatoid arthritis. *Reumatismo*. 2011;62(4):253-258. doi:10.4081/reumatismo.2010.253
55. M. DCG-M, C.L. D-F, X. D-V, L.J. M-G, P. M-R. The IL-6 G>C genetic polymorphism (rs1800795) on the response to Tocilizumab at 3, 6, 9 and 12 months. *Int J Clin Pharm*. 2017;39(1):337. http://ovidsp.ovid.com/ovidweb.cgi?T=JS&PAGE=reference&D=emed18&NEWS=N&AN=614479082
56. Padyukov L, Lampa J, Heimbürger M, et al. Genetic markers for the efficacy of tumour necrosis factor blocking therapy in rheumatoid arthritis. *Ann Rheum Dis*. 2003;62(6):526-529. doi:10.1136/ard.62.6.526
57. Pers YM, Cadart D, Rittore C, et al. TNFRII polymorphism is associated with response to TNF blockers in rheumatoid arthritis patients seronegative for ACPA. *Jt Bone Spine*. 2014;81(4):370-372. doi:10.1016/j.jbspin.2013.12.005
58. Daïen CI, Fabre S, Rittore C, et al. TGF beta1 polymorphisms are candidate predictors of the clinical response to rituximab in rheumatoid arthritis. *Jt Bone Spine*. 2012;79(5):471-475. doi:10.1016/j.jbspin.2011.10.007
59. Schotte H, Schlüter B, Schmidt H, et al. Putative IL-10 low producer genotypes are associated with a favourable etanercept response in patients with rheumatoid arthritis. *PLoS One*. 2015;10(6):1-13. doi:10.1371/journal.pone.0130907
60. Camp NJ, Cox A, di Giovine FS, McCabe D, Rich W, Duff GW. Evidence of a pharmacogenomic response to interleukin-1 receptor antagonist in rheumatoid arthritis. *Genes Immun*. 2005;6(6):467-471. doi:10.1038/sj.gene.6364228
61. Plant D, Prajapati R, Hyrich KL, et al. Replication of association of the PTPRC gene with response to anti-tumor necrosis factor therapy in a large UK cohort. *Arthritis Rheum*. 2012;64(3):665-670. doi:10.1002/art.33381
62. Enevold C, Baslund B, Linde L, et al. Interleukin-6-receptor polymorphisms rs12083537, rs2228145, and rs4329505 as predictors of response to tocilizumab in rheumatoid arthritis. *Pharmacogenet Genomics*. 2014;24(8):401-405. doi:10.1097/FPC.0000000000000071
63. Maldonado-Montoro M, Cañadas-Garre M, González-Utrilla A, Ángel Calleja-Hernández M. Influence of IL6R gene polymorphisms in the effectiveness to treatment with tocilizumab in rheumatoid arthritis. *Pharmacogenomics J*. 2018;18(1):167-172. doi:10.1038/tpj.2016.88
64. Juge PA, Gazal S, Constantin A, et al. Variants of genes implicated in type 1 interferon pathway and B-cell activation modulate the EULAR response to rituximab at 24 weeks in rheumatoid arthritis. *RMD Open*. 2017;3(2):1-5. doi:10.1136/rmdopen-2017-000448
65. X Díaz-Villamarín, CL Dávila-Fajardo, MDC Gónzalez-Medina, MJ Soto-Pino, E Sánche-Gómez, A Acuña, A Gómez-Martín, LJ Martínez-González, I Casas-Hidalgo JC-, Barrera. The KCNMB1 (A >G) (RS703505) genetic variant and the efficacy of tocilizumab in rheumatoid arthritis patients. *Eur J Hosp Pharm*. 2016;23(Suppl 1):A188.1-A188. doi:10.1136/ejhpharm-2016-000875.424
66. Coulthard LR, Taylor JC, Eyre S, et al. Genetic variants within the MAP kinase signaling network and anti-TNF treatment response in Rheumatoid arthritis patients. *Ann Rheum Dis*. 2011;70(1):98-103. doi:10.1136/ard.2010.133249
67. Bowes JD, Potter C, Gibbons LJ, et al. Investigation of genetic variants within candidate genes of the TNFRSF1B signalling pathway on the response to anti-TNF agents in a UK cohort of rheumatoid arthritis patients. *Pharmacogenet Genomics*. 2009;19(4):319-323. doi:10.1097/FPC.0b013e328328d51f

68. Juli A, Fernandez-Nebro A, Blanco F, et al. A genome-wide association study identifies a new locus associated with the response to anti-TNF therapy in rheumatoid arthritis. *Pharmacogenomics J*. 2016;16(2):147-150. doi:10.1038/tpj.2015.31
69. Drynda, S.; Kekow, J.; Glotzner, M.; Leesch D. Prediction of therapy response of an anti-TNFalpha- and a B-cell directed therapy in patients with rheumatoid arthritis based on a discrete single nucleotide polymorphism in the MED29 gene. *Z Rheumatol*. 2012;71(0):109.
70. Iwaszko M, Świerkot J, Kolossa K, Jeka S, Wiland P, Bogunia-Kubik K. Influence of NKG2D genetic variants on response to anti-TNF agents in patients with rheumatoid arthritis. *Genes (Basel)*. 2018;9(2):1-15. doi:10.3390/genes9020064
71. Canhão H, Rodrigues AM, Santos MJ, et al. TRAF1/C5 but not PTPRC variants are potential predictors of rheumatoid arthritis response to anti-tumor necrosis factor therapy. *Biomed Res Int*. 2015;2015. doi:10.1155/2015/490295
72. Conigliaro P, Ciccacci C, Politi C, et al. Polymorphisms in STAT4, PTPN2, PSORS1C1 and TRAF3IP2 genes are associated with the response to TNF inhibitors in patients with rheumatoid arthritis. *PLoS One*. 2017;12(1):1-14. doi:10.1371/journal.pone.0169956
73. I. Acosta-Colman, N. Palau, J. Tornero, A. Fernández-Nebro, F. Blanco, I. González-Alvaro, J. D Cañete, J. Maymó, J. Ballina, B.Fernández-Gutiérrez, A.Olivé, H. Corominas, A. Erra, O. Canela-Xandri, Ar AJ& SM. GWAS replication study confirms the association of PDE3A–SLCO1C1 with anti-TNF therapy response in rheumatoid arthritis. *Pharmacogenomics*. 2013;14(7):727-734.
74. Smith SL, Plant D, Lee XH, et al. Previously reported PDE3A-SLCO1C1 genetic variant does not correlate with anti-TNF response in a large UK rheumatoid arthritis cohort. *Pharmacogenomics*. 2016;17(7):715-720. doi:10.2217/pgs.16.16
75. G. Ozen, B. Saglam, A. Odabasi, O. Ozluk, S.P. Yentur, G. Saruhan-Direskeneli, N. Inanc HD. STAT4 Rs7574865 gene polymorphism is not associated with severe disease phenotype and response to tumor necrosis factor-α inhibitor treatment in patients with rheumatoid arthritis. *Ann Rheum Dis*. 2015;74(Suppl 2):979.2-980. doi:10.1136/annrheumdis-2015-eular.5901
76. Potter C, Hyrich KL, Tracey A, et al. Association of rheumatoid factor and anti-cyclic citrullinated peptide positivity, but not carriage of shared epitope or PTPN22 susceptibility variants, with anti-tumour necrosis factor response in rheumatoid arthritis. *Ann Rheum Dis*. 2009;68(1):69-74. doi:10.1136/ard.2007.084715
77. Gębura K, Świerkot J, Wysoczańska B, et al. Polymorphisms within genes involved in regulation of the NF-κB pathway in patients with rheumatoid arthritis. *Int J Mol Sci*. 2017;18(7):1-11. doi:10.3390/ijms18071432
78. Miceli-Richard C, Comets E, Verstuyft C, et al. A single tumour necrosis factor haplotype influences the response to adalimumab in rheumatoid arthritis. *Ann Rheum Dis*. 2008;67(4):478-484. doi:10.1136/ard.2007.074104
79. Shkaruba, N.; Silkov, A.; Sennikova, J.; Sizikov, A.; Herzsog, O.; Kozlov, V.; Sennikov, S.; Dolgikh, S.; Mazurov, V.; Shulman, J.; Sizyakina, L.; Kalashnikova T. Single nucleotide polymorphisms in the TNF-alpha gene and efficacy of anticytokine therapy in patients with rheumatoid arthritis. *Ann Rheum Dis*. 2013;71(0):Suppl 3.
80. Kang CP, Lee KW, Yoo DH, Kang C, Bae SC. The influence of a polymorphism at position -857 of the tumour necrosis factor α gene on clinical response to etanercept therapy in rheumatoid arthritis. *Rheumatology*. 2005;44(4):547-552. doi:10.1093/rheumatology/keh550
81. Swierkot J, Bogunia-Kubik K, Nowak B, et al. Analysis of associations between polymorphisms within genes coding for tumour necrosis factor (TNF)-alpha and TNF receptors and responsiveness to TNF-alpha blockers in patients with rheumatoid arthritis. *Jt Bone Spine*. 2015;82(2):94-99. doi:10.1016/j.jbspin.2014.08.006
82. Vasilopoulos Y, Bagiatis V, Stamatopoulou D, et al. Association of anti-CCP positivity and carriage of TNFRII susceptibility variant with anti-TNF-α response in rheumatoid arthritis. *Clin Exp Rheumatol*. 2011;29(4):701-704.
83. Maxwel JR, Potter C, Hyrich KL, et al. Association of the tumour necrosis factor-308 variant with differential response to anti-TNF agents in the treatment of rheumatoid arthritis. *Hum Mol Genet*. 2008;17(22):3532-3538. doi:10.1093/hmg/ddn245
84. Pinto JA, Rego I, Fernandez-López C, et al. Polymorphisms in genes encoding tumor necrosis factor-α and HLA-DRB1 are not associated with response to infliximab in patients with rheumatoid arthritis (Journal of Rheumatology (2008) 35, (177-178)). *J Rheumatol*. 2008;35(3):546.
85. Cuchacovich M, Soto L, Edwardes M, et al. Tumour necrosis factor (TNF)α -308 G/G promoter polymorphism and TNFα levels correlate with a better response to adalimumab in patients with rheumatoid arthritis. *Scand J Rheumatol*. 2006;35(6):435-440. doi:10.1080/03009740600904284
86. Mugnier B, Balandraud N, Darque A, Roudier C, Roudier J, Reviron D. Polymorphism at position -308 of the tumor necrosis factor α gene influences outcome of infliximab therapy in rheumatoid arthritis. *Arthritis Rheum*. 2003;48(7):1849-1852. doi:10.1002/art.11168
87. Marotte H, Arnaud B, Diasparra J, Zrioual S, Miossec P. Association between the level of circulating bioactive tumor necrosis factor α and the tumor necrosis factor α gene

polymorphism at -308 in patients with rheumatoid arthritis treated with a tumor necrosis factor α inhibitor. *Arthritis Rheum*. 2008;58(5):1258-1263. doi:10.1002/art.23430
88. Guis S, Balandraud N, Bouvenot J, et al. Influence of -308 A/G polymorphism in the tumor necrosis factor α gene on etanercept treatment in rheumatoid arthritis. *Arthritis Care Res*. 2007;57(8):1426-1430. doi:10.1002/art.23092
89. Seitz M, Wirthmüller U, Möller B, Villiger PM. The -308 tumour necrosis factor-α gene polymorphism predicts therapeutic response to TNFα-blockers in rheumatoid arthritis and spondyloarthritis patients. *Rheumatology*. 2007;46(1):93-96. doi:10.1093/rheumatology/kel175
90. Chatzikyriakidou A, Georgiou I, Voulgari P V., Venetsanopoulou AI, Drosos AA. Combined tumour necrosis factor-α and tumour necrosis factor receptor genotypes could predict rheumatoid arthritis patients' response to anti-TNF-α therapy and explain controversies of studies based on a single polymorphism [1]. *Rheumatology*. 2007;46(6):1034-1035. doi:10.1093/rheumatology/kem041
91. Morales-Lara MJ, Cañete JD, Torres-Moreno D, et al. Effects of polymorphisms in TRAILR1 and TNFR1A on the response to anti-TNF therapies in patients with rheumatoid and psoriatic arthritis. *Jt Bone Spine*. 2012;79(6):591-596. doi:10.1016/j.jbspin.2012.02.003
92. Potter C, Gibbons LJ, Bowes JD, et al. Polymorphisms spanning the TNFR2 and TACE genes do not contribute towards variable anti-TNF treatment response. *Pharmacogenet Genomics*. 2010;20(5):338-341. doi:10.1097/FPC.0b013e32833878d7
93. Ongaro A, De Mattei M, Pellati A, et al. Can tumor necrosis factor receptor II gene 676T>G polymorphism predict the response grading to anti-TNFα therapy in rheumatoid arthritis? *Rheumatol Int*. 2008;28(9):901-908. doi:10.1007/s00296-008-0552-5
94. Fabris M, Tolusso B, Di Poi E, Assaloni R, Sinigaglia L, Ferraccioli G. Tumor necrosis factor-α receptor II polymorphism in patients from Southern Europe with mild-moderate and severe rheumatoid arthritis. *J Rheumatol*. 2002;29(9):1847-1850.
95. Canet LM, Filipescu I, Caliz R, et al. Genetic variants within the TNFRSF1B gene and susceptibility to rheumatoid arthritis and response to anti-TNF drugs: A multicenter study. *Pharmacogenet Genomics*. 2015;25(7):323-333. doi:10.1097/FPC.0000000000000140
96. Toonen EJM, Coenen MJH, Kievit W, et al. The tumour necrosis factor receptor superfamily member 1b 676T>G polymorphism in relation to response to infliximab and adalimumab treatment and disease severity in rheumatoid arthritis. *Ann Rheum Dis*. 2008;67(8):1174-1177. doi:10.1136/ard.2008.088138
97. Ruyssen-Witrand A, Rouanet S, Combe B, et al. Association between -871C>T promoter polymorphism in the B-cell activating factor gene and the response to rituximab in rheumatoid arthritis patients. *Rheumatol (United Kingdom)*. 2013;52(4):636-641. doi:10.1093/rheumatology/kes344
98. Fabris M, Quartuccio L, Vital E, et al. The TTTT B lymphocyte stimulator promoter haplotype is associated with good response to rituximab therapy in seropositive rheumatoid arthritis resistant to tumor necrosis factor blockers. *Arthritis Rheum*. 2013;65(1):88-97. doi:10.1002/art.37707
99. Nishimoto T, Seta N, Anan R, et al. A single nucleotide polymorphism of TRAF1 predicts the clinical response to anti-TNF treatment in Japanese patients with rheumatoid arthritis. *Clin Exp Rheumatol*. 2014;32(2):211-217.
