# Supplementary material for: The Association between Genetics and Response to Treatment with Biologics in Patients with Psoriasis, Psoriatic Arthritis, Rheumatoid Arthritis, and Inflammatory Bowel Diseases: A Systematic Review and Meta-Analysis
Source: Int J Mol Sci. 2024 May 26;25(11):5793. doi: 10.3390/ijms25115793 (PMC11171831; doi:10.3390/ijms25115793)
Supplement: Supplementary file 1 [file ijms-25-05793-s001.zip › Supplementary Table S5.pdf]

IBD Supplementary Table S5

		Anti-TNF					Anti-IL12/23
Gene (rs number)	Chromosome number:location	Adalimumab	Infliximab	Etanercept	Golimumab	Anti-TNF combined	Ustekinumab
AHR / LINC02888 (rs1077773) G/A	chr7:17403055					Association<br>Two studies found an association with response.<br><br>Yoon et al:<br>n=459<br>OR (95% CI) = 0.61 (0.42–0.88). P=0.008[1]<br><br>Burke et al:<br>n=231<br>OR: 0.68. P=0.035[2]<br><br>No association<br>No studies	
ARFGAP2 (rs3740691) G/A	chr11:47166860	Association<br>One study with 102 patients found an association with response.<br>P=2.74E-04[3]<br><br>No association<br>No studies					
ATG16L1 (rs12994997) G/A	chr2:233264857					Association<br>One study with 359 patients found an association with response.	

						OR= 0.682<br>P=0.047.[4]<br><br>No association<br>No studies	
ATG16L1 (rs2241880) A/G	chr2:233274722	Association<br>No studies<br><br>No association<br>One study with 225 patients found no association with response. P=0.979.[5]	Association<br>One study with 94 patients found an association with response. RR=2.8 (1.0, 8.7). P=0.048.[6]<br><br>No association<br>One study found with 120 patients found no association with response. P=0.931.[5]		Association<br>No studies<br><br>No association<br>One study with 7 patients found no association with response. P=1.00.[5]	Association<br>No studies<br><br>No association<br>One study with 121 patients found no association with response. P=NS.[7]	Association<br>No studies<br><br>No association<br>One study with 109 patients found no association with response. P=0.875.[5]
ATG5 (rs9373839) T/C	chr6:106207742	Association<br>One study with 79 patients found an association with response. $P=5.15x10^{-05}$.[8]<br><br>No association<br>No studies					
ATXN2 (rs653178) C/T	chr12:111569952					Association<br>One study with 231 patients found an association with response.<br>OR: 1.78.	

						P=0.049.[2]<br><br>No association<br>No studies	
ATXN2L (rs35725751) C/T	chr16:28834263					Association<br>One study with 359 patients found an association with response. OR= 0.45 P=9.30E-05.[4]<br><br>No association<br>No studies	
ATXN2L (rs8049439) T/C	chr16:28826194	Association<br>One study with 97 patients found an association with response.<br>P= 2.96E-02.[9]<br><br>No association<br>No studies					
BRWD1 (rs2836878) G/A	chr21:39093608		Association<br>One study with 94 patients found an association with primary response.<br>RR=2.6 (1-6.5).<br>P=0.03.[6]<br><br>No association<br>No studies				
C1orf106 (rs61740234) C/T	chr1:200911574		Association<br>One study with 206 patients found an association				

			with response. OR (95% CI): 4.49 (1.31–15.32). P= 0.010[10]<br><br>No association<br>No studies				
CASP9 (rs4645983) G/A	chr1:15524108	Association<br>One study with 97 patients found an association with response. P=6.91E-03.[9]<br><br>No association<br>No studies	Association<br>One study with 204 patients found an association with response. P=0.04.[11]<br><br>No association<br>One study with 82 patients found no association with response.[12]				
CCDC88B (rs61886887) C/T	chr11:64352979		Association<br>One study with 206 patients found an association with response. OR (95% CI): 0.08 (0.01–0.61). P=0.002[10]<br><br>No association<br>No studies				
CCNY (rs12777960) C/A	chr10:35252098	Association<br>One study with 97 patients found an association with					

		response.<br>OR (95% CI):<br>3.26 (1.27-8.38).<br>P=1.56E-02.[9]<br><br>No association<br>No studies					
CD14 (rs2569190) A/G	chr5:140633331	Association<br>No studies<br><br>No association<br>One study with 24 patients found no association with response. P=NS.[13]				Association<br>One study with 254 patients found an association with response.<br>OR (95% CI): 0.54 (0.30-0.98).<br>P=0.04.[14]<br><br>No association<br>No studies	
CD28 (rs3116494) G/A	chr2:203727298					Association<br>One study with 459 patients found an association with time to loss of response.<br>HR (95% CI) = 1.46 (1.20–1.79).<br>P= 0.0002.[1]<br><br>No association<br>No studies	
CDKAL1 (rs6908425) T/C	chr6:20728500		Association<br>One study with 94 patients found an association with response.				

			RR=2.1 (1.0-4.3). P=0.049.[6]<br><br>No association<br>No studies				
CNTN5 (rs1813443) G/C	chr11:100140279		Association<br>One study with 126 patients found an association with response. OR(95% CI): 6.13 (1.74-21.63) P=0.005[15]<br><br>No association<br>No studies				
CRP (rs1130864) G/A	chr1:159713301					Association<br>One study with 79 patient found an association with response. P=4.09x10^{-05}.[8]<br><br>No association<br>No studies	
DAXX (rs2239839) C/A	chr6:33320298					Association<br>One study with 459 patients found an association with response. OR (95% CI): 2.08 (1.45–2.99). P=7.37E-05.[1]<br><br>No association<br>No studies	

DENND1B (rs2488397) G/C	chr1:197732149					Association<br>One study with 459 patients found an association with response.<br>OR (95% CI) = 1.85 (1.26–2.72)<br>P= 0.002.[1]<br><br>No association<br>No studies	
DNMT3B (rs6087990) T/C	chr20:32762102					Association<br>One study with 359 patients found an association with response.<br>OR=1.637<br>P=0.013.[4]<br><br>No association<br>No studies	
DTNBP1 (rs10456777) G/C	chr6:15857018					Association<br>One study with 459 patients found an association with response.<br>OR (95% CI): 3.63 (2.09–6.32)<br>P= 5.15E-06[1]<br><br>No association<br>No studies	
FAS (rs7896789) T/C	chr10:89005107					Association<br>One study with 107 patients found an	

						association with response.<br>OR (95% CI): 3.63 (1.261–10.425). P= 0.03.[16]<br><br>No association<br>No studies	
FAS Ligand (rs763110) C/T	chr1:172658358		Association<br>One study with 287 patients found an association with response.<br>OR (95% CI): 0.77 (0.66-0.90).<br>P=0.001.[11]<br><br>No association<br>One study with 82 patients found no association with response.[12]			Association<br>One study with 121 patients found an association with response.<br>P=0.042.[7]<br><br>No association<br>No studies	
FCGR3A (rs12128686) T/C	chr1:161548967					Association<br>One study with 107 patients found an association with response.<br>OR (95% CI): 3.63 (1.261–10.425) P=0.01[16]<br><br>No association<br>No studies	
FCGR3A (rs373184583) G/A	chr1:161548886					Association<br>One study with	

						107 patients found an association with response.<br>OR (95% CI): 3.63 (1.261–10.425) P=0.01.[16]<br><br>No association<br>No studies	
FCGR3A (rs111504845) G/A	chr1:161595689		Association<br>One study with 206 patients found an association with response. OR (95% CI): 2.50 (1.00–6.33). P=0.047[10]<br><br>No association<br>No studies				
FCGR3A (rs396991) A/C	chr1:161544752		Association<br>One study with 76 patients found an association with response. OR (95% CI): 8.89 (2.52–31.41). P=0.001.[17]<br><br>No association<br>Four studies found no association with response.		Association<br>No studies<br><br>No association<br>One study with 121 patients found no association with response. P=NS[7]		

			Louis et al:<br>n= 200 patients found no association with response.: RR (95% CI): 1.19 (0.99–1.43) P=0.15[18]<br><br>Matsuoka et al: n=121 OR (95% CI): 0.92 (0.45-1.90). P=0.82[19]<br><br>Papamichaela et al:<br>n= 106<br>OR (95% CI): 0.97 (0.37-2.51). P=1.[20]<br><br>Louis et al:<br>n=344<br>P=0.195[21]				
FCGR3A (rs6672453) C/A	chr1:161546221					Association<br>One study with 107 patients found an association with response.<br>OR (95% CI): 3.63 (1.26–10.43) P=0.01.[16]<br><br>No association<br>No studies	

FCGR3A (rs7539036) G/A	chr1:161542941					Association<br>One study with 107 patients found an association with response.<br>OR (95% CI): 3.63 (1.26–10.43) P=0.01.[16]<br><br>No association<br>No studies	
FIBP (rs568617) C/T	chr11:65885771					Association<br>One study with 231 patients found an association with primary non-response.<br>OR: 0.39. P=0.042.[2]<br><br>No association<br>No studies	
GJB3 (rs145751680) G/A	chr1:34785012		Association<br>One study with 135 patients found an association with response. OR (95% CI): NA. P=2.24E-06[22]<br><br>No association<br>No studies				
GLIS3 (rs1330307) A/C	chr9:4305064					Association<br>One study with 231 patients found an	

						association with response.<br>OR: 0.23. P=5.65E-06[2]<br><br>No association<br>No studies	
GNA12 (rs1182188) T/C	chr7:2830351					Association<br>One study with 359 patients found an association with response.<br>OR=1.859.<br>P=0.01439[4]<br><br>No association<br>No studies	
GSN (rs55689715) T/C	chr9:121213595					Association<br>One study with 549 patients found an association with response.<br>OR= 3.324.<br>P=6.00E-05.[4]<br><br>No association<br>No studies	
HFE (rs2071303) T/C	chr6:26091108	Association<br>One study with 68 patients found an association with response.<br>OR (95% CI): 4.83 (1.52–15.37).<br>P=0.009.[23]<br><br>No association<br>No studies					

IBD5 (No rs number)	Chr 5q31		Association<br>One study with 40 patients found an association with response. OR (95%CI) = 3.42 (1.09-10.68). *P*<0.05.[24]<br><br>No association<br>No studies				
ICOSLG (rs762421) G/A	chr21:44195678		Association<br>One study with 94 patients found an association with response. RR=2.6 (1.0, 7). P=0.045.[6]<br><br>No association<br>No studies				
IFIH1 (rs1990760) C/T	chr2:162267541					Association<br>One study with 459 patients found an association with time to loss of response.<br>HR (95% CI) = 0.79 (0.65–0.95). P=0.01.[1]<br><br>No association<br>No studies	
IFNG (rs2430561) T/A	chr12:68158742					Association<br>One study with 738 patients	

						found an association with response.<br>OR (95% CI): 1.66 (1.05-2.62) P=0.03.[14]<br><br>No association<br>No studies	
IFNGR1 (rs2234711) A/G	chr6:137219383					Association<br>One study with 253 patients found an association with response.<br>OR (95% CI): 0.29 (0.11–0.78). P=0.01.[25]<br><br>No association<br>No studies	
IFNGR1 (rs8126756) T/C	chr21:33403138					Association<br>One study with 474 patients found an association with response.<br>OR (95% CI): 0.09 (0.01-0.65). P=0.02.[25]<br><br>No association<br>No studies	
IFNGR2 (rs2284553) A/G	chr21:33404389					Association<br>One study with 231 patients found an association with response.	

						OR: 1.80.<br>P=0.037.[2]<br><br>No association<br>No studies	
IL10 (rs1800872) T/G	chr1:206773062					Association<br>One study with 209 patients found an association response<br>HR (95% CI): 4.75 (1.16–19.52).<br>P=0.031[26]<br><br>No association<br>One study with 255 patients found no association with response.<br>OR (95% CI): 0.57(0.25-1.26).<br>P=0.16[14]	
IL12B (rs3212217) C/G	chr5:159328122					Association<br>One study with 251 patients found an association with response.<br>OR (95% CI): 0.33 (0.15–0.69).<br>P=0.0036.[25]<br><br>No association<br>No studies	
IL13 (rs1295686) T/C	chr5:132660151	Association<br>One study with 97 patients found an					

		association with response. P=6.07E-03.[9]<br><br>No association<br>No studies					
IL17A (rs2275913) G/A	chr6:52186235	Association<br>One study with 102 patients found an association with response. P=0.006.[27]<br><br>No association<br>No studies	Association<br>No studies<br><br>No association<br>One study with 103 patients found no association with response. OR (95% CI): 0.81 (0.36–1.83), P=0.61.[28]			Association<br>Two studies found an association with response.<br><br>Salvador-Martın et al:<br>n=209<br>HR (95% CI): 0.32 (0.111–0.920)<br>P=0.034.[26]<br><br>Bank et al:<br>n=738<br>OR (95% CI): 0.47(0.21-1.01). P=0.05.[14]<br><br>No association<br>No studies	
IL17F (rs766748) G/A	chr6:52238101		Association<br>One study with 103 patients found an association with response. OR (95% CI): 0.203 (0.053–0.779), P=0.019.[28]<br><br>No association<br>No studies				

IL17F (rs9382084) G/T	chr6:52240869		Association<br>One study with 103 patients found an association with response. OR (95% CI): 9.671 (0.554–168.8), P=0.037.[28]<br><br>No association<br>No studies				
IL18 (rs187238) C/G	chr11:112164265					Association<br>One study with 1069 patients found an association with response.<br>OR (95% CI): 1.35 (1.00 - 1.82). P=0.047.[29]<br><br>No association<br>One study with 475 patients found no association with response.<br>OR (95% CI): 1.43 (0.73-2.81). P=0.30[25]	
IL18 (rs1946518) T/G	chr11:112164735					Association<br>Two studies found an association with response.<br><br>Bank et al: n=1783	

						OR (95%): 1.24 (1.01-1.53). P=0.04.[29]<br><br>Bank et al: n=252 OR (95% CI): 4.52 (1.43–14.30). P=0.01[25]<br><br>No association<br>No studies	
IL18RAP (rs6708413) G/A	chr2:102446909					Association<br>One study with 359 patients found an association with primary non-response. OR=1.712 P=0.045.[4]<br><br>No association<br>No studies	
IL1B (rs1071676) C/G	chr2:112829856					Association<br>One study with 107 patients found an association with response. OR (95% CI): 2.59 (1.09–6.11) P=0.03[16]<br><br>No association<br>No studies	
IL1B (rs1143634) G/A	chr2:112832813		Association<br>One study with 29 patient found an			Association<br>One study with 107 patients found an	

			association with lower response. P=0.027[30]<br><br>No association<br>No studies			association with non-response. OR (95% CI): 2.59 (1.093–6.113) P=0.03[16]<br><br>No association<br>No studies	
IL1B (rs1143637) C/T	chr2:112831756					Association<br>One study with 107 patients found an association with response.<br>OR (95% CI): 2.59 (1.09–6.11).<br>P=0.03.[16]<br><br>No association<br>No studies	
IL1B (rs1143639) C/T	chr2:112831216					Association<br>One study with 107 patients found an association with response.<br>OR (95% CI): 2.59 (1.09–6.11).<br>P=0.03.[16]<br><br>No association<br>No studies	
IL1B (rs4848306) G/A	chr2:112840530					Association<br>One study with 738 patients found an association with response.<br>OR (95% CI): 1.85 (1.05-3.27).	

						P=0.03.[14]<br><br>No association<br>No studies	
IL1R (rs2041747) G/A	chr2:102171949					Association<br>One study with 107 patients found an association with response.<br>OR (95% CI): 5.29 (1.178–23.708).<br>P= 0.02.[16]<br><br>No association<br>No studies	
IL1RN (rs4251961) T/C	chr2:113116890					Association<br>Two studies found an association with response.<br><br>Bank et al: n=256. OR (95% CI): 0.28(0.09-0.89). P=0.03.[14]<br><br>Bank et al: n=1783<br>OR (95% CI): 0.81 (0.66-1.00).<br>P=0.049.[29]<br><br>No association<br>No studies	
IL1RN (rs396201) T/C	chr2:113134198		Association<br>One study with 206 patients found an association with response.				

			OR (95% CI): 2.18 (1.05–4.56). P=0.035[10]<br><br>No association<br>No studies				
IL17RA (rs2241046) C/T	chr22:17105581		Association<br>One study with 206 patients found an association with response. OR (95% CI): 0.17 (0.04–0.80). P=0.012[10]<br><br>No association<br>No studies				
IL2RA (rs12722515) C/A	chr10:6039267					Association<br>One study with 359 patients found an association with durable response. OR= 2.997. P=0.0020.[4]<br><br>No association<br>No studies	
IL6 (rs10499563) T/C	chr7:22720869					Association<br>Two studies found an association with response.<br><br>Bank et al: n=732<br>OR 95% CI: 2.26	

						(1.18-4.32).<br>P=0.01[14]<br><br>Salvador-Martın et al: n=209<br>HR (95% CI): 0.210 (0.047–0.947)<br>P=0.042.[26]<br><br>No association<br>One study with 1782 patients found no association with response.<br>OR (95% CI): 1.31 (0.99-1.71).<br>P=0.052[29]	
IL6 -174 (rs1800795) G/C	chr7:22727026		Association<br>One study with 64 patients found an association with response.<br>P<0.05.[31]<br><br>No association<br>No studies				
IPMK (rs2790216) G/A	chr10:58238165					Association<br>One study with 231 patients found an association with durable response.<br>OR: 1.56.<br>P=0.048.[2]<br><br>No association	

						No studies	
IRAK4. rs4251580. C/T	chr12:43780963		Association<br>One study with 127 patients found an association with response. OR=0.289. P = 0.014.[32]<br><br>No association<br>No studies				
IRF1-AS1 (rs2188962) C/T	chr5:132435113		Association<br>One study with 94 patients found an association response. RR=2.3 (2.2-4.7). P=0.028.[6]<br><br>No association<br>No studies				
IRGM (rs13361189) T/C	chr5:150843825		Association<br>One study with 570 patients found an association with response OR (95% CI): 2.4 (1.0–5.7). P= 0.048[33]<br><br>No association<br>One study with 259 patients found no association				

			with response.[34]				
JAK2 (rs12343867) T/C	chr9:5074189					Association<br>Two studies found an association with response.<br><br>Bank et al:<br>n=255.<br>OR (95% CI):<br>0.17 (0.03–0.85).<br>P=0.03[25]<br><br>Bank et al:<br>n=1783<br>OR (95% CI):<br>1.24 (1.01-1.53)<br>P=0.04[29]<br><br>No association<br>No studies	
Keratin 4 (rs7956809) C/G	chr12:52808399					Association<br>One study with 359 patients found an association with response.<br>OR=3.204<br>P=4.30E-05.[4]<br><br>No association<br>No studies	
KLHL1 (rs9572250) A/G	chr13:69728046					Association<br>One study with 474 patients found an association with response.<br>OR (95% CI) =3.00 (1.84–4.89).	

						P = 3.19E-06.[35]<br><br>No association<br>No studies	
LINC00484/ LOC100507103 (Potential genes: NFIL3) (rs4743820) C/T	chr9:91166134					Association<br>One study with 231 patients found an association with response.<br>OR: 1.81.<br>P=0.044.[2]<br><br>No association<br>No studies	
LOC101928354 (rs17119) G/A	chr6:14719265					Association<br>One study with 359 patients found an association with durable response.<br>OR=1.7. P=0.047[4]<br><br>No association<br>No studies	
LOC105371082 (potential genes: SOCS1, LITAF, RMI2) (rs529866) C/T	chr16:11279463					Association<br>One study with 231 patients found an association with response.<br>OR: 2.18.<br>P=0.001.[2]<br><br>No association<br>No studies	
LOC105371082 (rs12051532) C/T	chr16:11285459					Association<br>One study with 231 patients found an	

						association with response.<br>OR: 2.35.<br>P=8.44E-06[2]<br><br>No association<br>No studies	
LOC105372112 (rs8083571) A/G	chr18:49691751					Association<br>One study with 359 patients found an association with response. OR= 2.948. P=2.40E-05.[4]<br><br>No association<br>No studies	
LOC105372912 (rs144256942) A/G	chr1:213558739					Association<br>One study with 474 patients found an association with response.<br>OR (95% CI): 9.88 (2.88–33.9).<br>P=1.14E-05.[35]<br><br>No association<br>No studies	
LOC107985484 (rs2836866) T/C	chr21:39067336					Association<br>One study with 459 patients found an association with time to loss of response.<br>HR (95% CI) = 1.54 (1.24–1.91).	

						P= 7.86E-05.[1]<br><br>No association<br>No studies	
LOC107986022 (rs2045307) C/T	chr3:117919374					Association<br>One study with 359 patients found an association with response.<br>OR= 2.785<br>P=7.40E-05.[4]<br><br>No association<br>No studies	
LOC107986770 (rs1568885) T/A	chr7:13597906		Association<br>One study with 126 patients found an association with response.<br>OR (95% CI): 8.14(1.35-49.05),<br>P=0.024[15]<br><br>No association<br>No studies				
LSP1 (rs907611) G/A	chr11:1852842					Association<br>One study with 231 patients found an association with response<br>OR: 1.54.<br>P=0.039.[2]<br><br>No association<br>No studies	

LTF (rs762787) C/T	chr3:46447445					Association<br>One study with 474 patients found an association with response.<br>OR (95% CI) = 4.27 (2.16–8.48).<br>P= 6.47E-06.[35]<br><br>No association<br>No studies	
LUZP2 (rs1915063) A/G	chr11:24078227					Association<br>One study with 459 patients found an association with time to loss of response<br>HR (95% CI) = 1.54 (1.27–1.87).<br>P=1.10E-05.[1]<br><br>No association<br>No studies	
LY96 (rs11465996) C/G	chr8:73989727					Association<br>Two studies found an association with response.<br><br>Salvador-Martın et al: n=209<br>HR (95% CI): 6.05 (1.22–30.13).<br>P=0.028.[26]<br><br>Bank et al:<br>n=255.<br>OR(95% CI): 0.32 (0.14-0.75).	

						P=0.01.[14]<br><br>No association<br>No studies	
MAP3K14 (rs7222094) T/C	chr17:45290287					Association<br>One study with 738 patients found an association with response.<br>OR (95% CI): 1.92 (1.00-3.68). P=0.05.[14]<br><br>No association<br>No studies	
MIF (rs755622) G/C	chr22:23894205	Association<br>One study with 102 patients found an association with response. P=0.004.[27]<br><br>No association<br>No studies					
MMP25 / LOC105376265 (rs117642371) G/T	chr9:123311401		Association<br>One study with 135 patients found an association with response. OR (95% CI): 30.68 (3.49–270). P=2.79E-06[22]<br><br>No association<br>No studies				

MSGN1 (rs34069439) A/T	chr2:17816758		Association<br>One study with 135 patients found an association with response. OR (95% CI): 18.83 (3.66–96.94). P=1.73E-06.[22]<br><br>No association<br>No studies				
MST1/APEH (rs3197999) G/A	chr3:49684099					Association<br>One study with 359 patients found an association with response. OR=0.51 P=0.025.[4]<br><br>No association<br>No studies	
NFKB1 (rs3774934) A/G	chr4:102506319	Association<br>One study with 79 patients found an association with response. P=0.014.[8]<br><br>No association<br>No studies					
NF-kB1 (rs7674004) G/A	chr4:102619156		Association<br>One study with 206 patients found an association with response.				

			OR (95% CI): 0.47 (0.23–0.97). P=0.039[10]<br><br>No association<br>No studies				
NFKBIA (rs696) C/T	chr14:35401887					Association<br>One study with 1783 patients found an association with response.<br>OR (95% CI): 1.25 (1.01 - 1.54). P=0.04.[29]<br><br>No association<br>One study with 738 patients found no association with response. OR (95% CI): 1.01 (0.57-1.78). P=0.97.[14]	
NLRP3 (rs10754558) G/C	chr1:247448734					Association<br>One study with 738 patient found an association with response.<br>OR (95% CI): 1.60 (1.02–2.52). P=0.04[25]<br><br>No association<br>No studies	
NLRP3 (rs4612666) C/T	chr1:247435768					Association<br>One study with 1783 patients found an	

						association with response.<br>OR (95% CI): 0.73 (0.57 - 0.95).<br>P=0.02.[29]<br><br>No association<br>One study with 738 patients found no association with response.<br>OR (95% CI): 0.70 (0.47-1.04).<br>P=0.08.[14]	
No name (rs2651244) G/A	chr1:70529879					Association<br>One study with 359 patients found an association with response.<br>OR= 1.51.<br>P=0.041.[4]<br><br>No association<br>No studies	
No name (Potential gene: RBP-J) (rs4692386) T/C	chr4:26130739					Association<br>One study with 231 patients found an association with response.<br>OR: 0.57.<br>P=0.004.[2]<br><br>No association<br>No studies	
No name (Potential genes: CCL2, CCL7) (rs3091315) A/G	chr17:34266646					Association<br>One study with	

						231 patients found an association with response.<br>OR: 0.63.<br>P=0.024.[2]<br><br>No association<br>No studies	
No name (Potential genes: NRP1; PARD3) (rs6481864) T/C	chr10:33639098					Association<br>One study with 459 patients found an association with time to loss of response.<br>HR (95% CI) = 2.31 (1.54–3.48).<br>P= 5.19E-05.[1]<br><br>No association<br>No studies	
No name (Potential genes: PRDM1; ATG5) (rs62421049) T/A	chr6:105965469					Association<br>One study with 459 patients found an association with time to loss of response.<br>HR (95% CI) = 1.83 (1.39–2.40).<br>P=1.68E-05.[1]<br><br>No association<br>No studies	
No name (rs10512734) A/G	chr5:40393503	Association<br>One study with 97 patients found an association with response.					

		OR (95% CI): 0.635 (0.541–0.746). P=2.76E-02[9]<br><br>No association<br>No studies					
No name (rs10761659) A/G	chr10:62685804					Association<br>One study with 359 patients found an association with response. OR= 1.659. P=0.041[4]<br><br>No association<br>No studies	
No name (rs11229555) G/T	chr11:5864121					Association<br>One study with 359 patients found an association with response OR= 0.58. P=0.01[4]<br><br>No association<br>No studies	
No name (rs9319943) T/C	chr18:59212595					Association<br>One study with 231 patients found an association with durable response. OR: 1.66. P=0.037.[2]<br><br>No association<br>No studies	

No name (rs9904253) A/G	chr17:70934698					Association<br>One study with 359 patients found an association with response. OR= 0.47 P=9.80E-05.[4]<br><br>No association<br>No studies	
No name Suggestions: FAP; IFIH1 (rs2111485) A/G	chr2:162254026					Association<br>One study with 459 patients found an association with time to loss of response. HR (95% CI) 0.74 (0.61–0.89) P=0.002.[1]<br><br>No association<br>No studies	
NOD2 (rs5743289) C/T	chr16:50722863					Association<br>One study with 231 patients found an association with response. OR: 1.79. P=0.033.[2]<br><br>No association<br>No studies	
OSMR (rs357291) C/A	chr5:38923677		Association<br>One study with 206 patients found an association				

			primary non-response.<br>OR (95% CI): 0.33 (0.15–0.73).<br>P=0.005[10]<br><br>No association<br>No studies				
PHTF1 (rs6679677) C/A	chr1:113761186					Association<br>One study with 231 patients found an association with response.<br>OR: 2.26.<br>P=0.041.[2]<br><br>No association<br>No studies	
PITX1-AS1 (rs254560) G/A	chr5:135107916					Association<br>One study with 359 patients found an association with response.<br>OR= 0.65.<br>P=0.027.[4]<br><br>No association<br>No studies	
PLCL1 (rs1440088) T/G	chr2:198006693					Association<br>One study with 359 patients found an association with response.<br>OR=1.74.<br>P=0.035.[4]	

						No association<br>No studies	
PLIN2 (rs2228416) C/T	chr9:19126283					Association<br>One study with 474 patients found an association with response.<br>OR (95% CI): 5.33 (2.43–11.7).<br>P= 3.76E-06.[35]<br><br>No association<br>No studies	
PTPN2 (rs1893217) A/G	chr18:12809341				Association<br>One study with 1843 patients found an association with response.<br>P=0.05.[36]<br><br>No association<br>No studies		
PTPN2 (rs7234029) A/G	chr18:12877061	Association<br>No studies<br><br>No association<br>One study with 229 patients found no association with response.<br>P=0.919.[5]	Association<br>No studies<br><br>No association<br>One study with 125 patients found no association with response.<br>P=0.808.[5]				Association<br>One study with 110 patients found an association with response.<br>P=0.005.[5]<br><br>No association<br>No studies
RAB38 (rs9144)	chr11:88113589		Association<br>One study with 135 patients found an association with loss of				

			response.<br>OR (95% CI): 3.81 (2.10–6.89). P=4.60E-06.[22]<br><br>No association<br>No studies				
RalGDS/AF-6 (rs2682714) T/C	chr12:64624412					Association<br>One study with 359 patients found an association with response.<br>OR= 2.4.<br>P=6.10E-05.[4]<br><br>No association<br>No studies	
RHCG (rs2289352) C/T	chr15:89479606					Association<br>One study with 459 patients found an association with response.<br>OR (95% CI): 2.45 (1.60–3.74).<br>P=3.46E-05.[1]<br><br>No association<br>No studies	
RIPK1 (rs9378763) C/A	chr6:3071375		Association<br>One study with 206 patients found an association with response.<br>OR (95% CI):				

			2.11 (1.00–4.48). P=0.047[10]<br><br>No association<br>No studies				
RIT1 (rs670523) A/G	chr1:155908941					Association<br>One study with 231 patients found an association with response.<br>OR: 0.64. P= 0.021.[2]<br><br>No association<br>No studies	
RORC (rs4845604) G/A	chr1:151829204					Association<br>One study with 359 patients found an association with response.<br>OR=2.46 P=0.00189.[4]<br><br>No association<br>No studies	
RSPO3 (rs2503322) A/G	chr6:127136115					Association<br>One study with 359 patients found an association with response.<br>OR= 0.59 P=0.041.[4]<br><br>No association<br>No studies	

SFMBT1 (rs9847710) T/C	chr3:53028645					Association<br>One study with 359 patients found an association with response.<br>OR= 0.51.<br>P=0.013.[4]<br><br>No association<br>No studies	
SH2B1 (rs7201929) T/C	chr16:28860645					Association<br>One study with 359 patients found an association with response.<br>OR= 0.4505.<br>P=8.10E-05.[4]<br><br>No association<br>No studies	
SLIT1 (rs7093856) G/A	chr10:97134109					Association<br>One study with 459 patients found an association with time to loss of response.<br>HR (95% CI) = 2.14 (1.48–3.09).<br>P= 5.30E-05.[1]<br><br>No association<br>No studies	
SMAD3 (rs17293632) C/T	chr15:67150258					Association<br>One study with 459 patients found an association with	

						time to loss of response<br>HR (95% CI) 0.69 (0.55–0.86). P=0.0008[1]<br><br>No association<br>No studies	
SMURF1 (rs9297145) C/A	chr7:99161494					Association<br>One study with 359 patients found an association with response.<br>OR= 1.57.<br>P=0.046.[4]<br><br>No association<br>No studies	
SP140 (rs6716753) T/C	chr2:230232414					Association<br>One study with 231 patients found an association with response.<br>OR: 0.61.<br>P=0.026.[2]<br><br>No association<br>No studies	
SYNGAP1 (rs10807124) G/A	chr6:33436287					Association<br>One study with 459 patients found an association with response.<br>OR (95% CI): 2.13 (1.47–3.07).<br>P= 5.62E-05[1]	

						No association<br>No studies	
TAGAP (rs212388) C/T	chr6:159069404					Association<br>One study with 359 patients found an association with response.<br>OR=0.62<br>P=0.0137.[4]<br>No association<br>No studies	
TBC1D5 (rs17200795) A/G	chr3:17361100					Association<br>One study with 359 patients found an association with response.<br>OR=3.193<br>P=4.60E-05[4]<br><br>No association<br>No studies	
TBX21 (rs17250932) T/C	chr17:47731941					Association<br>One study with 256 patients found an association with response.<br>OR (95% CI): 0.06 (0.01–0.80).<br>P=0.03[25]<br><br>No association<br>No studies	
TICAM1. rs7255265. T/C	chr19:4818366		Association<br>One study with 127 patients found an association				

			with response<br>OR=0.252.<br>P=0.038.[32]<br><br>No association<br>No studies				
TLR2 (rs11938228) C/A	chr4:153700794					Association<br>Two studies found an association with response.<br><br>Bank et al:<br>n=738. OR (95% CI): 0.66 (0.48-0.92) P=0.01[14]<br><br>Bank et al:<br>n=714<br>OR (95% CI):<br>0.55 (0.33 - 0.92)<br>P=0.02[29]<br><br>No association<br>No studies	
TLR2 (rs1816702) T/C	chr4:153688371		Association<br>One study with 132 patients found an association with response<br>P=0.014.[37]<br><br>No association<br>No studies			Association<br>One study with 466 patients found an association with response<br>OR (95% CI): 2.02 (1.04-3.95).<br>P=0.04.[14]<br><br>No association<br>No studies	
TLR2 (rs3804099) T/C	chr4:153703504		Association<br>One study with 132 patients			Association<br>One study with 738 patients	

			found an association with response P=0.042.[37]<br><br>No association<br>No studies			found an association with response.<br>OR (95% CI): 2.25 (1.26-4.01).<br>P=0.01.[14]<br><br>No association<br>No studies	
TLR2 (rs4696480) T/A	chr4:153685974					Association<br>One study with 253 patients found an association with response.<br>OR (95% CI): 0.29 (0.12-0.70).<br>P=0.01.[14]<br><br>No association<br>No studies	
TLR4 (rs1554973) T/C	chr9:117718534					Association<br>Two studies found an association with response.<br><br>Bank et al:<br>n=738<br>OR (95% CI):<br>0.72 (0.52-0.99)<br>P=0.04[14]<br><br>Bank et al:<br>n=1783<br>OR (95% CI):<br>0.80 (0.65 - 0.98)<br>P=0.03[29]<br><br>No association<br>No studies	

TLR4 (rs5030728) G/A	chr9:117712004					Association<br>Two studies found an association with response.<br><br>Bank et al:<br>n=1783<br>1.46 (1.01-2.11).<br>P=0.04.[29]<br><br>Bank et al:<br>n=738<br>OR (95%): 1.45 (1.06-2.00)<br>P=0.02.[14]<br><br>No association<br>No studies	
TLR5 (rs5744174) T/C	chr1:223111186					Association<br>One study with 479 patients found an association with response.<br>OR (95% CI): 0.36 (0.16–0.81).<br>P=0.01.[25]<br><br>No association<br>No studies	
TLR9 (rs187084) A/G	chr3:52227015					Association<br>One study with 738 patients found an association with response<br>OR (95% CI): 1.99 (1.04-3.82).<br>P=0.04.[14]	

						No association<br>No studies	
TLR9 (rs352139) T/C	chr3:52224356					Association<br>One study with 738 patients found an association with response.<br>OR (95% CI): 0.48 (0.24-0.96) P=0.04.[14]<br><br>No association<br>No studies	
TNF-α -857 (rs1799724) C/T	chr6:31574705		Association<br>One study with 121 patients found an association with response. OR (95% CI): 0.33 (0.12-0.95). P=0.04.[19]<br><br>No association<br>Three studies found no association with response.<br><br>Dideberg et al: n=222 P=0.672[38]<br><br>Duricova et al n=82. P=NS[12]<br><br>Papamichael et al: n=106				

			OR (95% CI): 1.27 (0.51-3.20). P=0.65.[20]				
TNF-α-238 (rs361525) G/A	chr6:31575324		Association<br>No studies<br><br>No association<br>Three studies found no association with response.<br><br>Dideberg et al: n=214. P=0.969.[38]<br><br>Matsuoka et al: n=121. OR (95% CI): 1.82 (0.34-9.77). P=0.49.[19]<br><br>Papamichael et al: n=106 OR (95% CI): 1.01 (0.39-2.58) P=1. [20]			Association<br>One study with 738 patients found an association with response. OR(95% CI): 0.43(0.19-0.97) P=0.04.[14]<br><br>No association<br>Two studies found no association with response.<br><br>Netz et al: n=121. P=NS.[7]<br><br>Lopez-Hernandez et al: n=82. P=NS.[39]	
TNF-α-308 (rs1800629) G/A	chr6:31575254		Association<br>No studies<br><br>No association<br>Seven studies found no association with response.<br><br>Dideberg et al: n=222. P=0.865[38]			Association<br>One study with 75 patients found an association with non-response. P=0.017[39]<br><br>No association<br>Two studies found no association with response.	

			Louis et al: n=220. P=NS[40]<br><br>Matsuoka et al: n=121. OR (95% CI): 0.94 (0.20-4.38). P=0.93[19]<br><br>Duricova et al: n=82. P=NS. [12]<br><br>Papamichael et al: n=106 OR (95% CI): 0.61 (0.24-1.56). P=0.33. [20]<br><br>Curci et al: n=76. OR (95% CI): 1.13 (0.34-3.81). P=0.84.[17]<br><br>Naviglio et al n=64.[31]			Netz et al: n=121 P= 0.088[7]<br><br>Bank et al: n=738 OR (95% CI): 1.26 (0.81-1.95) P=0.30[14]	
TNFAIP3 (rs6927172) C/G	chr6:137681038					Association<br>One study with 738 patients found an association with response. OR (95% CI): 0.62(0.42-0.92). P=0.02.[14]<br><br>No association<br>No studies	

TNFAIP6 / LOC101929319 (rs11677200) T/C	chr2:151354129		Association<br>One study with 332 patients found an association with response. OR (95% CI) = 0.5 (0.27–0.93). P=0.017.[41]<br><br>No association<br>No studies				
TNFRSF1A (rs1800693) T/C	chr12:6330843	Association<br>One study with 102 patients found an association with response. [27]<br><br>No association<br>No studies	Association<br>One study with 616 patients found an association with response. OR(95% CI): 2.34 (1.26-4.37). P=6.09x10-3[42]<br><br>No association<br>No studies				
TNFRSF1A (rs4149570) A/C	chr12:6342424		Association<br>No studies<br><br>No association<br>One study with 80 patients found no association with response. P=0.62.[43]			Association<br>Two studies found an association with beneficial response.<br><br>Bank et al: n=738. OR (95% CI): 1.94 (1.05-3.60) P=0.04.[14]<br><br>Bank et al: n=1069 OR (95% CI): 1.92	

						(1.02 - 3.60). P=0.04.[29]<br><br>No association<br>No studies	
TNFRSF1A (rs767455) A/G	chr12:6341779		Association<br>One study with 80 patients found an association with response. P=0.04.[43]<br><br>No association<br>Two studies found no association with response.<br><br>Matsuoka et al: n=121. OR (95% CI): 0.87 (0.36-2.12). P=0.76.[19]<br><br>Medrano et al: n=297 OR (95% CI): 0.84 (0.34–2.04). P=0.67.[44]				
TNFRSF1B (rs1061622) T/G	chr1:12192898		Association<br>Three studies found an association with response response.[45]<br><br>Mascheretti et al:				

			n=90.<br>P=0.036[45]<br><br>Medrano et al:<br>n=131.<br>OR (95% CI):<br>2.86 (1.04–<br>8.33).<br>P=0.023[44]<br><br>Steenholdt et al:<br>n=124.<br>OR (95% CI):<br>5.5 (1.5 – 25.5).<br>P=0.007.[46]<br><br>No association<br>Two studies found no association with response:<br><br>Matsuoka et al:<br>n=121<br>OR (95% CI):<br>1.00 (0.43-<br>2.33). P=0.99.[19]<br><br>Matsukura et al:<br>n=80. P=0.29.[43]				
TNFRSF1B. (rs1061624). A/G	chr1:12207208		Association<br>One study with 132 patients found an association with response P=0.024.[37]				

			No association<br>No studies				
TNFRSF1B (rs3397) C/T	chr1:12207235		Association<br>No studies<br><br>No association<br>Two studies found no association with response:<br><br>Matsukura et al:<br>n=80. P=0.55.[43]<br><br>Medrano et al:<br>n=297.<br>OR (95% CI):<br>1.45 (0.42–6.37).<br>P= 0.53.[44]			Association<br>One study with 209 patients found an association with response.<br>P=0.043.[26]<br><br>No association<br>No studies	
TNFRSF1B (rs5746053) G/A	chr1:12202241					Association<br>One study with 107 patients found an association with response.<br>OR (95% CI): 0.09 (0.005–1.560)<br>P=0.02.[16]<br><br>No association<br>No studies	
TNFRSF1B (rs976881) T/C	chr1:12173697		Association<br>One study with 124 patients				

			found an association with loss of response. OR (95%): 3.3 (1.2 – 9.1), P = 0.014.[46]<br><br>No association<br>One study with 121 patients found no association with response. OR (95% CI): 1.04 (0.46-2.37). P=0.93.[19]				
TNFRSF9 / PARK7 (rs3766606) G/T	chr1:7962137					Association<br>One study with 359 patients found an association with primary non-response. OR=0.32 P=0.022.[4]<br><br>No association<br>No studies	
TNFSF15 (rs4246905) T/C	chr9:114790969					Association<br>One study with 359 patients found an association with primary non-response. OR=1.66. P=0.047.[4]<br><br>No association	

						No studies	
TNFSF4 (rs116724455) T/C	chr1:173221946					Association<br>One study with 474 patients found an association with response.<br>OR (95% CI): 19.9 (4.57–86.7). P= 4.79E-08.[35]<br><br>No association<br>No studies	
TRAF3IP2-ASI (rs3851228) A/T	chr6:111526988					Association<br>One study with 231 patients found an association with response.<br>OR: 2.33.<br>P=0.027.[2]<br><br>No association<br>No studies	
TRIB1 (rs921720) A/G	chr8:12552242					Association<br>Two studies found an association with primary non-response and durable response, respectively.<br><br>Barber et al:<br>n=359<br>OR= 0.586.<br>P=0.04991.[4]<br><br>Burke et al:<br>n=231<br>OR: 0.67.	

						P=0.042.[2]<br><br>No association<br>No studies	
TRIM21 (rs2269330) A/G	chr11:4385253		Association<br>One study with 206 patients found an association with response. OR (95% CI): 0.35 (0.16-0.75). P=0.006[10]<br><br>No association<br>No studies				
UBAC2, GPR18 (rs3742130) G/A	chr13:99255087					Association<br>One study with 231 patients found an association with response. OR: 1.98. P=0.023.[2]<br><br>No association<br>No studies	
XBBP1 (rs35873774) A/G	chr22:28795944		Association<br>One study with 570 patients found an association with response. OR (95% CI): 3.7 (1.2–10.8). P=0.016.[33]<br><br>No association<br>No studies				

ZFP36L1 (rs194749) T/C	chr14:68807188					Association<br>One study with 359 patients found an association with response<br>OR= 1.663. P=0.049.[4]<br><br>No association<br>No studies	
ZFP90 (rs1728785) A/C	chr16:68557327					Association<br>One study with 359 patients found an association with response.<br>OR=0.5 P=0.045[4]<br><br>No association<br>No studies	
ZNF133 (rs2228273) G/A	chr20:18315432		Association<br>One study with 135 patients found an association with response.<br>OR (95% CI): 6.11 (2.59–14.46).<br>P=4.70E-06.[22]<br><br>No association<br>No studies				
ZNF133 (rs34099160) C/T	chr20:18315086		Association<br>One study with 135 patients found an association with response.				

			OR (95% CI): 6.11 (2.659–14.46). P=4.70E-06.[22]<br><br>No association<br>No studies				
ZNF227 (rs2168989) G/T	chr19:44208531					Association<br>One study with 459 patients found an association with time to loss of response. HR (95% CI): 0.65 (0.53–0.80). P= 4.98E-05.[1]<br><br>No association<br>No studies	

References

1. Yoon SM, Haritunians T, Chhina S, et al. Colonic Phenotypes Are Associated with Poorer Response to Anti-TNF Therapies in Patients with IBD. *Inflamm Bowel Dis*. 2017;23(8):1382-1393. doi:10.1097/MIB.0000000000001150
2. Burke KE, Khalili H, Garber JJ, et al. Genetic Markers Predict Primary Nonresponse and Durable Response to Anti-Tumor Necrosis Factor Therapy in Ulcerative Colitis. *Inflamm Bowel Dis*. 2018;24(9):1840-1848. doi:10.1093/ibd/izy083
3. Repnik K.; Jurgec S.; Koder S.; Ferkolj I.; Potocnik U.; Missense variant rs3740691 in gene ARFGAP2 predicts short-term nonresponse to anti-TNF inhibitor adalimumab in Crohn's disease patients. *Eur J Hum Genet*. 2019;27(0):1670.
4. Barber GE, Yajnik V, Khalili H, et al. Genetic Markers Predict Primary Non-Response and Durable Response to Anti-TNF Biologic Therapies in Crohn's Disease. *Am J Gastroenterol*. 2016;111(12):1816-1822. doi:10.1038/ajg.2016.408
5. Peter Hoffmann , David Lamerz , Petra Hill MK and AG. Gene Polymorphisms of NOD2, IL23R, PTPN2 and ATG16L1 in Patients with Crohn's Disease: On theWay to Personalized Medicine? *Genes (Basel)*. 2021;12(6):866.
6. Marla C Dubinsky, Ling Mei, Madison Friedman, Tanvi Dhere, Talin Haritunians, Hakon Hakonarson, Cecilia Kim, Joseph Glessner, Stephan R Targan DPM, Kent D Taylor and JIR. Genome Wide Association (GWA) Predictors Of Anti-TNFα Therapeutic Responsiveness In Pediatric Inflammatory Bowel Disease (IBD). *AIDS Behav*. 2012;23(1):1031-1043. doi:10.1002/ibd.21174.Genome

7. Netz U, Carter JV, Eichenberger MR, et al. Genetic polymorphisms predict response to anti-tumor necrosis factor treatment in Crohn's disease. *World J Gastroenterol*. 2017;23(27):4958-4967. doi:10.3748/wjg.v23.i27.4958
8. Dezelak M, Repnik K, Koder S, Ferkolj I, Potočnik U. A Prospective Pharmacogenomic Study of Crohn's Disease Patients during Routine Therapy with Anti-TNF-α Drug Adalimumab: Contribution of ATG5, NFKB1, and CRP Genes to Pharmacodynamic Variability. *Omi A J Integr Biol*. 2016;20(5):296-309. doi:10.1089/omi.2016.0005
9. Koder S, Repnik K, Ferkolj I, Pernat C, , Pavel Skok1, 2 RK, Potočnik W& U. Genetic polymorphism in ATG16L1 gene influences the response to adalimumab in Crohn's disease patients. *Pharmacogenomics*. 2015;16(3):191-204.
10. Zhang C Bin, Tang J, Wang XD, Lyu KS, Huang M, Gao X. Multi-alleles predict primary non-response to infliximab therapy in Crohn's disease. *Gastroenterol Rep*. 2021;9(5):427-434. doi:10.1093/gastro/goaa070
11. Hlavaty T, Pierik M, Henckaerts L, et al. Polymorphisms in apoptosis genes predict response to infliximab therapy in luminal and fistulizing Crohn's disease. *Aliment Pharmacol Ther*. 2005;22(7):613-626. doi:10.1111/j.1365-2036.2005.02635.x
12. Duricova D, Pedersen N, Lenicek M, et al. Infliximab dependency in children with Crohn's disease. *Aliment Pharmacol Ther*. 2009;29(7):792-799. doi:10.1111/j.1365-2036.2009.03926.x
13. Barreiro-de Acosta M, Ouburg S, Morré SA, et al. NOD2, CD14 and TLR4 mutations do not influence response to adalimumab in patients with Crohn's disease: A preliminary report. *Rev Esp Enfermedades Dig*. 2010;102(10):591-595. doi:10.4321/S1130-01082010001000005
14. Bank S, Andersen PS, Burisch J, et al. Associations between functional polymorphisms in the NFκB signaling pathway and response to anti-TNF treatment in Danish patients with inflammatory bowel disease. *Pharmacogenomics J*. 2014;14(6):526-534. doi:10.1038/tpj.2014.19
15. Thomas D, Gazouli M, Karantanos T, et al. Association of rs1568885, rs1813443 and rs4411591 polymorphisms with anti-TNF medication response in Greek patients with Crohn's disease. *World J Gastroenterol*. 2014;20(13):3609-3614. doi:10.3748/wjg.v20.i13.3609
16. Walczak M, Lykowska-Szuber L, Plucinska M, et al. Is Polymorphism in the Apoptosis and Inflammatory Pathway Genes Associated With a Primary Response to Anti-TNF Therapy in Crohn's Disease Patients? *Front Pharmacol*. 2020;11(August). doi:10.3389/fphar.2020.01207
17. Curci D, Lucafò M, Cifù A, et al. Pharmacogenetic variants of infliximab response in young patients with inflammatory bowel disease. *Clin Transl Sci*. 2021;14(6):2184-2192. doi:10.1111/cts.13075
18. Louis E, El Ghoul Z, Vermeire S, et al. Association between polymorphism in IgG Fc receptor IIIa coding gene and biological response to infliximab in Crohn's disease. *Aliment Pharmacol Ther*. 2004;19(5):511-519. doi:10.1111/j.1365-2036.2004.01871.x
19. Matsuoka K, Hamada S, Shimizu M, et al. Factors predicting the therapeutic response to infliximab during maintenance therapy in Japanese patients with Crohn's disease. *PLoS One*. 2018;13(10):1-10. doi:10.1371/journal.pone.0204632
20. Papamichaela K, Gazoulib M, Karakoidasa C, Panayotouc I, Roma-Giannikouc E, Mantzarisa GJ. Association of TNF and FcγRIIA gene polymorphisms with differential response to infliximab in a Greek cohort of crohn's disease patients. *Ann Gastroenterol*. 2011;24(1):35-40.
21. Louis EJ, Watier HE, Schreiber S, et al. Polymorphism in IgG Fc receptor gene FCGR3A and response to infliximab in Crohn's disease: A subanalysis of the ACCENT I study. *Pharmacogenet Genomics*. 2006;16(12):911-914. doi:10.1097/01.fpc.0000230421.12844.fd
22. Jung ES, Choi K woon, Kim SW, et al. ZNF133 is associated with infliximab responsiveness in patients with inflammatory bowel diseases. *J Gastroenterol Hepatol*. 2019;34(10):1727-1735. doi:10.1111/jgh.14652
23. Repnik K, Koder S, Skok P, Ferkolj I, Potočnik U. Transferrin Level Before Treatment and Genetic Polymorphism in HFE Gene as Predictive Markers for Response to Adalimumab in Crohn's Disease Patients. *Biochem Genet*. 2016;54(4):476-486. doi:10.1007/s10528-016-9734-0
24. Urcelay E, Mendoza JL, Martínez A, et al. IBD5 polymorphisms in inflammatory bowel disease: Association with response to infliximab. *World J Gastroenterol*.

2005;11(8):1187-1192. doi:10.3748/wjg.v11.i8.1187
25. Bank S, Andersen PS, Burisch J, et al. Genetically determined high activity of IL-12 and IL-18 in ulcerative colitis and TLR5 in Crohns disease were associated with non-response to anti-TNF therapy. *Pharmacogenomics J*. 2018;18(1):87-97. doi:10.1038/tpj.2016.84
26. Salvador-Martín S, Bossacoma F, Pujol-Muncunill G, et al. Genetic Predictors of Long-term Response to Antitumor Necrosis Factor Agents in Pediatric Inflammatory Bowel Disease. *J Pediatr Gastroenterol Nutr*. 2020;71(4):508-515. doi:10.1097/MPG.0000000000002840
27. Repnik K.; Koder S.; Ferkolj I.; Potocnik U.; Cross disease pharmacogenetic analysis predicting anti-TNF response identifies SNPs predicting adalimumab response in Crohn’s disease patients. *Eur J Hum Genet*. 2019;26(0):680.
28. Urabe S, Isomoto H, Ishida T, et al. Genetic Polymorphisms of IL-17F and TRAF3IP2 Could Be Predictive Factors of the Long-Term Effect of Infliximab against Crohn’s Disease. *Biomed Res Int*. 2015;2015. doi:10.1155/2015/416838
29. Bank S, Julsgaard M, Abed OK, et al. Polymorphisms in the NFkB, TNF-alpha, IL-1beta, and IL-18 pathways are associated with response to anti-TNF therapy in Danish patients with inflammatory bowel disease. *Aliment Pharmacol Ther*. 2019;49(7):890-903. doi:10.1111/apt.15187
30. Lacruz-Guzmán D, Torres-Moreno D, Pedrero F, et al. Influence of polymorphisms and TNF and IL1β serum concentration on the infliximab response in Crohn’s disease and ulcerative colitis. *Eur J Clin Pharmacol*. 2013;69(3):431-438. doi:10.1007/s00228-012-1389-0
31. Naviglio S, Stocco G, Cuzzoni E, et al. Pharmacogenetic determinants of response to infliximab in pediatric inflammatory bowel disease. *Dig Liver Dis*. 2015;47:e264. doi:10.1016/j.dld.2015.07.123
32. K. M, H. I, T. I, et al. Genetic polymorphism in the TLR4 related gene can be a predictive factor of long-term effect of infliximab treatment for Crohn’s disease. *United Eur Gastroenterol J*. 2015;3(5 SUPPL. 1):A234. http://ovidsp.ovid.com/ovidweb.cgi?T=JS&PAGE=reference&D=emed17&NEWS=N&AN=72266336
33. Nuij VJAA, Peppelenbosch MP, Woude CJ, Fuhler GM. Genetic polymorphism in ATG16L1 gene is associated with adalimumab use in inflammatory bowel disease. *J Transl Med*. 2017;15(1):1-8. doi:10.1186/s12967-017-1355-9
34. Meggyesi N, Kiss LS, Koszarska M, et al. NKX2-3 AND IRGM variants are associated with disease susceptibility to IBD in Eastern European patients. *World J Gastroenterol*. 2010;16(41):5233-5240. doi:10.3748/wjg.v16.i41.5233
35. Wang MH, Friton JJ, Raffals LE, et al. Novel Genetic Risk Variants Can Predict Anti-TNF Agent Response in Patients with Inflammatory Bowel Disease. *J Crohn’s Colitis*. 2019;13(8):1036-1043. doi:10.1093/ecco-jcc/jjz017
36. Spalinger MR, Voegelin M, Biedermann L, et al. The clinical relevance of the IBD-associated variation within the risk gene locus encoding protein tyrosine phosphatase non-receptor type 2 in patients of the Swiss IBD cohort. *Digestion*. 2016;93(3):182-192. doi:10.1159/000444479
37. Salvador-Martín S, López-Cauce B, Nuñez O, et al. Genetic predictors of long-term response and trough levels of infliximab in crohn’s disease. *Pharmacol Res*. 2019;149(September):104478. doi:10.1016/j.phrs.2019.104478
38. Dideberg V, Théâtre E, Farnir F, et al. The TNF/ADAM 17 system: Implication of an ADAM 17 haplotype in the clinical response to infliximab in Crohn’s disease. *Pharmacogenet Genomics*. 2006;16(10):727-734. doi:10.1097/01.fpc.0000230117.26581.a4
39. López-Hernández R, Valdés M, Campillo JA, et al. Genetic polymorphisms of tumour necrosis factor alpha (TNF-α) promoter gene and response to TNF-α inhibitors in Spanish patients with inflammatory bowel disease. *Int J Immunogenet*. 2014;41(1):63-68. doi:10.1111/iji.12059
40. Louis E, Vermeire S, Rutgeerts P, et al. Inflammatory Bowel Disease A Positive Response to Infliximab in Crohn Disease: Association with a Higher Systemic Inflammation Before Treatment But Not With -308 TNF Gene Polymorphism. *Scand J Gastroenterol*. 2002;37(7):818-824. doi:10.1080/gas.37.7.818.824
41. Medrano LM, Taxonera C, González-Artacho C, et al. Response to infliximab in Crohn’s disease: Genetic analysis supporting expression profile. *Mediators Inflamm*. 2015;2015. doi:10.1155/2015/318207
42. Billiet, T.; Cleynen, I.; Ballet, V.; Ferrante, M.; Rutgeerts, P.; Vermeire S. Primary response to infliximab in Crohn’s disease is associated with the TNFRSF1A

rs1800693 gene polymorphism. *J Crohn’s Colitis*. 2013;7(0):282.

43. Matsukura H, Ikeda S, Yoshimura N, Takazoe M, Muramatsu M. Genetic polymorphisms of tumour necrosis factor receptor superfamily 1A and 1B affect responses to infliximab in Japanese patients with Crohn’s disease. *Aliment Pharmacol Ther*. 2008;27(9):765-770. doi:10.1111/j.1365-2036.2008.03630.x
44. Medrano LM, Taxonera C, Márquez A, et al. Role of TNFRSF1B polymorphisms in the response of Crohn’s disease patients to infliximab. *Hum Immunol*. 2014;75(1):71-75. doi:10.1016/j.humimm.2013.09.017
45. Mascheretti S, Hampe J, Kühbacher T, et al. Pharmacogenetic investigation of the TNF/TNF-receptor system in patients with chronic active Crohn’s disease treated with infliximab. *Pharmacogenomics J*. 2002;2(2):127-136. doi:10.1038/sj.tpj.6500091
46. Steenholdt C, Enevold C, Ainsworth MA, Brynskov J, Thomsen OO, Bendtzen K. Genetic polymorphisms of tumour necrosis factor receptor superfamily 1b and fas ligand are associated with clinical efficacy and/or acute severe infusion reactions to infliximab in Crohn’s disease. *Aliment Pharmacol Ther*. 2012;36(7):650-659. doi:10.1111/apt.12010
