# Supplementary material for: The Association between Genetics and Response to Treatment with Biologics in Patients with Psoriasis, Psoriatic Arthritis, Rheumatoid Arthritis, and Inflammatory Bowel Diseases: A Systematic Review and Meta-Analysis
Source: Int J Mol Sci. 2024 May 26;25(11):5793. doi: 10.3390/ijms25115793 (PMC11171831; doi:10.3390/ijms25115793)
Supplement: Supplementary file 1 [file ijms-25-05793-s001.zip › Supplementary Table S6.pdf]

Supplementary Table S6. Pooled odds ratio using random effects meta-analysis for psoriasis

Gene (rs number)	Drug	Studies (n)	Patients (n)	Odds ratio (95% CI)#	I2 (95% CI)
**5-HTR2A** (rs6311)	TNFi-combined	2	144[1]+68[2] =212	**0.18 (0.06 – 0.58)***	NA
**C17orf51** (rs1975974)	TNFi-combined	2	144[1]+78[2] =222	**3.73 (1.81 – 7.73)***	NA
**CDKAL1** (rs6908425)	TNFi-overall	3	144[1] + 96[2] + 116[3] =356	1.24 (0.29 – 5.35)	86% (34.7% to 93.6%)
**CTNNA2** (rs11126740)	TNFi-combined	2	144[1]+78[2] =222	**0.16 (0.06 – 0.42)***	NA
**FCGR2A** (rs1801274)	TNFi-overall	4	100[4]+70[5]+144[1] + 30[6] =344	1.17 (0.47 – 2.91)	49% (0% to 81.6%)
FCGR2A *(rs1801274)*	*Etanercept*	*2*	*55*[4] + *30*[6] *=85*	*0.75 (0.27 – 2.13)*	*0% (NA)*
**FCGR3A** (rs396991)	TNFi-overall	3	100[4]+56[5] + 115[6] =271	0.81 (0.16 – 4.06)	73.3% (0% to 89.9%)
FCGR3A *(rs396991)*	*Etanercept*	*2*	*55*[4] + *30*[6] *=85*	*1.60 (0.02 – 131.5)*	*92.3% (NA)*
**GBP6** (rs928655)	TNFi-combined	2	144[1]+68[2] =212	**3.50 (1.57 – 7.78)***	NA
**IFIH1** (rs17716942)	TNFi-combined	2	144[1]+282[7] =426	0.62 (0.22 – 1.79)	71.5% (NA)
**IL-12B** (rs2546890)	TNFi-combined	2	68[2]+144[1] =212	**0.37 (0.17 – 0.81)***	NA
**IL-12B** (rs3213094)	TNFi-combined	2	80[8]+280[7] =360	1.51 (0.88 – 2.60)	NA
**IL-12B** (rs6887695)	TNFi-combined	3	144[1]+80[8]+248[9] =472	1.55 (0.57 – 4.24)	68% (0% to 88.6%)
**IL-17A** (rs2275913)	TNFi-overall	3	143[10]+132[11]+ 249[9] =524	1.35 (0.87 – 2.08)	0% (0%-72.9%)
**IL-19** (rs2243188)	TNFi-combined	2	95[12]+144[1] =239	**0.41 (0.22 – 0.76)***	NA
**IL-23R** (rs11209026)	TNFi-combined	3	102[13]+250[9]+272[7] =624	0.90 (0.44 – 1.86)	0% (0%-72.9%)
IL-23R *(rs11209026)*	*Adalimumab*	*2*	*28*[13]+*121*[7] *=149*	*1.16 (0.26 – 4.35)*	*NA*
IL-23R *(rs11209026)*	*Etanercept*	*2*	*54*[13]+*159*[7] *=213*	*1.11 (0.33 – 3.78)*	*NA*
**MAP3K1** (rs96844)	TNFi-overall	2	95[12]+144[1] =239	**0.22 (0.10 – 0.47)***	NA
**MYD88** (rs7744)	TNFi-overall	2	144[1]+249[9] =393	2.42 (0.54 – 10.88)	52.9% (NA)
**NFKB1** (rs28362491)	TNFi-combined	2	96[14]+249[9] =345	0.79 (0.39 – 1.61)	45.4% (NA)
**NFKBIA** (rs696)	TNFi-combined	2	96[14]+247[9]=343	1.29 (0.46 – 3.61)	72,8% (NA)
**PSTP1P1** (rs2254441)	TNFi-combined	2	144[1]+68[2] =212	**5.82 (1.34 – 25.29)***	NA
**PTTG1** (rs2431697)	TNFi-overall	2	144[1]+95[12] =239	**0.43 (0.20 – 0.95)***	NA
**TNF-α-238** (rs361525)	TNFi-combined	3	102[13]+97[15]+249[9] =448	0.58 (0.14 – 2.43)	78.9% (0% to 91.4%)
TNF-α-238 *(rs361525)*	*Etanercept*	*2*	*97*[15]+*54*[13] *=151*	***0.19 (0.08 – 0.47)****	*NA*
**TNF-α-308** (rs1800629)	TNFi-combined	4	102[13]+97[15]+249[9]+100[16] =548	**0.41 (0.19 – 0.88)***	47.3% (0% to 81.1%)
TNF-α-308 *(rs1800629)*	*Etanercept*	*2*	*97*[15] + *100*[16] = *197*	***0.21 (0.05 – 0.80)****	*43.7% (NA)*
**TNF-α-857** (rs1799724)	TNFi-combined	4	80[17]+102[13]+97[15]+49[18] =328	**0.53 (0.29 – 0.97)***	15.6% (0% to 72.6%)
TNF-α-857 *(rs1799724)*	*Etanercept*	*3*	*97*[15]+*54*[13]+*44*[17] *=195*	*0.55 (0.06 – 5.50)*	*81.4% (0% to 92.2%)*
**TNFAIP3** (rs610604)	TNFi-combined	5	189[19]+243[7]+95[12]+49[18]+100[16] =676	0.70 (0.30 – 1.60)	66.3% (0% to 85%)

TNFAIP3 *(rs610604)*	*Adalimumab*	*2*	*40*[19]+*121*[7] =*161*	*1.52 (0.28 – 8.32)*	*73.7% (= *% to *%)*
TNFAIP3 *(rs610604)*	*Etanercept*	*3*	*100*[16]+*159*[7]+*35*[19] =*294*	*0.68 (0.35 – 1.32)*	*0% (0% to 72.9%)*
**TNFRSF1A** (rs191190)	TNFi-overall	2	144[1]+95[12] =239	**2.92 (1.28 – 6.63)***	NA
**TNFRSF1A** (rs4149570)	TNFi-overall	2	144[1]+249[9] =393	**0.51 (0.29 – 0.89)***	0% (NA)
**TNFRSF1B** (rs1061622)	TNFi-overall	6	80[17]+90[20]+144[1]+95[12]+49[18]+100[16] =558	**0.49 (0.30 – 0.79)***	20.2% (0% to 68.4%)
**TRAF3IP2** (rs240993)	TNFi-combined	2	278[7] +74[8] =352	1.61 (0.96 – 2.70)	NA
**ZNF816A** (rs9304742)	TNFi-overall	2	144[1]+95[12] =239	**0.33 (0.17 – 0.64)***	NA
**CHUK** (rs11591741)	IL12/23i	2	230[9]+69[21] =299	0.88 (0.06 – 13.99)	92.2% (NA)
**IL-17A** (rs2275913)	IL12/23i	2	66[7]+142[9] =208	0.58 (0.29 – 1.17)	NA
**IL-23R** (rs11209026)	IL12/23i	2	66[7]+142[9] =208	0.51 (0.13 – 1.97)	NA
**TNFAIP3** (rs610604)	IL12/23i	2	51[22]+66[7] =117	0.54 (0.20 – 1.46)	12.4% (NA)

NA, not available; n, number. TNFi combined: includes studies assessing TNFi overall but also individual drugs. TNFi overall: includes only studies assessing TNFi overall as a drug class. *p<0.05
#odds ratio (OR) of response comparing minor allele with major allele, OR>1 favors major allele, OR<1 favors minor allele.

1. Prieto-Pérez, R.; Solano-López, G.; Cabaleiro, T.; Román, M.; Ochoa, D.; Talegón, M.; Baniandrés, O.; López-Estebaranz, J.L.; De La Cueva, P.; Daudén, E.; et al. New Polymorphisms Associated with Response to Anti-TNF Drugs in Patients with Moderate-to-Severe Plaque Psoriasis. *Pharmacogenomics Journal* **2018**, *18*, 70–75, doi:10.1038/tpj.2016.64.
2. Ovejero-Benito, M.C.; Prieto-Pérez, R.; Llamas-Velasco, M.; Carmen Belmonte, T.C.; Manuel; Román1, D.O.; María Talegón1, M.S.-R.; Esteban; Daudén‡, 2; Francisco, &; et al. Polymorphisms Associated with Etanercept Response in Moderate-to-Severe Plaque Psoriasis. *Pharmacogenomics* **2017**, *18*, 631–638.
3. Coto-Segura, P.; Batalla, A.; González-Fernández, D.; Gómez, J.; Santos-Juanes, J.; Queiro, R.; Alonso, B.; Iglesias, S.; Coto, E. CDKAL1 Gene Variants Affect the Anti-TNF Response among Psoriasis Patients. *International Immunopharmacology* **2015**, *29*, 947–949, doi:10.1016/j.intimp.2015.11.008.
4. Mendrinou, E.; Patsatsi, A.; Zafiriou, E.; Papadopoulou, D.; Aggelou, L.; Sarri, C.; Mamuris, Z.; Kyriakou, A.; Sotiriadis, D.; Roussaki-Schulze, A.; et al. FCGR3A-V158F Polymorphism Is a Disease-Specific Pharmacogenetic Marker for the Treatment of Psoriasis with Fc-Containing TNFα Inhibitors. *Pharmacogenomics Journal* **2017**, *17*, 237–241, doi:10.1038/tpj.2016.16.
5. Julià, M.; Guilabert, A.; Lozano, F.; Suarez-Casasús, B.; Moreno, N.; Carrascosa, J.M.; Ferrándiz, C.; Pedrosa, E.; Alsina-Gibert, M.; Mascaró, J.M. The Role of Fcγ Receptor Polymorphisms in the Response to Anti-Tumor Necrosis Factor Therapy in Psoriasis: A Pharmacogenetic Study. *JAMA Dermatol* **2013**, *149*, 1033–1039, doi:10.1001/jamadermatol.2013.4632.
6. Batalla, A.; Coto, E.; Coto-Segura, P. Influence of Fcγ Receptor Polymorphisms on Response to Anti-Tumor Necrosis Factor Treatment in Psoriasis. *JAMA Dermatol* **2015**, *151*, 1376–1378, doi:10.1001/jamadermatol.2015.2818.
7. van den Reek, J.M.P.A.; Coenen, M.J.H.; van de L'Isle Arias, M.; Zweegers, J.; Rodijk-Olthuis, D.; Schalkwijk, J.; Vermeulen, S.H.; Joosten, I.; van de Kerkhof, P.C.M.; Seyger, M.M.B.; et al. Polymorphisms in CD84, IL12B and TNFAIP3 Are Associated with Response to Biologics in Patients with Psoriasis. *British Journal of Dermatology* **2017**, *176*, 1288–1296, doi:10.1111/bjd.15005.

8. Torii, K.; Morita, A. Specific Single Nucleotide Polymorphism Genotypes and Association of an IL-12B Polymorphism with Secondary Failure of Infliximab Therapy in Japanese Psoriasis Patients. *Journal of Dermatological Science* **2020**, *99*, 135–136, doi:10.1016/j.jdermsci.2020.05.011.
9. N.D., L.; L., S.; L., I.; R., G.; T.N., D.; I., B.; H.J., H.; M.R., A.; R.B., D.; A.C., B.; et al. Associations between Functional Polymorphisms and Response to Biological Treatment in Danish Patients with Psoriasis. *Pharmacogenomics Journal* **2018**, *18*, 494–500, doi:https://dx.doi.org/10.1038/tpj.2017.31 PT - Article.
10. Prieto-Pérez R, Solano-López, C.T. et al The Polymorphism Rs763780 in the IL-17F Gene Is Associated with Response to Biological Drugs in Patients with Psoriasis. *Pharmacogenomics* **2015**, *16*, 1723–1731.
11. van Vugt, L.J.; van den Reek, J.M.P.A.; Meulewaeter, E.; Hakobjan, M.; Heddes, N.; Traks, T.; Kingo, K.; Galluzzo, M.; Talamonti, M.; Lambert, J.; et al. Response to IL-17A Inhibitors Secukinumab and Ixekizumab Cannot Be Explained by Genetic Variation in the Protein-Coding and Untranslated Regions of the IL-17A Gene: Results from a Multicentre Study of Four European Psoriasis Cohorts. *Journal of the European Academy of Dermatology and Venereology* **2020**, *34*, 112–118, doi:10.1111/jdv.15787.
12. María Carmen Ovejero-Benito, Rocío Prieto-Pérez, Mar Llamas-Velasco, Ester Muñoz-Aceituno, Alejandra Reolid, Miriam Saiz-Rodríguez, Carmen Belmonte, Manuel Román, Dolores Ochoa, María Talegón, Teresa Cabaleiro, Esteban Daudén, F.A.-S. Polymorphisms Associated with Adalimumab and Infliximab Response in Moderate- to-Severe Plaque Psoriasis. *Pharmacogenomics* **2018**, *19*, 7–16.
13. Gallo, E.; Cabaleiro, T.; Román, M.; Solano-López, G.; Abad-Santos, F.; García-Díez, A.; Daudén, E. The Relationship between Tumour Necrosis Factor (TNF)-α Promoter and IL12B/IL-23R Genes Polymorphisms and the Efficacy of Anti-TNF-α Therapy in Psoriasis: A Case-Control Study. *British Journal of Dermatology* **2013**, *169*, 819–829, doi:10.1111/bjd.12425.
14. Caldarola, G.; Sgambato, A.; Fanali, C.; Moretta, G.; Farina, M.; Lucchetti, D.; Peris, K.; De Simone, C. HLA-Cw6 Allele, NFkB1 and NFkBIA Polymorphisms Play No Role in Predicting Response to Etanercept in Psoriatic Patients. *Pharmacogenet Genomics* **2016**, *26*, 423–427, doi:10.1097/FPC.0000000000000233.

15. De Simone, C.; Farina, M.; Maiorino, A.; Fanali, C.; Perino, F.; Flamini, A.; Caldarola, G.; Sgambato, A. TNF-Alpha Gene Polymorphisms Can Help to Predict Response to Etanercept in Psoriatic Patients. *Journal of the European Academy of Dermatology and Venereology* **2015**, *29*, 1786–1790, doi:10.1111/jdv.13024.
16. Hassan Hadi, A.M.; Abbas, A.A.-H.; Abdulamir, A.S.; Fadheel, B.M. The Effect of TnFaip3 Gene Polymorphism on Disease Susceptibility and Response of Etanercept in Psoriatic Patients. *European Journal of Molecular and Clinical Medicine* **2020**, *7*, 240–246.
17. Vasilopoulos, Y.; Manolika, M.; Zafiriou, E.; Sarafidou, T.; Bagiatis, V.; Krüger-Krasagaki, S.; Tosca, A.; Patsatsi, A.; Sotiriadis, D.; Mamuris, Z.; et al. Pharmacogenetic Analysis of TNF, TNFRSF1A, and TNFRSF1B Gene Polymorphisms and Prediction of Response to Anti-TNF Therapy in Psoriasis Patients in the Greek Population. *Mol Diagn Ther* **2012**, *16*, 29–34, doi:10.2165/11594660.
18. Ito, M.; Hirota, T.; Momose, M.; Ito, T.; Umezawa, Y.; Fukuchi, O.; Asahina, A.; Nakagawa, H.; Tamari, M.; Saeki, H. Lack of Association of TNFA, TNFRSF1B and TNFAIP3 Gene Polymorphisms with Response to Anti-Tumor Necrosis Factor Therapy in Japanese Patients with Psoriasis. *Journal of Dermatology* **2020**, *47*, e110–e111, doi:10.1111/1346-8138.15200.
19. Masouri, S.; Stefanaki, I.; Ntritsos, G.; Kypreou, K.P.; Drakaki, E.; Evangelou, E.; Nicolaidou, E.; Stratigos, A.J.; Antoniou, C. A Pharmacogenetic Study of Psoriasis Risk Variants in a Greek Population and Prediction of Responses to Anti-TNF-α and Anti-IL-12/23 Agents. *Molecular Diagnosis and Therapy* **2016**, *20*, 221–225, doi:10.1007/s40291-016-0198-z.
20. González-Lara, L.; Batalla, A.; Coto, E.; Gómez, J.; Eiris, N.; Santos-Juanes, J.; Queiro, R.; Coto-Segura, P. The TNFRSF1B Rs1061622 Polymorphism (p.M196R) Is Associated with Biological Drug Outcome in Psoriasis Patients. *Arch Dermatol Res* **2015**, *307*, 405–412, doi:10.1007/s00403-014-1533-z.
21. Prieto-Pérez, R.; Mar; Llamas-Velasco; Teresa; Cabaleiro, Guillermo Solano-López, B.M.; Román1, M.; Ochoa1, D.; María; Talegón1; Daudén2, E.; et al. Pharmacogenetics of Ustekinumab in Patients with Moderate-to-Severe Plaque Psoriasis. *Pharmacogenetics* **2017**, *18*, 157–164.

22. Talamonti, M.; Botti, E.; Galluzzo, M.; Teoli, M.; Spallone, G.; Bavetta, M.; Chimenti, S.; Costanzo, A. Pharmacogenetics of Psoriasis: HLA-Cw6 but Not LCE3B/3C Deletion nor TNFAIP3 Polymorphism Predisposes to Clinical Response to Interleukin 12/23 Blocker Ustekinumab. *British Journal of Dermatology* **2013**, *169*, 458–463, doi:10.1111/bjd.12331.
