# Supplementary material for: The Association between Genetics and Response to Treatment with Biologics in Patients with Psoriasis, Psoriatic Arthritis, Rheumatoid Arthritis, and Inflammatory Bowel Diseases: A Systematic Review and Meta-Analysis
Source: Int J Mol Sci. 2024 May 26;25(11):5793. doi: 10.3390/ijms25115793 (PMC11171831; doi:10.3390/ijms25115793)
Supplement: Supplementary file 1 [file ijms-25-05793-s001.zip › Supplementary Table S7.pdf]

| **Supplementary Table S7**. Pooled odds ratio using random effects meta-analysis for rheumatoid arthritis | | | | | |
|---|---|---|---|---|---|
| **Gene (rs number)** | **Drug** | **Studies (n)** | **Patients (n)** | **Odds ratio (95% CI)#** | **I2 (95% CI)** |
| **C9orf72** (rs3849942) | TNFi-overall | 3 | 95[1] + 135[2] + 89[3] =319 | **0.50 (0.27 – 0.93)*** | 39.4% (0% to 82.2%) |
| **CARD8** (rs10403848) | TNFi-overall | 2 | 1283[4]+289[5] =1572 | 1.13 (0.79 – 1.61) | 49.8% (NA) |
| **CARD8** (rs11672725) | TNFi-overall | 2 | 1283[4]+532[5] =1815 | 1.11 (0.88 – 1.41) | NA |
| **CENTD1** (rs437943) | TNFi-overall | 3 | 135[2]+89[3] + 95[1] =319 | 0.77 (0.22 – 2.80) | 77 % (0% to 90.9%) |
| **CHUK** (rs11591741) | TNFi-overall | 4 | 883[6]+183[7]+ 689[8]+971[9] =2726 | 0.89 (0.74 – 1.08) | 0% (0% to 67.9%) |
| **FCGR2A** (rs1801274) | TNFi-combined | 5 | 202[10] + 302[11] + 291[12] + 85[13] + 348[14] =1228 | 1.03 (0.63 – 1.68) | 61.3% (0% to 83.3%) |
| **FCGR3A** (rs396991) | TNFi-combined | 8 | 301[11] + 377[15] + 37[16] + 77[13] + 282[17] +78[18] + 36[19] + 33[20]= 1221 | **0.77 (0.59 – 0.99)*** | 6.5% (0% to 59%) |
| ***FCGR3A*** *(rs396991)* | *Infliximab* | *4* | *37*[16] + *78*[18] + *77*[13] + *29*[19] *=144* | *0.78 (0.42 – 1.41)* | *87.7% (NA)* |
| **GBP6** (rs928655) | TNFi-overall | 3 | 135[2]+89[3] + 95[1] = 319 | 1.50 (0.43 – 5.31) | 79.5% (0% to 91.6%) |
| **IFNK** (rs7046653) | TNFi-overall | 3 | 135[2]+89[3] + 95[1] =319 | **0.60 (0.37 – 0.99)*** | 46.5% (0% to 83.8%) |
| **IKBKB** (rs11986055) | TNFi-overall | 2 | 901[6]+986[9] =1887 | 1.42 (0.40 – 5.07) | 83% (NA) |
| **IL-6** (rs1800795) | TNFi-combined | 2 | 77[21] + 191[22] = 268 | 0.76 (0.38 – 1.51) | 0 (NA) |
| **IL-10** (rs1800896) | TNFi-combined | 4 | 689[8] + 113[23] + 149[24] + 15[25] =966 | 1.12 (0.75 – 1.67) | 13.5% (5 to 72%) |
| **IRAK-3** (rs11541076) | TNFi-overall | 2 | 901[6]+987[9] =1888 | **1.38 (1.09 – 1.75)*** | NA |
| **LASS6** (rs13393173) | TNFi-overall | 3 | 89[3]+135[2] + 95[1] =319 | 0.61 (0.22 – 1.63) | 65.3% (0% to 88%) |
| **LMO4** (rs983332) | TNFi-overall | 3 | 89[3]+135[2] + 95[1] =319 | 0.54 (0.21 – 1.40) | 70.6% (0% to 89.2%) |
| **LOC105369309** (rs717117) | TNFi-overall | 2 | 532[5]+135[2] =667 | 0.26 (0.04 – 1.83) | 84.7% (NA) |
| **MAP2K6** (rs11656130) | TNFi-overall | 2 | 918[26] + 689[8] =1607 | 0.82 (0.46 – 1.45) | 83.5% (NA) |
| **MAP2K6** (rs2716191) | TNFi-overall | 2 | 905[26] + 689[8] =1594 | **1.21 (1.01 – 1.44)*** | NA |
| **MAPK14** (rs916344) | TNFi-overall | 2 | 918[26] + 689[8] =1607 | 1.02 (0.82 – 1.27) | NA |
| **MAPKAPK2** (rs4240847) | TNFi-overall | 2 | 901[26] + 689[8] =1590 | 0.87 (0.56 – 1.34) | 74.1% (NA) |
| **MOBKL2B** (rs868856) | TNFi-overall | 3 | 89[3]+135[2] + 95[1] =319 | 0.56 (0.27 – 1.16) | 49.2% (0% to 84.3%) |
| **MOBKL2B** (rs2814707) | TNFi-overall | 3 | 89[3]+135[2] + 95[1] =319 | 0.51 (0.25 – 1.01) | 53.2% (0% to 85.2%) |
| **MyD88** (rs7744) | TNFi-overall | 4 | 902[6] + 183[7] + 689[8] + 991[9] = 2765 | 1.20 (0.95 – 1.52) | 29.6% (0% to 76.5%) |
| **NFKBIB** (rs3136645) | TNFi-overall | 2 | 982[9]+902[6] = 1884 | 0.80 (0.56 – 1.14) | 60.5% (NA) |
| **NFKBIB** (rs9403) | TNFi-overall | 2 | 970[9]+893[6] =1863 | 0.79 (0.59 – 1.05) | 42.6% (NA) |
| **NLRP3** (rs4612666) | TNFi-overall | 3 | 516[27]+988[9]+532[5] = 2036 | **0.67 (0.48 – 0.93)*** | 0% (0% to 72.9%) |
| **NLRP3** (rs4925648) | TNFi-overall | 2 | 1283[4] + 289[5] =1572 | 1.28 (0.91 – 1.81) | 26.4% (NA) |
| **NLRP3** (rs10925026) | TNFi-overall | 2 | 1283[4] + 532[5] =1815 | **0.74 (0.56 – 0.97)*** | 0% (NA) |
| **PADI4** (rs2240340) | TNFi-combined | 2 | 208[28] + 291[12] =499 | 1.12 (0.57 – 2.19) | 84.1% (NA) |
| **PTPRC** (rs10919563) | TNFi-overall | 5 | 183[7] + 469[29] + 208[28] + 689[8] +821[30] =2370 | 0.77 (0.56 – 1.05) | 53.5% (0% to 80.9%) |
| **SLCO1C1** (rs3794271) | TNFi-overall | 4 | 135[2]+313[31] + 1750[32]+289[5]=2487 | 0.68 (0.37 – 1.27) | 82.1% (32.9% to 91.3%) |

| | | | | | |
|---|---|---|---|---|---|
| **STAT4** (rs7574865) | TNFi-overall | 2 | 183[7] + 163[33] =346 | 0.65 (0.42 – 1.03) | NA |
| **PON1** (rs854547) | TNFi-overall | 3 | 89[3]+135[2] + 95[1] =319 | 1.47 (0.70 – 3.09) | 30% (0% to 80.2%) |
| **PON1** (rs854548) | TNFi-overall | 3 | 89[3]+135[2] + 95[1] =319 | 0.51 (0.16 – 1.63) | 74.9% (0% to 90.4%) |
| **PON1** (rs854555) | TNFi-overall | 3 | 89[3]+135[2] + 95[1] =319 | 0.64 (0.30 – 1.35) | 47.6% (0% to 84%) |
| **QK1** (rs10945919) | TNFi-overall | 3 | 89[3]+135[2] + 95[1] =319 | 0.69 (0.28 – 1.69) | 63.8% (0% to 87.6%) |
| **RPS6KA4** (rs475032) | TNFi-overall | 2 | 903[26] + 689[8] =1592 | 1.04 (0.78 – 1.39) | 57% (NA) |
| **RPS6KA5** (rs1286076) | TNFi-overall | 2 | 900[26] + 689[8] =1589 | 0.96 (0.76 – 1.22) | 33.4% (NA) |
| **TLR1** (rs4833095) | TNFi-overall | 2 | 511[34]+993[9] =1504 | 0.96 (0.77 – 1.21) | NA |
| **TLR5** (rs5744174) | TNFi-overall | 2 | 411[34]+988[9] =1399 | 1.13 (0.89 – 1.43) | NA |
| **TLR10** (rs11096957) | TNFi-overall | 2 | 894[6]+960[9] =1854 | 0.89 (0.62 – 1.29) | 65.7% (NA) |
| **TNF-α-238** (rs361525) | TNFi-combined | 5 | 70[35]+360[36]+113[37] +476[38]+190[39] =1209 | 0.96 (0.57 – 1.61) | 18.6% (0% to 70.3%) |
| ***TNF-α-238*** *(rs361525)* | *Adalimumab* | *3* | *59*[39] + *360*[36] + *69*[38] = *488* | *1.59 (0.71-3.57)* | *3.8% (0% to 73.9%)* |
| ***TNF-α-238*** *(rs361525)* | *Etanercept* | *3* | *70*[35]+*198*[38]+*102*[39] *=370* | *0.89 (0.33 – 2.39)* | *15.5% (0% to 76.8%)* |
| ***TNF-α-238*** *(rs361525)* | *Infliximab* | *3* | *113*[37]+*209*[38]+*20*[39] *=342* | *0.57 (0.27 – 1.21)* | *0% (0% to 72.9%)* |
| **TNF-α-308** (rs1800629) | TNFi-combined | 12 | 187[39] + 73[40] + 78[41] + 123[23] + 291[12] + 53[42] + 86[42] + 113[37] + 474[38] + 369[36] + 198[43] + 100[44] = 2145 | 0.75 (0.54 – 1.05) | 55.2% (0% to 75%) |
| ***TNF-α-308*** *(rs1800629)* | *Adalimumab* | *5* | 60[39] + *78*[41] + *291*[12] + *71*[38] + *369*[36] = *869* | *0.81 (0.53 – 1.24)* | *48.1% (0% to 79.3%)* |
| ***TNF-α-308*** *(rs1800629)* | *Etanercept* | *5* | *73*[40] + *123*[23] + *86*[42] + *197*[38] + *99*[39] *=578* | *0.63 (0.35 – 1.10)* | *45% (0% to 78.3%)* |
| ***TNF-α-308*** *(rs1800629)* | *Infliximab* | *5* | *53*[45] + *113*[37] + *198*[43] + *19*[39] + *20*[46] = *403* | *0.68 (0.33 – 1.41)* | *35% (0% to 75.4%)* |
| **TNF-α-857** (rs1799724) | TNFi-combined | 4 | 190[39] + 357[36] + 70[35] + 100[44] =717 | 1.41 (0.92 – 2.16) | 14.8% (0% to 72.4%) |
| ***TNF-α-857*** *(rs1799724)* | *Adalimumab* | *2* | *61*[39] + *357*[36] = *418* | *1.66 (0.99 – 2.78)* | *0% (NA)* |
| ***TNF-α-857*** *(rs1799724)* | *Etanercept* | *2* | *101*[39] + *70*[35] = *171* | ***3.05 (1.09 – 8.53)**** | *0% (NA)* |
| **TNFR1A** (rs767455) | TNFi-combined | 3 | 58[47]+89[48]+187[39] =334 | 1.03 (0.63 – 1.70) | 0% (0% to 72.9%) |
| ***TNFR1A*** *(rs767455)* | *Infliximab* | *3* | *58*[47]+*89*[48]+*21*[39] *=168* | *0.84 (0.41 – 1.72)* | *0% (0% to 72.9%)* |
| **TNFRSF1B** (rs1061622) | TNFi-combined | 6 | 212[49]+122[18]+190[39] +15[25]+456[50] + 100[44] =1095 | 0.71 (0.40 – 1.26) | 68.4% (0% to 84.6%) |
| ***TNFRSF1B*** *(rs1061622)* | *Infliximab* | *2* | *122*[18]+*21*[39] *=143* | ***0.25 (0.11 – 0.57)**** | *NA* |
| ***FCGR3A*** *(rs396991)* | *Abatacept* | *2* | *120*[51]+*25*[52] *= 145* | *1.20 (0.51 – 2.79)* | *NA* |
| **TNFSF13B** (rs9514828) | Rituximab | 2 | 115[53]+152[54] = 267 | **0.33 (0.13 – 0.82)*** | NA |
| **FCGR3A** (rs396991) | Rituximab | 5 | 177[55] + 52[56] + 42[57] + 221[58] + 110[59] = 602 | **1.71 (1.16 – 2.51)*** | 0% (0% to 64.1%) |
| **STAT4** (rs7574865) | Rituximab | 2 | 62[60] + 112[61] =174 | 1.24 (0.61 – 2.52) | NA |
| **FCGR2A** (rs1801274) | Tocilizumab | 2 | 154[62] + 87[57] = 241 | 1.19 (0.62 – 2.28) | NA |
| **FCGR3A** (rs396991) | Tocilizumab | 2 | 87[57] + 148[62] =235 | 1.36 (0.60 – 3.06) | NA |

| | | | | | |
|---|---|---|---|---|---|
| **IL-6R** (rs2228145) | Tocilizumab | 2 | 79[63]+77[64] =156 | **10.71 (3.46 – 33.10)*** | NA |
| **IL-6R** (rs4329505) | Tocilizumab | 3 | 79[63]+77[64]+154[62] =310 | 0.87 (0.33 – 2.35) | 59.8% (0% to 86.7%) |
| **IL-6R** (rs12083537) | Tocilizumab | 2 | 77[64]+152[62] =229 | **0.47 (0.24 – 0.89)*** | NA |

NA, not available; n, number. TNFi combined: includes studies assessing TNFi overall but also individual drugs. TNFi overall: includes only studies assessing TNFi overall as a drug class. *p<0.05
#odds ratio (OR) of response comparing minor allele with major allele, OR>1 favors major allele, OR<1 favors minor allele.

1. Suarez-Gestal, M.; Perez-Pampin, E.; Calaza, M.; Gomez-Reino, J.J.; Gonzalez, A. Lack of Replication of Genetic Predictors for the Rheumatoid Arthritis Response to Anti-TNF Treatments: A Prospective Case-Only Study. *Arthritis Res Ther* **2010**, *12*, doi:10.1186/ar2990.
2. Krintel, S.B.; Palermo, G.; Johansen, J.S.; Germer, S.; Essioux, L.; Benayed, R.; Badi, L.; Østergaard, M.; Hetland, M.L. Investigation of Single Nucleotide Polymorphisms and Biological Pathways Associated with Response to TNF$\alpha$ Inhibitors in Patients with Rheumatoid Arthritis. *Pharmacogenet Genomics* **2012**, *22*, 577–589, doi:10.1097/FPC.0b013e3283544043.
3. Liu, C.; Batliwalla, F.; Li, W.; Lee, A.; Roubenoff, R.; Beckman, E.; Khalili, H.; Damle, A.; Kern, M.; Furie, R.; et al. Genome-Wide Association Scan Identifies Candidate Polymorphisms Associated with Differential Response to Anti-TNF Treatment in Rheumatoid Arthritis. *Molecular Medicine* **2008**, *14*, 575–581, doi:10.2119/2008-00056.Liu.
4. Mathews, R.J.; Robinson, J.I.; Battellino, M.; Wong, C.; Taylor, J.C.; Eyre, S.; Churchman, S.M.; Wilson, A.G.; Isaacs, J.D.; Hyrich, K.; et al. Evidence of NLRP3-Inflammasome Activation in Rheumatoid Arthritis (RA); Genetic Variants within the NLRP3-Inflammasome Complex in Relation to Susceptibility to RA and Response to Anti-TNF Treatment. *Annals of the Rheumatic Diseases* **2014**, *73*, 1202–1210, doi:10.1136/annrheumdis-2013-203276.
5. Lopez-Rodriguez, R.; Perez-Pampin, E.; Marquez, A.; Blanco, F.J.; Joven, B.; Carreira, P.; Ferrer, M.A.; Caliz, R.; Valor, L.; Narvaez, J.; et al. Validation Study of Genetic Biomarkers of Response to TNF Inhibitors in Rheumatoid Arthritis. *PLoS One* **2018**, *13*, 1–13, doi:10.1371/journal.pone.0196793.
6. Potter, C.; Cordell, H.J.; Barton, A.; Daly, A.K.; Hyrich, K.L.; Mann, D.A.; Morgan, A.W.; Wilson, A.G.; Isaacs, J.D. Association between Anti-Tumour Necrosis Factor Treatment Response and Genetic Variants within the TLR and NFκB Signalling Pathways. *Ann Rheum Dis* **2010**, *69*, 1315–1320, doi:10.1136/ard.2009.117309.
7. Zervou, M.I.; Myrthianou, E.; Flouri, I.; Plant, D.; Chlouverakis, G.; Castro-Giner, F.; Rapsomaniki, P.; Barton, A.; Boumpas, D.T.; Sidiropoulos, P.; et al. Lack of Association of Variants Previously Associated with Anti-TNF Medication Response in Rheumatoid Arthritis Patients: Results from a Homogeneous Greek Population. *PLoS One* **2013**, *8*, 1–5, doi:10.1371/journal.pone.0074375.
8. Ferreiro-Iglesias, A.; Montes, A.; Perez-Pampin, E.; Cañete, J.D.; Raya, E.; Magro-Checa, C.; Vasilopoulos, Y.; Sarafidou, T.; Caliz, R.; Ferrer, M.A.; et al. Replication of PTPRC as Genetic Biomarker of Response to TNF Inhibitors in Patients with Rheumatoid Arthritis. *Pharmacogenomics Journal* **2016**, *16*, 137–140, doi:10.1038/tpj.2015.29.
9. Sode, J.; Vogel, U.; Bank, S.; Andersen, P.S.; Hetland, M.L.; Locht, H.; Heegaard, N.H.H.; Andersen, V. Confirmation of an IRAK3 Polymorphism as a Genetic Marker Predicting Response to Anti-TNF Treatment in Rheumatoid Arthritis. *Pharmacogenomics Journal* **2018**, *18*, 81–86, doi:10.1038/tpj.2016.66.
10. Montes, A.; Perez-Pampin, E.; Narváez, J.; Cañete, J.D.; Navarro-Sarabia, F.; Moreira, V.; Fernández-Nebro, A.; Del Carmen Ordóñez, M.; De La Serna, A.R.; Magallares, B.; et al. Association of FCGR2A with the Response to Infliximab Treatment of Patients with Rheumatoid Arthritis. *Pharmacogenet Genomics* **2014**, *24*, 238–245, doi:10.1097/FPC.0000000000000042.
11. Dávila-, C.L.; Huizinga, W.; Dávila-Fajardo, C.L.; Van Der Straaten, T.; Baak-Pablo, R.; Medarde Caballero, C.; Cabeza Barrera, J.; Huizinga, T.W.; Guchelaar, H.J.; Swen, J.J. FcGR Genetic Polymorphisms and the Response to Adalimumab in Patients with Rheumatoid Arthritis. *Pharmacogenomics* **2015**, *16*, 373–381.
12. Eektimmerman, F.; Swen, J.; Böhringer, S.; Huizinga, T.W.J.; Wouter, M. Pathway Analysis to Identify Genetic Variants Associated with Efficacy of Adalimumab in Rheumatoid Arthritis. *Pharmacogenomics* **2017**, *18*, 945–953.
13. Cañete, J.D.; Suárez, B.; Hernández, M. V.; Sanmartí, R.; Rego, I.; Celis, R.; Moll, C.; Pinto, J.A.; Blanco, F.J.; Lozano, F. Influence of Variants of Fc$\gamma$ Receptors IIA and IIIA on the American College of Rheumatology and European League Against Rheumatism Responses to Anti-Tumour Necrosis Factor $\alpha$ Therapy in Rheumatoid Arthritis. *Ann Rheum Dis* **2009**, *68*, 1547–1552, doi:10.1136/ard.2008.096982.

14. Avila-Pedretti, G.; Tornero, J.; Fernández-Nebro, A.; Blanco, F.; González-Alvaro, I.; Cañete, J.D.; Maymó, J.; Alperiz, M.; Fernández-Gutiérrez, B.; Olivé, A.; et al. Variation at FCGR2A and Functionally Related Genes Is Associated with the Response to Anti-TNF Therapy in Rheumatoid Arthritis. *PLoS One* **2015**, *10*, 1–12, doi:10.1371/journal.pone.0122088.
15. A., M.; E., P.-P.; B., J.; P., C.; A., F.-N.; M., D.C.O.; F., N.-S.; V., M.; Y., V.; T., S.; et al. FCGR Polymorphisms in the Treatment of Rheumatoid Arthritis with Fc-Containing TNF Inhibitors. *Pharmacogenomics* **2015**, *16*, 333–345.
16. Morales-Lara, M.J.; Conesa-Zamora, P.; Garca-Simón, M.S.; Pedrero, F.; Santaclara, V.; Perez-Guillermo, M.; Soriano-Navarro, E. Association between the FCGR3A V158F Polymorphism and the Clinical Response to Infliximab in Rheumatoid Arthritis and Spondyloarthritis Patients. *Scand J Rheumatol* **2010**, *39*, 518–520, doi:10.3109/03009741003781969.
17. Kastbom, A.; Bratt, J.; Ernestam, S.; Lampa, J.; Padyukov, L.; Söderkvist, P.; Skogh, T. Fc$\gamma$ Receptor Type III A Genotype and Response to Tumor Necrosis Factor $\alpha$-Blocking Agents in Patients with Rheumatoid Arthritis. *Arthritis Rheum* **2007**, *56*, 448–452, doi:10.1002/art.22390.
18. Rooryck, C.; Barnetche, T.; Richez, C.; Laleye, A.; Arveiler, B.; Schaeverbeke, T. Influence of FCGR3A-V212F and TNFRSF1B-M196R Genotypes in Patients with Rheumatoid Arthritis Treated with Infliximab Therapy. *Clin Exp Rheumatol* **2008**, *26*, 340–342.
19. Tsukahara, S.; Ikari, K.; Sato, E.; Yamanaka, H.; Hara, M.; Tomatsu, T.; Momohara, S.; Kamatani, N. A Polymorphism in the Gene Encoding the Fc [GAMMA] IIIA Receptor Is a Possible Genetic Marker to Predict the Primary Response to Infliximab in Japanese Patients with Rheumatoid Arthritis. *Ann Rheum Dis* **2008**, *67*, 1791.
20. Tutuncu, Z.; Kavanaugh, A.; Zvaifler, N.; Corr, M.; Deutsch, R.; Boyle, D. Fc$\gamma$ Receptor Type IIIA Polymorphisms Influence Treatment Outcomes in Patients with Inflammatory Arthritis Treated with Tumor Necrosis Factor $\alpha$–Blocking Agents. *Arthritis Rheum* **2005**, *52*, 2693–2696.
21. Jančić, I.; Arsenović-Ranin, N.; Šefik-Bukilica, M.; Živojinović, S.; Damjanov, N.; Spasovski, V.; Srzentić, S.; Stanković, B.; Pavlović, S. -174G/C Interleukin-6 Gene Promoter Polymorphism Predicts Therapeutic Response to Etanercept in Rheumatoid Arthritis. *Rheumatology International* **2013**, *33*, 1481–1486, doi:10.1007/s00296-012-2586-y.
22. Dávila-Fajardo, C.L.; Márquez, A.; Pascual-Salcedo, D.; Moreno Ramos, M.J.; García-Portales, R.; Magro, C.; Alegre-Sancho, J.J.; Balsa, A.; Cabeza-Barrera, J.; Raya, E.; et al. Confirmation of -174G/C Interleukin-6 Gene Promoter Polymorphism as a Genetic Marker Predicting Antitumor Necrosis Factor Treatment Outcome. *Pharmacogenetics and Genomics* **2014**, *24*, 1–5, doi:10.1097/FPC.0000000000000013.
23. Padyukov, L.; Lampa, J.; Heimbürger, M.; Ernestam, S.; Cederholm, T.; Lundkvist, I.; Andersson, P.; Hermansson, Y.; Harju, A.; Klareskog, L.; et al. Genetic Markers for the Efficacy of Tumour Necrosis Factor Blocking Therapy in Rheumatoid Arthritis. *Ann Rheum Dis* **2003**, *62*, 526–529, doi:10.1136/ard.62.6.526.
24. Canet, L.M.; Cáliz, R.; Lupiañez, C.B.; Canhão, H.; Martinez, M.; Escudero, A.; Filipescu, I.; Segura-Catena, J.; Soto-Pino, M.J.; Ferrer, M.A.; et al. Genetic Variants within Immune-Modulating Genes Influence the Risk of Developing Rheumatoid Arthritis and Anti-TNF Drug Response: A Two-Stage Case-Control Study. *Pharmacogenetics and Genomics* **2015**, *25*, 432–443, doi:10.1097/FPC.0000000000000155.
25. Pers, Y.M.; Cadart, D.; Rittore, C.; Ravel, P.; Daïen, V.; Fabre, S.; Jorgensen, C.; Touitou, I. TNFRII Polymorphism Is Associated with Response to TNF Blockers in Rheumatoid Arthritis Patients Seronegative for ACPA. *Joint Bone Spine* **2014**, *81*, 370–372, doi:10.1016/j.jbspin.2013.12.005.
26. Coulthard, L.R.; Taylor, J.C.; Eyre, S.; Robinson, J.I.; Wilson, A.G.; Isaacs, J.D.; Hyrich, K.; Emery, P.; Barton, A.; Barrett, J.H.; et al. Genetic Variants within the MAP Kinase Signaling Network and Anti-TNF Treatment Response in Rheumatoid Arthritis Patients. *Annals of the Rheumatic Diseases* **2011**, *70*, 98–103, doi:10.1136/ard.2010.133249.

27. Sode, J.; Vogel, U.; Bank, S.; Andersen, P.S.; Thomsen, M.K.; Hetland, M.L.; Locht, H.; Heegaard, N.H.H.; Andersen, V. Anti-TNF Treatment Response in Rheumatoid Arthritis Patients Is Associated with Genetic Variation in the NLRP3-Inflammasome. *PLoS One* **2014**, *9*, 1–10, doi:10.1371/journal.pone.0100361.
28. Canhão, H.; Rodrigues, A.M.; Santos, M.J.; Carmona-Fernandes, D.; Bettencourt, B.F.; Cui, J.; Rocha, F.L.; Canas Silva, J.; Polido-Pereira, J.; Pereira Silva, J.A.; et al. TRAF1/C5 but Not PTPRC Variants Are Potential Predictors of Rheumatoid Arthritis Response to Anti-Tumor Necrosis Factor Therapy. *BioMed Research International* **2015**, *2015*, doi:10.1155/2015/490295.
29. Plant, D.; Prajapati, R.; Hyrich, K.L.; Morgan, A.W.; Wilson, A.G.; Isaacs, J.D.; Barton, A. Replication of Association of the PTPRC Gene with Response to Anti-Tumor Necrosis Factor Therapy in a Large UK Cohort. *Arthritis and Rheumatism* **2012**, *64*, 665–670, doi:10.1002/art.33381.
30. Cui, J.; Saevarsdottir, S.; Thomson, B.; Padyukov, L.; van der Helm-Van Mil, A.H.M.; Nititham, J.; Hughes, L.B.; De Vries, N.; Raychaudhuri, S.; Alfredsson, L. Rheumatoid Arthritis Risk Allele PTPRC Is Also Associated with Response to Anti–Tumor Necrosis Factor $\alpha$ Therapy. *Arthritis & Rheumatism* **2010**, *62*, 1849–1861.
31. I. Acosta-Colman, N. Palau, J. Tornero, A. Fernández-Nebro, F. Blanco, I. González-Alvaro, J. D Cañete, J. Maymó, J. Ballina, B.Fernández-Gutiérrez, A.Olivé, H. Corominas, A. Erra, O. Canela-Xandri, Ar, A.J.& S.M. GWAS Replication Study Confirms the Association of PDE3A–SLCO1C1 with Anti-TNF Therapy Response in Rheumatoid Arthritis. *Pharmacogenomics* **2013**, *14*, 727–734.
32. Smith, S.L.; Plant, D.; Lee, X.H.; Massey, J.; Hyrich, K.; Morgan, A.W.; Wilson, A.G.; Isaacs, J.; Barton, A. Previously Reported PDE3A-SLCO1C1 Genetic Variant Does Not Correlate with Anti-TNF Response in a Large UK Rheumatoid Arthritis Cohort. *Pharmacogenomics* **2016**, *17*, 715–720, doi:10.2217/pgs.16.16.
33. Conigliaro, P.; Ciccacci, C.; Politi, C.; Triggianese, P.; Rufini, S.; Kroegler, B.; Perricone, C.; Latini, A.; Novelli, G.; Borgiani, P.; et al. Polymorphisms in STAT4, PTPN2, PSORS1C1 and TRAF3IP2 Genes Are Associated with the Response to TNF Inhibitors in Patients with Rheumatoid Arthritis. *PLoS ONE* **2017**, *12*, 1–14, doi:10.1371/journal.pone.0169956.
34. Sode, J.; Vogel, U.; Bank, S.; Andersen, P.S.; Hetland, M.L.; Locht, H.; Heegaard, N.H.H.; Andersen, V. Genetic Variations in Pattern Recognition Receptor Loci Are Associated with Anti-TNF Response in Patients with Rheumatoid Arthritis. *PLoS ONE* **2015**, *10*, 1–13, doi:10.1371/journal.pone.0139781.
35. Kang, C.P.; Lee, K.W.; Yoo, D.H.; Kang, C.; Bae, S.C. The Influence of a Polymorphism at Position -857 of the Tumour Necrosis Factor $\alpha$ Gene on Clinical Response to Etanercept Therapy in Rheumatoid Arthritis. *Rheumatology* **2005**, *44*, 547–552, doi:10.1093/rheumatology/keh550.
36. Miceli-Richard, C.; Comets, E.; Verstuyft, C.; Tamouza, R.; Loiseau, P.; Ravaud, P.; Kupper, H.; Becquemont, L.; Charron, D.; Mariette, X. A Single Tumour Necrosis Factor Haplotype Influences the Response to Adalimumab in Rheumatoid Arthritis. *Ann Rheum Dis* **2008**, *67*, 478–484, doi:10.1136/ard.2007.074104.
37. Pinto, J.A.; Rego, I.; Fernandez-López, C.; Freire, M.; Fernandez-Sueiro, J.L.; Blanco, F.J.; Rodríguez-Gomez, M.; Sanmarti, R.; Cañete, J.D. Polymorphisms in Genes Encoding Tumor Necrosis Factor-$\alpha$ and HLA-DRB1 Are Not Associated with Response to Infliximab in Patients with Rheumatoid Arthritis (Journal of Rheumatology (2008) 35, (177-178)). *Journal of Rheumatology* **2008**, *35*, 546.
38. Maxwel, J.R.; Potter, C.; Hyrich, K.L.; Barton, A.; Worthington, J.; Isaacs, J.D.; Morgan, A.W.; Wilson, A.G. Association of the Tumour Necrosis Factor-308 Variant with Differential Response to Anti-TNF Agents in the Treatment of Rheumatoid Arthritis. *Hum Mol Genet* **2008**, *17*, 3532–3538, doi:10.1093/hmg/ddn245.
39. Swierkot, J.; Bogunia-Kubik, K.; Nowak, B.; Bialowas, K.; Korman, L.; Gebura, K.; Kolossa, K.; Jeka, S.; Wiland, P. Analysis of Associations between Polymorphisms within Genes Coding for Tumour Necrosis Factor (TNF)-Alpha and TNF Receptors and Responsiveness to TNF-Alpha Blockers in Patients with Rheumatoid Arthritis. *Joint Bone Spine* **2015**, *82*, 94–99, doi:10.1016/j.jbspin.2014.08.006.

40. Jančić, I.; Šefik-Bukilica, M.; Živojinović, S.; Damjanov, N.; Spasovski, V.; Kotur, N.; Klaassen, K.; Pavlović, S.; Bufan, B.; Arsenović-Ranin, N. Influence of Promoter Polymorphisms of the Tnf-$\alpha$ (-308g/A) and IL-6 (-174g/C) Genes on Therapeutic Response to Etanercept in Rheumatoid Arthritis. *J Med Biochem* **2015**, *34*, 414–421, doi:10.2478/jomb-2014-0060.
41. Cuchacovich, M.; Soto, L.; Edwardes, M.; Gutierrez, M.; Llanos, C.; Pacheco, D.; Sabugo, F.; Alamo, M.; Fuentealba, C.; Villanueva, L.; et al. Tumour Necrosis Factor (TNF)$\alpha$ -308 G/G Promoter Polymorphism and TNF$\alpha$ Levels Correlate with a Better Response to Adalimumab in Patients with Rheumatoid Arthritis. *Scand J Rheumatol* **2006**, *35*, 435–440, doi:10.1080/03009740600904284.
42. Guis, S.; Balandraud, N.; Bouvenot, J.; Auger, I.; Toussirot, E.; Wendling, D.; Mattel, J.P.; Nogueira, L.; Mugnier, B.; Legeron, P.; et al. Influence of -308 A/G Polymorphism in the Tumor Necrosis Factor $\alpha$ Gene on Etanercept Treatment in Rheumatoid Arthritis. *Arthritis Care Res (Hoboken)* **2007**, *57*, 1426–1430, doi:10.1002/art.23092.
43. Marotte, H.; Arnaud, B.; Diasparra, J.; Zrioual, S.; Miossec, P. Association between the Level of Circulating Bioactive Tumor Necrosis Factor $\alpha$ and the Tumor Necrosis Factor $\alpha$ Gene Polymorphism at -308 in Patients with Rheumatoid Arthritis Treated with a Tumor Necrosis Factor $\alpha$ Inhibitor. *Arthritis Rheum* **2008**, *58*, 1258–1263, doi:10.1002/art.23430.
44. Vasilopoulos, Y.; Bagiatis, V.; Stamatopoulou, D.; Zisopoulos, D.; Alexiou, I.; Sarafidou, T.; Settas, L.; Sakkas, L.; Mamuris, Z. Association of Anti-CCP Positivity and Carriage of TNFRII Susceptibility Variant with Anti-TNF-$\alpha$ Response in Rheumatoid Arthritis. *Clin Exp Rheumatol* **2011**, *29*, 701–704.
45. Mugnier, B.; Balandraud, N.; Darque, A.; Roudier, C.; Roudier, J.; Reviron, D. Polymorphism at Position -308 of the Tumor Necrosis Factor $\alpha$ Gene Influences Outcome of Infliximab Therapy in Rheumatoid Arthritis. *Arthritis Rheum* **2003**, *48*, 1849–1852, doi:10.1002/art.11168.
46. Cuchacovich, M.; Ferreira, L.; Aliste, M.; Soto, L.; Cuenca, J.; Cruzat, A.; Gatica, H.; Schiattino, I.; Pérez, C.; Aguirre, A.; et al. Tumour Necrosis Factor-$\alpha$ (TNF-$\alpha$) Levels and Influence of -308 TNF-$\alpha$ Promoter Polymorphism on the Responsiveness to Infliximab in Patients with Rheumatoid Arthritis. *Scand J Rheumatol* **2004**, *33*, 228–232, doi:10.1080/03009740410005863.
47. Chatzikyriakidou, A.; Georgiou, I.; Voulgari, P. V.; Venetsanopoulou, A.I.; Drosos, A.A. Combined Tumour Necrosis Factor-$\alpha$ and Tumour Necrosis Factor Receptor Genotypes Could Predict Rheumatoid Arthritis Patients' Response to Anti-TNF-$\alpha$ Therapy and Explain Controversies of Studies Based on a Single Polymorphism [1]. *Rheumatology* **2007**, *46*, 1034–1035, doi:10.1093/rheumatology/kem041.
48. Morales-Lara, M.J.; Cañete, J.D.; Torres-Moreno, D.; Hernández, M.V.; Pedrero, F.; Celis, R.; García-Simón, M.S.; Conesa-Zamora, P. Effects of Polymorphisms in TRAILR1 and TNFR1A on the Response to Anti-TNF Therapies in Patients with Rheumatoid and Psoriatic Arthritis. *Joint Bone Spine* **2012**, *79*, 591–596, doi:10.1016/j.jbspin.2012.02.003.
49. Toonen, E.J.M.; Coenen, M.J.H.; Kievit, W.; Fransen, J.; Eijsbouts, A.M.; Scheffer, H.; Radstake, T.R.D.J.; Creemers, M.C.W.; De Rooij, D.J.R.A.M.; Van Riel, P.L.C.M.; et al. The Tumour Necrosis Factor Receptor Superfamily Member 1b 676T>G Polymorphism in Relation to Response to Infliximab and Adalimumab Treatment and Disease Severity in Rheumatoid Arthritis. *Ann Rheum Dis* **2008**, *67*, 1174–1177, doi:10.1136/ard.2008.088138.
50. Canet, L.M.; Filipescu, I.; Caliz, R.; Lupiañez, C.B.; Canhaõ, H.; Escudero, A.; Segura-Catena, J.; Soto-Pino, M.J.; Ferrer, M.A.; Garcia, A.; et al. Genetic Variants within the TNFRSF1B Gene and Susceptibility to Rheumatoid Arthritis and Response to Anti-TNF Drugs: A Multicenter Study. *Pharmacogenet Genomics* **2015**, *25*, 323–333, doi:10.1097/FPC.0000000000000140.
51. Márquez Pete, N.; Maldonado Montoro, M.D.M.; Pérez Ramírez, C.; Martínez Martínez, F.; Martínez de la Plata, J.E.; Daddaoua, A.; Jiménez Morales, A. Influence of the Fcgr2a Rs1801274 and Fcgr3a Rs396991 Polymorphisms on Response to Abatacept in Patients with Rheumatoid Arthritis. *Journal of Personalized Medicine* **2021**, *11*, 1–15, doi:10.3390/jpm11060573.

52. Gazeau, P.; Alegria, G.C.; Devauchelle-Pensec, V.; Jamin, C.; Lemerle, J.; Bendaoud, B.; Brooks, W.H.; Saraux, A.; Cornec, D.; Renaudineau, Y. Memory B Cells and Response to Abatacept in Rheumatoid Arthritis. *Clinical Reviews in Allergy and Immunology* **2017**, *53*, 166–176, doi:10.1007/s12016-017-8603-x.
53. Ruyssen-Witrand, A.; Rouanet, S.; Combe, B.; Dougados, M.; Loët, X. Le; Sibilia, J.; Tebib, J.; Mariette, X.; Constantin, A. Association between -871C>T Promoter Polymorphism in the B-Cell Activating Factor Gene and the Response to Rituximab in Rheumatoid Arthritis Patients. *Rheumatology (United Kingdom)* **2013**, *52*, 636–641, doi:10.1093/rheumatology/kes344.
54. Fabris, M.; Quartuccio, L.; Vital, E.; Pontarini, E.; Salvin, S.; Fabro, C.; Zabotti, A.; Benucci, M.; Manfredi, M.; Ravagnani, V.; et al. The TTTT B Lymphocyte Stimulator Promoter Haplotype Is Associated with Good Response to Rituximab Therapy in Seropositive Rheumatoid Arthritis Resistant to Tumor Necrosis Factor Blockers. *Arthritis and Rheumatism* **2013**, *65*, 88–97, doi:10.1002/art.37707.
55. Kastbom, A.; Cöster, L.; Ärlestig, L.; Chatzidionysiou, A.; Van Vollenhoven, R.F.; Padyukov, L.; Rantapää-Dahlqvist, S.; Saevarsdottir, S. Influence of FCGR3A Genotype on the Therapeutic Response to Rituximab in Rheumatoid Arthritis: An Observational Cohort Study. *BMJ Open* **2012**, *2*, 1–4, doi:10.1136/bmjopen-2012-001524.
56. Pál, I.; Szamosi, S.; Hodosi, K.; Szekanecz, Z.; Váróczy, L. Effect of Fcγ-Receptor 3a (FCGR3A) Gene Polymorphisms on Rituximab Therapy in Hungarian Patients with Rheumatoid Arthritis. *RMD Open* **2017**, *3*, 1–4, doi:10.1136/rmdopen-2017-000485.
57. Jiménez Morales, A.; Maldonado-Montoro, M.; Martínez de la Plata, J.E.; Pérez Ramírez, C.; Daddaoua, A.; Alarcón Payer, C.; Expósito Ruiz, M.; García Collado, C. FCGR2A/FCGR3A Gene Polymorphisms and Clinical Variables as Predictors of Response to Tocilizumab and Rituximab in Patients With Rheumatoid Arthritis. *Journal of Clinical Pharmacology* **2019**, *59*, 517–531, doi:10.1002/jcph.1341.
58. Quartuccio, L.; Fabris, M.; Pontarini, E.; Salvin, S.; Zabotti, A.; Benucci, M.; Manfredi, M.; Biasi, D.; Ravagnani, V.; Atzeni, F.; et al. The 158VV Fcgamma Receptor 3A Genotype Is Associated with Response to Rituximab in Rheumatoid Arthritis: Results of an Italian Multicentre Study. *Annals of the Rheumatic Diseases* **2014**, *73*, 716–721, doi:10.1136/annrheumdis-2012-202435.
59. Ruyssen-Witrand, A.; Rouanet, S.; Combe, B.; Dougados, M.; Le Loët, X.; Sibilia, J.; Tebib, J.; Mariette, X.; Constantin, A. Fcγ Receptor Type IIIA Polymorphism Influences Treatment Outcomes in Patients with Rheumatoid Arthritis Treated with Rituximab. *Annals of the Rheumatic Diseases* **2012**, *71*, 875–877, doi:10.1136/annrheumdis-2011-200337.
60. Daïen, C.I.; Fabre, S.; Rittore, C.; Soler, S.; Daïen, V.; Tejedor, G.; Cadart, D.; Molinari, N.; Daurès, J.P.; Jorgensen, C.; et al. TGF Beta1 Polymorphisms Are Candidate Predictors of the Clinical Response to Rituximab in Rheumatoid Arthritis. *Joint Bone Spine* **2012**, *79*, 471–475, doi:10.1016/j.jbspin.2011.10.007.
61. Juge, P.A.; Gazal, S.; Constantin, A.; Mariette, X.; Combe, B.; Tebib, J.; Dougados, M.; Sibilia, J.; Le Loet, X.; Dieudé, P. Variants of Genes Implicated in Type 1 Interferon Pathway and B-Cell Activation Modulate the EULAR Response to Rituximab at 24 Weeks in Rheumatoid Arthritis. *RMD Open* **2017**, *3*, 1–5, doi:10.1136/rmdopen-2017-000448.
62. Luxembourger, C.; Ruyssen-Witrand, A.; Ladhari, C.; Rittore, C.; Degboe, Y.; Maillefert, J.F.; Gaudin, P.; Marotte, H.; Wendling, D.; Jorgensen, C.; et al. A Single Nucleotide Polymorphism of IL6-Receptor Is Associated with Response to Tocilizumab in Rheumatoid Arthritis Patients. *Pharmacogenomics Journal* **2019**, *19*, 368–374, doi:10.1038/s41397-019-0072-6.
63. Enevold, C.; Baslund, B.; Linde, L.; Josephsen, N.L.; Tarp, U.; Lindegaard, H.; Jacobsen, S.; Nielsen, C.H. Interleukin-6-Receptor Polymorphisms Rs12083537, Rs2228145, and Rs4329505 as Predictors of Response to Tocilizumab in Rheumatoid Arthritis. *Pharmacogenetics and Genomics* **2014**, *24*, 401–405, doi:10.1097/FPC.0000000000000071.
64. Maldonado-Montoro, M.; Cañadas-Garre, M.; González-Utrilla, A.; Ángel Calleja-Hernández, M. Influence of IL6R Gene Polymorphisms in the Effectiveness to Treatment with Tocilizumab in Rheumatoid Arthritis. *Pharmacogenomics Journal* **2018**, *18*, 167–172, doi:10.1038/tpj.2016.88.
